# Gastric morphology in sigmodontine rodents (Mammalia: Cricetidae): a comprehensive comparative classification

**DOI:** 10.7717/peerj.21405

**Published:** 2026-06-10

**Authors:** Ulyses F. J. Pardiñas, Jorge Brito, Carola Cañón, Jorge Cherem, Erika Cuellar Soto, Marisol Hidalgo-Cossio, Nuria Bernal-Hoverud, Belkis Rivas, Raisa Cairampoma, João Alves de Oliveira

**Affiliations:** 1 Instituto de Diversidad y Evolucion Austral, Consejo Nacional de Investigaciones Científicas y Técnicas, Puerto Madryn, Chubut, Argentina; 2 Instituto Nacional de Biodiversidad (INABIO), Quito, Ecuador; 3 Centro Internacional Cabo de Hornos (CHIC), Puerto Williams, Chile; 4 Instituto Milenio Centro de Regulación del Genoma (CRG), Santiago, Chile; 5 Instituto Tabuleiro, Florianópolis, Brazil; 6 Department of Biology, College of Science, Sultan Qaboos University, Muscat, Oman; 7 Colección Boliviana de Fauna, Museo Nacional de Historia Natural, La Paz, Bolivia; 8 Museo de Historia Natural Alcide d’Orbigny, Cochabamba, Bolivia; 9 Department of Biological Sciences, Texas Tech University, Lubbock, United States; 10 Grupo de Ecología Animal, Facultad de Ciencias, Universidad de Los Andes, Merida, Venezuela; 11 Departamento de Mastozoología, Museo de Historia Natural, Universidad Nacional Mayor de San Marcos, Lima, Peru; 12 Museu Nacional, Universidade Federal do Rio de Janeiro, Rio de Janeiro, Brazil

**Keywords:** Comparative anatomy, Digestive system, Stomach

## Abstract

Sigmodontine rodents (Cricetidae: Sigmodontinae) represent the most diverse extant mammalian subfamily in both generic and species richness. Variation in conservative organ systems, such as the stomach, can provide insight into the morphological basis of their ecological diversification. Here, we examined the gross internal anatomy of 602 stomachs representing 168 species, 74 genera, and 13 tribes—approximately 80% of the subfamily’s generic diversity—using sagittal and transverse sections. We propose a refined and standardized classification of stomach morphology based on the distribution of glandular and cornified epithelia. Five principal gastric designs are recognized: equiglandular, supraglandular, subglandular, discoglandular, and diverticular. The equiglandular condition predominates, occurring in approximately 65% of genera. Most tribes are characterized by a single gastric design, whereas Akodontini and Thomasomyini each exhibit multiple configurations. Notable exceptions include *Phaenomys*, which departs from the typical tribal pattern, and *Thomasomys*, the only genus exhibiting more than one stomach configuration. Ancestral-state reconstruction supports the equiglandular condition as the primitive state for Sigmodontinae and their sister clade, Tylomyinae. Independent origins of the diverticular condition in Akodontini and Thomasomyini indicate convergence, although detailed anatomical comparisons reveal distinct structural patterns. In addition, previously overlooked features—particularly the configuration of the bordering fold and its relationship to the esophageal opening and plica angularis—provide new insight into stomach organization. Overall, sigmodontine gastric morphology is structured across multiple hierarchical levels, combining strong phylogenetic signal with repeated evolutionary innovation. These results establish a comparative framework for interpreting stomach diversity and highlight the need for integrative approaches to fully understand digestive-system evolution in rodents.

## Introduction

The Neotropical mammalian fauna has long fascinated naturalists because of its distinctiveness. This interest dates back to early European explorations, with notable milestones including the work of Darwin (1839), who was inspired by Pampean fossils and Galápagos diversity—key evidence shaping his evolutionary ideas—and that of Attenborough (2018), who devoted much of his early work to Chacoan armadillos. However, the uniqueness of Neotropical assemblages extends beyond morphological or ecological attributes. The region harbors one of the most diverse extant mammalian radiations: the sigmodontine rodents (*e.g*., Patton, Pardiñas & D’Elía, 2015; Pardiñas et al., 2017; Brito & Pardiñas, 2025).

Whether measured by generic richness (91 genera, excluding recently extinct and fossil forms) or by species richness (around 500 species; Brito & Pardiñas, 2025), the Sigmodontinae rival—or even surpass—the Murinae in terms of evolutionary expansion (*e.g*., Denys, Taylor & Aplin, 2017). Despite their current diversity, sigmodontines appear to have a relatively recent evolutionary history compared to other major rodent lineages (*e.g.,* López-Antoñanzas et al., 2024), with their radiation generally estimated to have occurred during the late Miocene to Pliocene (ca. 8–6 million years ago; *e.g*., Aghová et al., 2018; Leite et al., 2014; Pardiñas et al., 2022; Bangs et al., 2025).

Although the group exhibits considerable morphological variation, particularly at the intertribal level in external, cranial, and ecological traits, several anatomical features remain stable across the subfamily. These include, among others, a complex (*i.e*., tridigitate) penile morphology, a molar pattern composed of three free-cemented teeth decreasing in size posteriorly, the “ICAMER” configuration of occlusal molar structures, the insertion of the nuchal ligament at the second thoracic vertebra, the parallel-type malleus, the unilocular stomach, and three complete diastemic rugae (*e.g*., Hooper & Musser, 1964; Carleton, 1973, 1980; Reig, 1977; Vorontsov, 1982; Voss, 1988; Barbière et al., 2019). This contrast between intertribal variability and conservatism in key structural features highlights the challenge of identifying morphological innovations that might have contributed to the successful radiation of the group. Moreover, any attempt to pinpoint such drivers requires broad and representative taxonomic sampling.

Among the organs of the digestive system, the stomach remains the only structure to have received moderate anatomical attention in sigmodontines. The most comprehensive study remains Carleton’s (1973) survey of approximately 34 genera. Despite the significance of this foundational work and a few additional contributions (*e.g*., Vorontsov, 1967; Dorst, 1972; Pardiñas et al., 2020), stomach morphology remains undescribed for a substantial portion of sigmodontine diversity. Furthermore, previous observations have not been reinterpreted in light of current phylogenetic frameworks, which now recognize three principal lineages within the subfamily: two composed of single, highly specialized tribes (Ichthyomyini and Sigmodontini), and a third, Oryzomyalia, comprising at least 11 suprageneric clades (*e.g*., Steppan, Adkins & Anderson, 2004; Leite et al., 2014; Pardiñas et al., 2022). This phylogenetic restructuring provides an essential framework for reassessing morphological traits, including stomach organization, in an evolutionary context.

Stimulated in part by the anatomical investigations of Vorontsov (1959, 1962, 1967), Carleton (1973, 1981) who undertook a foundational study of stomach morphology in cricetid rodents. Focusing primarily on the internal distribution of two epithelial types—cornified and glandular—he recognized two major patterns. The hemiglandular type presents a more or less equal coverage of the lumen by both epithelia, while the discoglandular type is characterized by glandular epithelium restricted to the fundus. In addition, Carleton (1973) examined the depth of the incisura angularis—a key fold in the anterior stomach wall—leading to a further dichotomy. A deeply penetrant incisura gives the stomach a bilocular (two-chambered) appearance, while a shallow expression results in a unilocular (single-chambered) configuration. By combining these characters, Carleton (1973) proposed not only a novel anatomical classification but also its potential systematic implications. This was particularly relevant at a time when the tribal arrangement of American cricetids was still under active debate (*e.g*., Hershkovitz, 1966; Hooper & Musser, 1964; Reig, 1972; Gardner & Patton, 1976; Pearson & Patton, 1976). Sigmodontinae and Tylomyinae were considered to possess primarily unilocular-hemiglandular stomachs, whereas Neotominae were characterized by bilocular-discoglandular types. After more than half a century, Carleton’s (1973) framework remains central to describing the gross anatomy of the cricetid stomach (Langer, 2017). However, recent studies focusing on previously overlooked structures (*e.g*., bordering fold, pars pylorica) have introduced refinements to Carleton’s scheme (*e.g*., Pardiñas et al., 2020). Moreover, although the descriptive terms proposed by Carleton (1973) are applicable beyond cricetids, a partially inconsistent and often conflicting terminology persists in the literature—even within muroid rodents (*e.g*., Langer, 2017; Walters et al., 2014; Steiner et al., 2022). Despite its enduring influence, this framework has not been systematically revisited using expanded taxonomic sampling and modern phylogenetic hypotheses.

Beyond its descriptive value, stomach morphology represents a key interface between diet, physiology, and ecological specialization in rodents (*e.g*., Carleton, 1973; Vorontsov, 1982; Langer, 2017). As such, variation in its internal organization may provide insights into functional adaptation and the evolutionary diversification of lineages (*e.g*., Langer, 2002; Langer & Clauss, 2018). In sigmodontine rodents—one of the most rapidly diversified mammalian radiations—understanding patterns of stomach morphology may help clarify whether anatomical diversification has accompanied their ecological and phylogenetic expansion. In particular, evaluating stomach morphology within a phylogenetic framework allows testing whether its variation reflects evolutionary history, convergent ecological adaptation, or a combination of both Vorontsov (1982).

This study proposes a revised classification of stomach morphology in sigmodontine rodents, based on extensive comparative anatomical data and interpreted within a phylogenetic framework. We also establish a consistent and refined terminology for describing the internal organization of the stomach. By integrating broad taxonomic sampling with evolutionary analyses, we aim to provide a framework for understanding the diversification and functional significance of stomach morphology within this radiation. We further explore whether major patterns of stomach organization are associated with phylogenetic structure or instead reflect repeated ecological convergence. To achieve this, we adopted a collaborative approach involving researchers from multiple countries, allowing us to sample nearly 80% of all extant genera—including most morphologically distinctive forms. In addition, this study focuses on internal features of the stomach that have previously received little or no attention, providing new insights into its anatomical diversity, evolutionary patterns, and potential functional significance.

## Materials and Methods

### Studied specimens

Examined specimens (mostly adults) consisted of fluid-preserved bodies, usually fixed in 10% formalin and preserved in 70% ethanol, from which the entire digestive system was removed. The complete list of material examined (602 stomachs representing 168 species, 74 genera, and 13 tribes) is summarized in Table 1 and provided in detail in Table S1.

**Table 1:** Total number of tribes, genera, and species of Sigmodontinae for which stomach morphology was examined in this study.

Tribe	Subtribe/Clade	Genus	Species
Abrotrichini	Abrotrichina	*Abrothrix*	4
	Notyomyina	*Geoxus*	1
		*Notiomys*	1
		*Paynomys*	1
Akodontini	Akodontina	*Akodon*	14
		*Castoria*	1
		*Deltamys*	1
		*Microxus*	1
		*Necromys*	5
		*Thalpomys*	2
		*Thaptomys*	1
	Oxymycterina	*Juscelinomys*	1
		*Oxymycterus*	6
	Scapteromyina	*Bibimys*	2
		*Blarinomys*	1
		*Brucepattersonius*	1
		*Kunsia*	1
		*Lenoxus*	1
		*Scapteromys*	1
Andinomyini		*Andinomys*	1
		*Punomys*	1
Euneomyini		*Euneomys*	1
		*Irenomys*	1
		*Neotomys*	1
Ichthyomyini	Ichthyomyina	*Daptomys*	1
		*Ichthyomys*	5
		*Neusticomys*	2
Neomicroxini		*Neomicroxus*	1
Oryzomyini	Clade A	*Scolomys*	1
		*Zygodontomys*	1
	Clade B	*Casiomys*	2
		*Euryoryzomys*	2
		*Hylaeamys*	3
		*Mindomys*	1
		*Nephelomys*	3
		*Oecomys*	2
		*Pattonimus*	2
		*Transandinomys*	2
	Clade C	*Microryzomys*	2
		*Neacomys*	2
		*Oligoryzomys*	5
		*Oreoryzomys*	1
	Clade D	*Aegialomys*	1
		*Cerradomys*	1
		*Holochilus*	3
		*Lundomys*	1
		*Melanomys*	2
		*Nectomys*	2
		*Pseudoryzomys*	1
		*Sigmodontomys*	1
		*Sooretamys*	1
		*Tanyuromys*	1
Phyllotini	Calomyina	*Calassomys*	1
		*Calomys*	5
	Phyllotina	*Auliscomys*	3
		*Eligmodontia*	4
		*Graomys*	2
		*Loxodontomys*	1
		*Phyllotis*	7
		*Tapecomys*	1
Reithrodontini		*Reithrodon*	1
Rhagomyini		*Rhagomys*	2
Sigmodontini		*Sigmodon*	1
Thomasomyini		*Aepeomys*	1
		*Chilomys*	4
		*Rhipidomys*	4
		*Thomasomys*	18
Wiedomyini		*Juliomys*	2
		*Phaenomys*	1
		*Wiedomys*	2
		*Wilfredomys*	1
Incertae sedis		*Abrawayaomys*	1
		*Chinchillula*	1
		*Delomys*	2
Total = 13		74	168

Some taxa could not be examined directly and were instead assessed using published descriptions and illustrations. These include eight genera (*Anotomys* [assessed after Voss, 1988], *Chibchanomys* [Voss, 1988], *Drymoreomys* [Percequillo, Weksler & Costa, 2011], *Handleyomys* [Voss, Gómez-Laverde & Pacheco, 2002], *Incanomys* [Zeballos et al., 2025], *Nesoryzomys* [Patton & Hafner, 1983], *Oryzomys* [Carleton & Musser, 1989], and *Rheomys* [Carleton, 1973]). In addition, at least five genera (*Amphinectomys*, *Chelemys*, *Galenomys*, *Gyldenstolpia*, and *Microakodontomys*) are currently known to lack preserved digestive systems in museum collections, to the best of our knowledge.

Acronyms for collections cited in figures are as follows: CBF, Colección Boliviana de Fauna, La Paz, Bolivia; CNP, Colección de Mamíferos del Centro Nacional Patagónico, Chubut, Argentina; CNP-D, Colección de Mamíferos (anexo digestivos) del Centro Nacional Patagónico, Chubut, Argentina; CML, Colección Mamíferos Lillo, Tucumán, Argentina; CMUFLA, Colección de Mamíferos de la Universidade Federal de Lavras, Minas Gerais, Brazil; CMZ, Colección del Museo de Zoología “Alfonso Herrera,” Universidad Nacional Autónoma de México, Mexico; CVULA, Colección de Vertebrados, Universidad de Los Andes, Mérida, Venezuela; MECN, Colección de Mastozoología del Instituto Nacional de Biodiversidad (INABIO), Quito, Ecuador; MN, Museu Nacional, Rio de Janeiro, Brazil; MUSA, Museo de Historia Natural, Universidad Nacional de San Agustín, Arequipa, Peru; MZUFV, Museu de Zoologia, Departamento de Biologia Animal, Universidade Federal de Viçosa, Viçosa, Minas Gerais, Brazil; QCAZ, Museo de Zoología de la Pontificia Universidad Católica del Ecuador, Quito, Ecuador; and UFSC, Universidade Federal de Santa Catarina, Florianópolis, Santa Catarina, Brazil.

### Anatomy and terminology

**Figure 1:**
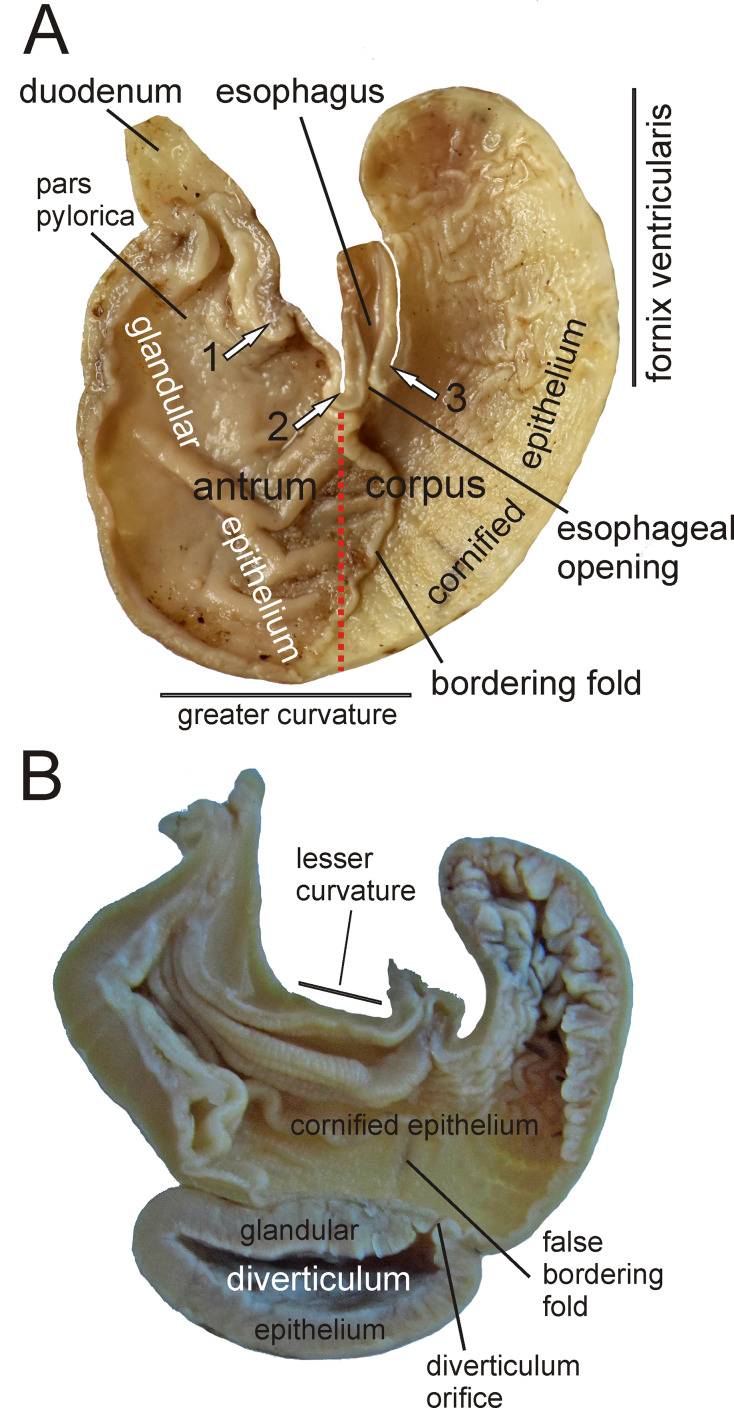
Overall stomach anatomy and terminology in Sigmodontinae. Ventral view of a midfrontal section of *Scolomys melanops* (A; MECN 5777) and *Brucepattersonius iheringi* (B; CNP 5507). Numbered labels indicate anatomical features: 1, incisura praepylorica; 2, incisura angularis; 3, incisura cardialis. In all stomach photographs presented in this study, the *corpus* is positioned to the right (anatomically to the left). All figures are shown at approximately the same scale. Photographs by J. Brito (A) and U. Pardiñas (B).

Stomachs were photographed externally in ventral view and then bisected along the midsagittal plane to expose the internal gross anatomy, after the food contents were washed out (Fig. 1). Observations were conducted under stereomicroscope magnification, focusing primarily on the surface appearance of structures and epithelial regions. Selected specimens were processed histologically; however, neither cellular- nor tissue-level details, nor functional interpretations, are included in this article.

During the final stages of this study, after several hundred stomachs had been opened following the basic procedure described above, a methodological problem with this approach was identified, having been perpetuated since Tullberg (1899). If the incision is not perfectly aligned with the midsagittal plane, or if the organ is not fixed without deformation, the resulting internal morphology may exhibit spurious variation. In addition, and partially related to this effect, the regions of the stomach most affected are those surrounding the esophageal opening, the incisura angularis, and the area where the bordering fold meets the lesser curvature.

**Figure 2:**
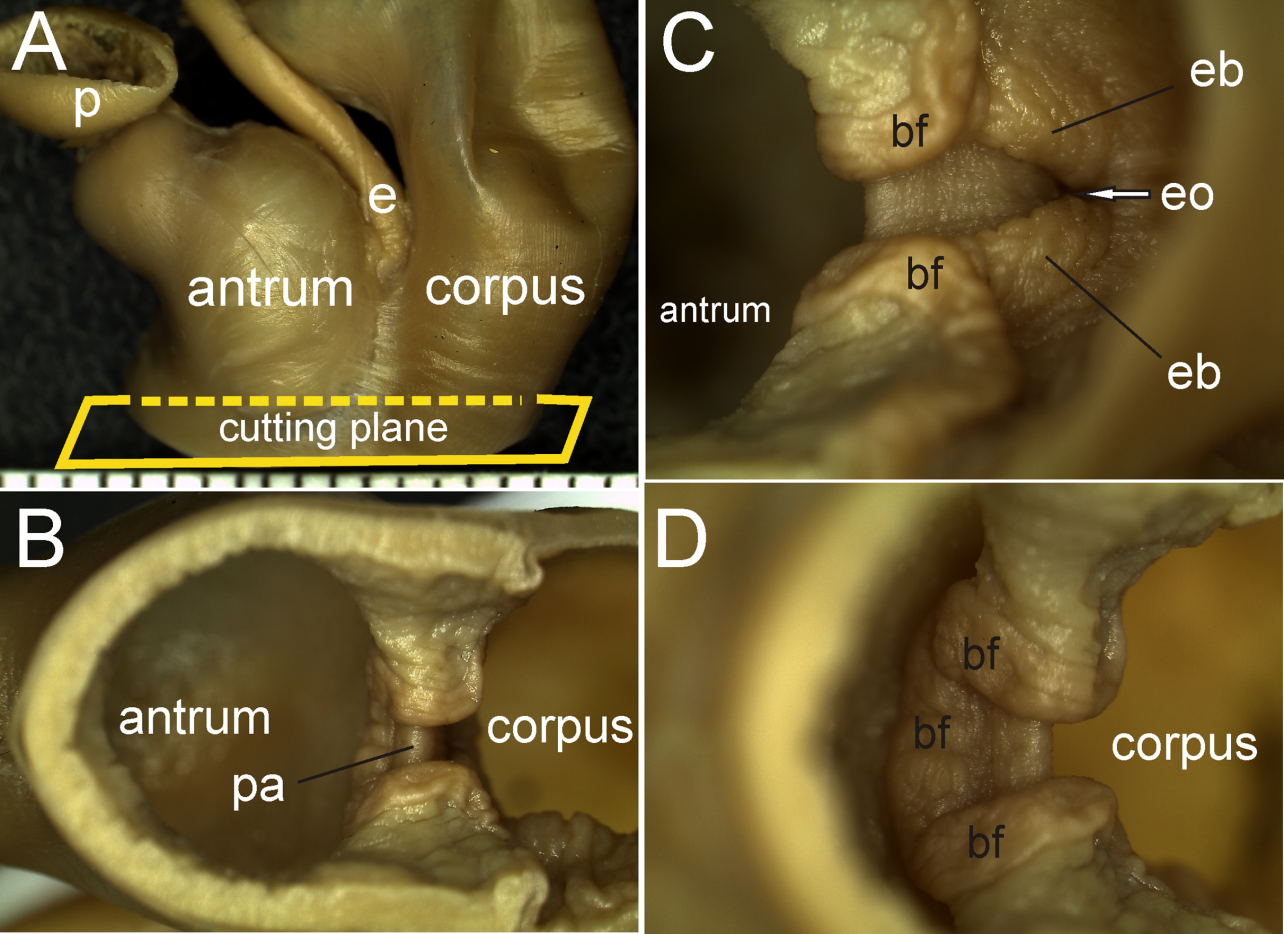
Novel cutting approach to expose internal stomach morphology. (A) External view of the stomach showing the cutting plane. (B) Internal overview of the anterior half. (C) Internal view tilted slightly to the right to expose the esophageal opening. (D) Internal view tilted slightly to the left to show the bordering fold (*Abrothrix olivacea*; CNP 6664). Abbreviations: bf, bordering fold; e, esophagus; eb, esophageal bordering fold; eo, esophageal opening; p, pylorus; pa, plica angularis. All figures are shown at approximately the same scale. Photographs by U. Pardiñas.

To overcome this issue, a new opening procedure was applied to selected specimens, consisting of a transverse cut approximately at the middle of the organ (Fig. 2). This procedure produces two portions: an anterior (cranial) and a posterior (caudal) region. Internal inspection of the anterior portion provides a novel perspective for assessing a critical anatomical region that has been largely overlooked due to traditional methodological practices. This region includes the transition from the esophageal to the cardiac stomach and part of the cardiac stomach *sensu* Blank (1950, p. 425, fig. 1). The main features examined are the position of the esophageal opening, the nature of the surrounding epithelium, and, most importantly, the configuration of the bordering fold as it interacts with the esophageal opening and the incisura angularis.

The basic anatomical terminology employed in this study follows Carleton (1973, 1981). Additional nomenclature required to describe certain traits was derived from Vorontsov (1967) or is introduced here. All terms used are illustrated in Figs. 1 and 2 and are briefly described below (listed in alphabetical order):

Antrum: the part of the stomach to the right of the incisura angularis.

Bordering fold: a ridge of tissue marking the junction between glandular and cornified epithelia.

Cornified (or squamous) epithelium: one of the two main types of mucosal lining evident at the gross anatomical level, characterized by thin walls and a corrugated surface typically crossed by accordion-like folds.

*Corpus*: the part of the stomach to the left of the incisura angularis.

Diverticulum orifice: a minute opening associated with a very short channel that perforates the thick glandular wall, connecting the diverticulum with the main lumen of the stomach.

Diverticulum: a pouch containing glandular epithelium in diverticular stomachs, easily recognized by its external position relative to the main lumen and its thick walls.

Esophageal bordering fold: a prominent ridge—appearing as a double flap converging toward the esophageal opening—formed at the junction between the cornified epithelium and the gastric portion of the esophageal epithelium.

Esophageal opening: the aperture where the esophagus opens into the stomach.

False bordering fold: a ridge, usually faint but sometimes distinct, dividing two portions of the cornified epithelium that differ in surface appearance, typically aligned with the main axis of the stomach.

Fornix ventricularis: the part of the *corpus* extending cranially beyond the level of the gastroesophageal junction.

Glandular epithelium: one of the two kinds of mucosal lining evident at the gross anatomical level, characterized by thick walls and a surface that appears smooth or gently folded.

Incisura angularis (angular incisura): the angle formed by the dextral junction of the esophagus and stomach.

Incisura cardialis (cardial incisura): the angle formed by the sinistral junction of the esophagus and stomach.

Incisura praepylorica (prepyloric incisura): an angular indentation on the anterior surface of the stomach, situated to the right of the incisura angularis.

Isthmus: a medial, caudal indentation of the stomach associated with an internal band (sometimes more than one) of particularly corrugated cornified epithelium crossing the internal surface to the left of the bordering fold.

Pars pylorica: the portion of the stomach preceding the pylorus and typically delimited by the incisura praepylorica.

Plica angularis: the internal fold corresponding to the incisura angularis.

### Phylogenetic framework

Currently, two major groups of phylogenetic reconstructions are available for sigmodontines. Over the past 25 years, numerous studies have produced phylogenies based on broad taxonomic coverage (usually over 40 genera) but using limited molecular datasets—typically one mitochondrial marker (usually cytochrome b), alone or combined with one to five nuclear markers (*e.g*., Smith & Patton, 1999; D’Elía et al., 2006a; Salazar-Bravo, Pardiñas & D’Elía, 2013; Steppan & Schenk, 2017; Gonçalves et al., 2020; Pardiñas et al., 2022, 2024). In partial contrast, a genomic study using ultraconserved elements explored the genealogical relationships of 36 genera through a coalescent-based approach that included 2,958 characters (Parada, Hanson & D’Elía, 2021). The topologies recovered by these analyses are largely congruent. Two major clades characterize the base of the tree: one (Sigmodontalia, *sensu* Leite et al., 2014) includes two tribes, Ichthyomyini and Sigmodontini, while the other (Oryzomyalia, *sensu* Steppan, Adkins & Anderson, 2004) comprises the remaining lineages of the subfamily (at least 11 tribes plus three genera incertae sedis).

The principal differences among existing reconstructions involve support values for certain nodes that define tribal relationships within Oryzomyalia. However, these discrepancies are relatively limited. For example, the sister relationship between the largest sigmodontine tribe, Oryzomyini, and the remaining members of Oryzomyalia is well supported in several studies (*e.g*., Pardiñas et al., 2022 and references therein). In addition, several “classical” sister pairs are commonly recovered, such as Abrotrichini + Phyllotini (the latter often including the incertae sedis genus Delomys; *e.g*., Gonçalves et al., 2020) and Akodontini + Thomasomyini (the latter often including Rhagomyini; *e.g*., Pardiñas et al., 2022).

A second genomic study (Bangs et al., 2025) was recently published and is relevant here. Its reconstructed topology does not differ substantially from previous proposals. Bangs et al. (2025) proposed two new monotypic tribes to accommodate former incertae sedis genera—Chinchillulini (including Chinchillula) and Delomyini (including Delomys)—and assigned Abrawayaomys (also formerly incertae sedis) to Akodontini. Although these hypotheses are plausible, they will require further study to be confirmed or revised.

Within this context, and for the analysis of stomach morphological evolution, we followed the most taxonomically comprehensive and up-to-date phylogeny (Pardiñas et al., 2024), while constraining certain nodes according to the sister relationships recovered by Parada, Hanson & D’Elía (2021). In addition, some of the hypotheses proposed by Bangs et al. (2025) are briefly discussed in light of the findings on gastric anatomy.

### Phylogenetic analyses

A discrete character matrix representing the principal gastric morphotypes of Sigmodontinae was assembled based on direct examination of specimens, supplemented with published descriptions, and covering 83 of the 91 extant genera of the subfamily (Table S2). Each genus was assigned to one of the stomach morphotypes recognized in the refined classification proposed herein: discoglandular, diverticular, equiglandular, subglandular, and supraglandular (see Results). Direct observations were available for 74 genera; an additional eight genera were coded based on published sources (*i.e*., Voss, 1988; Zeballos et al., 2025; Carleton, 1973; Voss, Gómez-Laverde & Pacheco, 2002; Percequillo, Weksler & Costa, 2011; Patton & Hafner, 1983; Carleton & Musser, 1989). Eight genera were coded as missing because no specimens or published descriptions of gastric morphology are available. One genus, *Thomasomys*, was coded as polymorphic because different species exhibit either the equiglandular or subglandular morphotype.

Ancestral-state analyses were conducted using an updated version of the multilocus phylogeny derived from mitochondrial (cytb) and nuclear (IRBP, GHR, RAG1) sequences assembled by Pardiñas et al. (2024). The combined DNA matrix comprised 5,142 bp from four non-linked loci: cytb (1,159 bp), IRBP (1,165 bp), GHR (1,777 bp), and RAG1 (1,041 bp). The mitochondrial gene cytb was best fitted by a GTR+G model, whereas the nuclear genes IRBP, GHR, and RAG1 were best fitted by HKY+G models. The dataset includes all sigmodontine genera for which sequence data are publicly available (excluding *Gyldenstolpia*) and incorporates 19 outgroup taxa representing additional cricetid subfamilies and more distantly related muroids (Table S2). Sequences were curated and aligned independently for each locus using ClustalX (Larkin et al., 2007).

Phylogenetic inference followed a Maximum Likelihood framework implemented in IQ-TREE v2.2 (Minh et al., 2020). Genes were treated as independent partitions to account for locus-specific evolutionary heterogeneity (*e.g*., differences in substitution rates and selective constraints), rather than grouping markers by genomic origin (mitochondrial *vs*. nuclear), which may obscure such variation. The best-fitting substitution model for each partition was selected using ModelFinder in IQ-TREE. Nodal support was assessed using ultrafast bootstrap analyses with 1,000 replicates.

Ancestral stomach morphotypes were inferred in Mesquite v3.7 (Maddison & Maddison, 2019) using the discrete morphotype matrix (one multistate character per genus) and the Maximum Likelihood phylogeny. Reconstructions followed the Maximum Likelihood method under the Mk1 model (Markov k-state model with equal transition probabilities among states). For all internal nodes, likelihood values were obtained for each alternative state, and the corresponding probabilities were visualized across the phylogeny using proportional-symbol diagrams. Analyses were conducted under default optimization settings for 20 million iterations, sampling every 1,000 steps to ensure convergence.

### Background

Although early references to the stomach morphology of sigmodontines appear in older literature (*e.g*., Cuvier, 1837), the comparative study of Rodentia conducted by Tullberg (1899) is likely the first work in which two members of this subfamily were examined. Despite working with limited material—four poorly preserved *Sigmodon* specimens and one *Oxymycterus*—Tullberg (1899) documented the markedly different distribution of cornified and glandular epithelia in these two genera (Tullberg, 1899: plate XLI, figs. 22–24). The pouched condition of the glandular epithelium in the stomach of *Oxymycterus* was particularly notable, warranting both an illustration of its internal aspect (Tullberg, 1899: plate XLI, fig. 24) and a discussion of its possible connection to similar structures in other muroids (*e.g*., *Lophuromys*; see Tullberg, 1899: 451–452).

Beyond a few specific studies on *Sigmodon* (*e.g*., Blank, 1950)—a genus frequently addressed due to its occurrence in the United States—it was Vorontsov (1959, 1962, 1967, 1979, 1982) who initiated the modern study of digestive system morphology in rodents. His contributions synthesized data from the entire digestive tract, from lips to anus, based on both original dissections and literature sources. In the initial phase of his work, Vorontsov (1962, 1967) examined genera such as *Akodon*, *Nectomys*, *Oryzomys*, and *Oxymycterus*, providing schematic illustrations for most and a detailed drawing for the latter (Vorontsov, 1967: fig. 100; Fig. S1). *Sigmodon* was briefly mentioned (Vorontsov, 1967: 133). In a later synthesis, Vorontsov (1982) incorporated much of the data produced by Carleton (1973). Although Vorontsov’s work (1982 and references therein) substantially advanced understanding of digestive system evolution in rodents, its impact on sigmodontine comparative anatomy was limited by the small number of taxa examined and its focus on the basic distribution of internal epithelia.

Carleton (1973) conducted the foundational study on gross stomach morphology in sigmodontines. This work was notable not only for its taxonomic breadth—equivalent to approximately 71 species, 34 genera, and nine tribes under current classifications—but also for its explicit phylogenetic framework. Carleton (1973: 9) introduced a standardized method for illustrating stomach morphology: “Excised stomachs were bisected along a plane horizontal with the longitudinal body axis. This plane of section provided the most information for illustrative purposes. Figured stomachs thus appear in conventional anatomical position with the ventral half cut away.” This approach became standard in subsequent studies and remains widely used in analyses of sigmodontine stomachs (*e.g*., Langer, 2017; Pardiñas et al., 2020). However, this convention has also limited the examination of certain internal structures (see Results).

Carleton (1973) also established or refined a set of anatomical terms—incisura angularis, *corpus*, antrum, fornix ventricularis, and bordering fold—to describe internal stomach features. This mixed Latin–English terminology, though not entirely new (cf. Vorontsov, 1967), contributed to standardizing previously inconsistent nomenclature (*e.g*., cardiac, esophageal, fundic, and pyloric regions; see Blank, 1950; Grassé & Dekeyser, 1955).

Two major anatomical designs were identified by Carleton (1973: 10): unilocular-hemiglandular and bilocular-discoglandular. These were distinguished based on the depth of the *incisura angularis* and the distribution of cornified and glandular epithelia. In unilocular-hemiglandular stomachs, the *incisura angularis* does not penetrate deeply, resulting in a single-chambered appearance, with cornified and glandular epithelia each covering roughly half of the internal surface. In contrast, bilocular-discoglandular stomachs have a deeply penetrating *incisura angularis*, producing a double-chambered appearance, with glandular epithelium restricted to a disc-shaped area along the greater curvature. This classification, with minor modifications, has been retained in more recent treatments (Langer, 2017: 199–202). Carleton (1973: fig. 12) also incorporated stomach morphology into an evolutionary framework, proposing that taxa with reduced glandular epithelium represent derived forms evolving from an ancestral hemiglandular condition.

More than half a century has passed since Carleton’s (1973) work. With one exception, research on stomach morphology in sigmodontines has progressed through sporadic and limited contributions (Table 2). Most of these consist of brief descriptions—typically based on single specimens—embedded within small-scale taxonomic revisions (*e.g*., Bezerra et al., 2007; Brito et al., 2022). Since Carleton (1980), stomach characters have occasionally been incorporated into cladistic analyses (*e.g*., Voss, 1988; Weksler, 2006; Salazar-Bravo et al., 2023), but detailed anatomical studies remain scarce.

**Table 2:** Gastric morphology in Sigmodontinae based on published literature.

Tribe/subtribe/Clade	Genera	Reference
Abrotrichini	*Abrothrix*	Dorst (1972), Carleton (1973)*, Teta et al. (2011)
	*Chelemys*	
	*Geoxus*	Carleton (1973)*, Mann (1978), D’Elía et al. (2006b)
	*Notiomys*	Pardiñas, Udrizar Sauthier & Teta (2008)
	*Paynomys*	
Akodontina	*Akodon*	Vorontsov (1967), Maggese (1969), Dorst (1972), Carleton (1973)*, Rouaux et al. (2003), Finotti, Moraes Santos & Cerqueira (2012), Pardiñas et al. (2020)
	*Castoria*	Pardiñas et al. (2016b, 2020)
	*Deltamys*	Teta, Cueto & Suárez (2007), Pardiñas et al. (2020)
	*Microxus*	Carleton (1973)*, Pardiñas et al. (2020)
	*Necromys*	Dorst (1972), Carleton (1973)*, Pardiñas et al. (2020)
	*Podoxymys*	Carleton (1973)
	*Thalpomys*	Pardiñas et al. (2020)
	*Thaptomys*	Pardiñas et al. (2020)
Oxymycterina	*Juscelinomys*	Emmons & Patton (2012)
	*Oxymycterus*	Tullberg (1899), Echave Llanos & Vilchez (1964), Vorontsov (1967), Carleton (1973), Pardiñas et al. (2020), Bezerra et al. (2025)
Scapteromyina	*Bibimys*	Gonçalves et al. (2005), Pardiñas, Galliari & Voglino (2017), Pardiñas et al. (2020)
	*Blarinomys*	Geise et al. (2008), Pardiñas et al. (2020)
	*Brucepattersonius*	Hershkovitz (1998), Pardiñas et al. (2020)
	*Gyldenstolpia*	
	*Kunsia*	Bezerra et al. (2007)
	*Lenoxus*	Pardiñas et al. (2020)
	*Scapteromys*	Carleton (1973), Pardiñas et al. (2020)
Andinomyini	*Andinomys*	Salazar-Bravo et al. (2016)
	*Punomys*	Salazar-Bravo et al. (2016)
Euneomyini	*Euneomys*	Carleton (1973)
	*Irenomys*	Carleton (1973)*
	*Neotomys*	Carleton (1973)*
Anotomyina	*Anotomys*	Voss (1988)*
Ichthyomyina	*Chibchanomys*	Voss (1988)*
	*Daptomys*	Carleton (1973)
	*Ichthyomys*	Voss (1988)*
	*Incanomys*	Zeballos et al. (2025)
	*Neusticomys*	Carleton (1973)
	*Rheomys*	Carleton (1973)
Neomicroxini	*Neomicroxus*	Pardiñas et al. (2021)
Clade A	*Scolomys*	
Oryzomyini	*Zygodontomys*	Carleton (1973)
Clade B	*Casiomys*	Carleton (1973)*
Oryzomyini	*Euryoryzomys*	
	*Handleyomys*	Voss, Gómez-Laverde & Pacheco, 2002
	*Hylaeamys*	Carleton (1973)*
	*Mindomys*	Brito et al. (2022)
	*Nephelomys*	Carleton (1973)*
	*Oecomys*	Carleton (1973)*, Pardiñas et al. (2016a)
	*Pattonimus*	Brito et al. (2020)
	*Transandinomys*	Carleton (1973)*
Clade C	*Microryzomys*	Carleton & Musser (1989), Noblecilla Huiman (2008)
Oryzomyini	*Neacomys*	Carleton (1973)*, Tinoco et al. (2023)
	*Oligoryzomys*	Carleton (1973)*, Mann (1978), Carleton & Musser (1989)
	*Oreoryzomys*	Brito et al. (2025)
Clade D	*Aegialomys*	Patton & Hafner (1983), Guabloche, Arana & Ramírez (2002)
	*Amphinectomys*	
	*Cerradomys*	Percequillo, Hingst-Zaher & Bonvicino (2008)*
	*Drymoreomys*	Percequillo, Weksler & Costa (2011)*
	*Eremoryzomys*	
	*Holochilus*	Carleton (1973)
	*Lundomys*	Voss & Carleton (1993)
	*Melanomys*	Carleton (1973)*
	*Microakodontomys*	
	*Nectomys*	Vorontsov (1962, 1967), Carleton (1973)
	*Nesoryzomys*	Patton & Hafner (1983)
	*Oryzomys*	Vorontsov (1967), Carleton (1973)*, Carleton & Musser (1989)
	*Pseudoryzomys*	Voss & Myers (1991)*
	*Sigmodontomys*	
	*Sooretamys*	Chiquito, D’Elía & Percequillo (2014)*
	*Tanyuromys*	
Calomyina	*Calassomys*	Pardiñas et al. (2014)
	*Calomys*	Maggese (1969), Dorst (1972), Carleton (1973), Ellis et al. (1994), Rouaux et al. (2003)
Phyllotina	*Auliscomys*	Dorst (1972)
	*Andalgalomys*	
	*Eligmodontia*	Cuvier (1837), Carleton (1973)*
	*Galenomys*	
	*Graomys*	Carleton (1973)
	*Loxodontomys*	
	*Phyllotis*	Dorst (1972), Carleton (1973)*
	*Salinomys*	
	*Tapecomys*	
Reithrodontini	*Reithrodon*	Carleton (1973)*
Rhagomyini	*Rhagomys*	Luna & Patterson (2003)
Sigmodontini	*Sigmodon*	Blank (1950), Vorontsov (1962, 1967), Carleton (1973)*
Thomasomyini	*Aepeomys*	Voss, Gómez-Laverde & Pacheco (2002), Pacheco (2003)
	*Chilomys*	Carleton (1973)
	*Rhipidomys*	Carleton (1973)*
	*Thomasomys*	Carleton (1973), Noblecilla Huiman (2008), Pacheco (2003), Pacheco & Ruelas (2023)
Wiedomyini	*Juliomys*	
	*Phaenomys*	
	*Wiedomys*	
	*Wilfredomys*	
Incertae sedis	*Abrawayaomys*	
	*Chinchillula*	Dorst (1972)
	*Delomys*	

**Note:**

References marked with an asterisk (*) indicate mentions lacking detailed descriptions and/or illustrations.

The only comprehensive study focused exclusively on stomach morphology is that of Pardiñas et al. (2020), centered on the tribe Akodontini, the second most diverse group within Sigmodontinae. Building on Carleton (1973), the authors examined 36 species, covering 15 genera when combined with literature data. They proposed a refined classification of stomach types: Type A, the classic unilocular-hemiglandular stomach; Type B, a unilocular-discoglandular stomach; and Type C, a unilocular-diverticular stomach, in which the glandular epithelium is confined to a pouch or diverticulum, following Langer (2017). Pardiñas et al. (2020) interpreted the coexistence of these stomach types within Akodontini as evidence that the tribe may be overinflated and could represent at least three distinct tribes or subtribes.

Although Carleton’s (1973) study remains foundational, with its classification and terminology still widely adopted and only slightly modified (Langer, 2017), it also represents the last comprehensive, subfamily-wide analysis of stomach morphology in Sigmodontinae. Over the past five decades, no major re-evaluation of this anatomical system has been conducted at a comparable scale, despite advances in taxonomy, phylogenetics, and analytical methods. As a result, many aspects of stomach morphological diversity, variation within tribes, and evolutionary trends remain insufficiently explored.

## Results

### A refined classification

Having directly examined approximately 80% of the extant generic diversity of the subfamily—rising to 91% when all available literature evidence is included (Table 3)—this morphological overview supports several refinements to Carleton’s (1973) seminal classification of stomach morphology. A trait originally emphasized by that author—the penetration of the dextral gastroesophageal angle, or incisura angularis—is here reinforced as a key attribute characterizing the overall anatomical design of this group. With a single partial exception (in the genus *Phaenomys*; see below), the incisura angularis is shallow and, in the absence of other deep infoldings, the gastric lumen appears morphologically undivided and thus unilocular (*sensu* Carleton, 1973). Therefore, as established by Carleton (1973), all known sigmodontines possess unilocular stomachs.

**Table 3:** Diversity of gastric morphology in Sigmodontinae based on a refined generic classification.

Tribe/other	Genera	Classification	Remarks
Abrotrichini	*Abrothrix*	Equiglandular	
	*Chelemys*	–	No fluids available
	*Geoxus*	Equiglandular	
	*Notiomys*	Equiglandular	
	*Paynomys*	Equiglandular	
Akodontina	*Akodon*	Equiglandular	
	*Castoria*	Equiglandular	
	*Deltamys*	Equiglandular	
	*Microxus*	Equiglandular	
	*Necromys*	Equiglandular	*N. obscurus* tends supraglandular
	*Podoxymys*	Equiglandular	
	*Thalpomys*	Equiglandular	
	*Thaptomys*	Equiglandular	
Oxymycterina	*Juscelinomys*	Diverticular	
	*Oxymycterus*	Diverticular	
Scapteromyina	*Bibimys*	Equiglandular	
	*Blarinomys*	Diverticular	
	*Brucepattersonius*	Diverticular	
	*Gyldenstolpia*	–	No fluids available
	*Kunsia*	Diverticular	
	*Lenoxus*	Diverticular	
	*Scapteromys*	Discoglandular	
Andinomyini	*Andinomys*	Equiglandular	
	*Punomys*	Equiglandular	
Euneomyini	*Euneomys*	Supraglandular	
	*Irenomys*	Supraglandular	
	*Neotomys*	Supraglandular	
Anotomyina	*Anotomys*	Subglandular	Fide (Voss, 1988)
Ichthyomyina	*Chibchanomys*	Subglandular	Fide (Voss, 1988)
	*Daptomys*	Subglandular	
	*Ichthyomys*	Subglandular	
	*Incanomys*	Subglandular	Fide (Zeballos et al., 2025)
	*Neusticomys*	Subglandular	
	*Rheomys*	Subglandular	Fide (Carleton, 1973)
Neomicroxini	*Neomicroxus*	Equiglandular	
Clade A	*Scolomys*	Equiglandular	
	*Zygodontomys*	Equiglandular	
Clade B	*Casiomys*	Equiglandular	
	*Euryoryzomys*	Equiglandular	
	*Handleyomys*	Equiglandular	Fide (Voss, Gómez-Laverde & Pacheco, 2002)
	*Hylaeamys*	Equiglandular	
	*Mindomys*	Equiglandular	
	*Nephelomys*	Equiglandular	
	*Oecomys*	Equiglandular	
	*Pattonimus*	Equiglandular	
	*Transandinomys*	Equiglandular	
Clade C	*Microryzomys*	Equiglandular	
	*Neacomys*	Equiglandular	
	*Oligoryzomys*	Equiglandular	
	*Oreoryzomys*	Equiglandular	
Clade D	*Aegialomys*	Equiglandular	
	*Amphinectomys*	–	No fluids available
	*Cerradomys*	Equiglandular	
	*Drymoreomys*	Equiglandular	Fide (Percequillo, Weksler & Costa, 2011)
	*Eremoryzomys*	–	
	*Holochilus*	Supraglandular	
	*Lundomys*	Equiglandular	
	*Melanomys*	Equiglandular	
	*Microakodontomys*	–	No fluids available
	*Nectomys*	Equiglandular	
	*Nesoryzomys*	Equiglandular	Fide (Patton & Hafner, 1983)
	*Oryzomys*	Equiglandular	Fide (Carleton & Musser, 1989)
	*Pseudoryzomys*	Equiglandular	
	*Sigmodontomys*	Equiglandular	
	*Sooretamys*	Equiglandular	
	*Tanyuromys*	Equiglandular	
Calomyina	*Calassomys*	Equiglandular	
	*Calomys*	Equiglandular	
Phyllotina	*Auliscomys*	Equiglandular	
	*Andalgalomys*	–	
	*Eligmodontia*	Equiglandular	
	*Galenomys*	–	No fluids available
	*Graomys*	Equiglandular	
	*Loxodontomys*	Equiglandular	
	*Phyllotis*	Equiglandular	
	*Salinomys*	–	
	*Tapecomys*	Equiglandular	
Reithrodontini	*Reithrodon*	Supraglandular	
Rhagomyini	*Rhagomys*	Equiglandular	
Sigmodontini	*Sigmodon*	Equiglandular	
Thomasomyini	*Aepeomys*	Diverticular	
	*Chilomys*	Equiglandular	
	*Rhipidomys*	Equiglandular	
	*Thomasomys*	Equiglandular	Several species subglandular
Wiedomyini	*Juliomys*	Equiglandular	
	*Phaenomys*	Subglandular	
	*Wiedomys*	Equiglandular	
	*Wilfredomys*	Equiglandular	
Incertae sedis	*Abrawayaomys*	Equiglandular	
	*Chinchillula*	Equiglandular	
	*Delomys*	Equiglandular	
Total	91	83	
%genus/total		91.2	

However, the original classification, restricted to two basic configurations describing the internal distribution of glandular and cornified epithelia, requires refinement to better reflect the currently documented diversity. Carleton (1973) defined two principal anatomical designs: hemiglandular and discoglandular. In the former, the glandular epithelium is confined more or less to the antrum, with the *corpus* lined by cornified epithelium. In the latter, the glandular epithelium is limited to a disc-shaped area along the greater curvature, while the cornified epithelium covers the remainder of the internal surface of the organ.

Carleton (1973) also mentioned the “pouched condition” to describe cases in which the glandular epithelium is restricted to a diverticulum or pouch. Langer (2017: 202, fig. 4.82) recognized the need to adjust Carleton’s (1973) scheme and introduced the subhemiglandular category to accommodate examples in which the glandular epithelium occupies a smaller portion of the antrum. In addition, that author formalized the “pouched condition,” coining the term diverticular. Within this framework, the terminology adopted by Pardiñas et al. (2020)—who distinguished three stomach “types” identified by letters—appears suboptimal, because the use of lettered categories reduces the descriptive value inherent in morphological terms such as diverticular.

**Figure 3:**
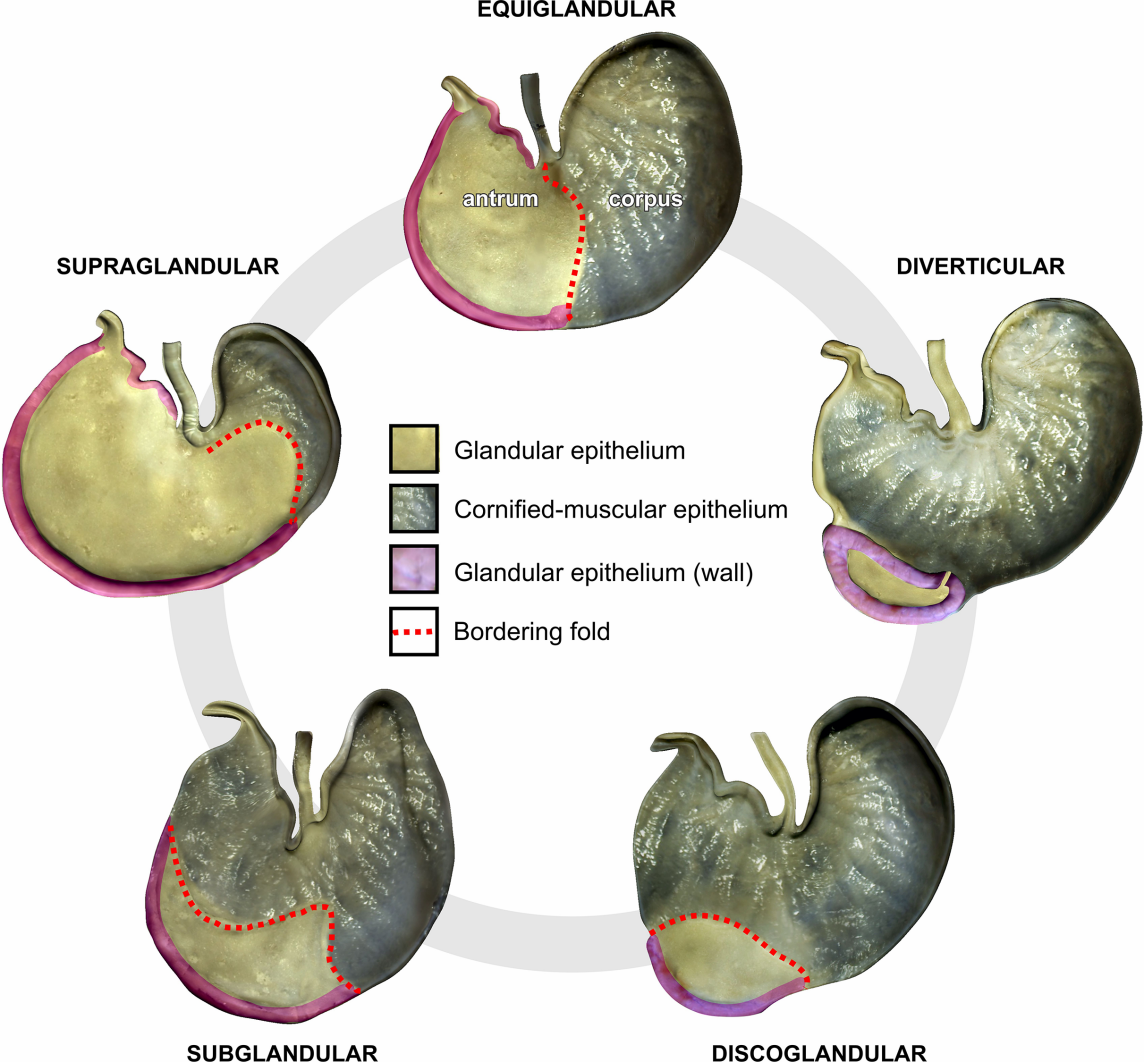
Refined classification of stomach types in Sigmodontinae rodents. Silhouettes illustrate the main internal configurations based on the distribution of two epithelial types. The order of presentation does not imply evolutionary relationships. Artwork by D. Voglino.

The classification proposed here returns to the terminology introduced by Carleton (1973) and retained and refined by Langer (2017), owing to its descriptive clarity. Because all sigmodontine stomachs are unilocular, this classification emphasizes the internal distribution of the glandular epithelium (Fig. 3).

The most common anatomical design is termed equiglandular; the prefix *equi-* denotes “equal” or “equally” and highlights the roughly even distribution of glandular and cornified epithelia lining the internal surface of the organ. This category corresponds in part to the hemiglandular type *sensu* Carleton (1973). The equiglandular group encompasses minor variations in the extent of the glandular epithelium, whether it is strictly confined to the antrum or extends slightly beyond the level of the esophageal opening or the incisura angularis to the right or left.

All other recognized anatomical designs are less frequent but represent well-defined morphological patterns.

Supraglandular stomachs are characterized by glandular epithelium extending broadly over the antrum and a large portion of the *corpus*, whereas cornified epithelium is mostly confined to the right wall of the *corpus* and to the fundus of the fornix ventricularis. The contrasting condition, subglandular (equivalent to subhemiglandular *sensu* Langer, 2017), involves glandular epithelium covering a smaller portion of the antral surface, usually because the pars pylorica—either totally or partially—is lined by cornified epithelium. Discoglandular stomachs (*sensu* Carleton, 1973) display glandular epithelium lining the fundus along the greater curvature, extending over parts of both the antrum and *corpus*. Diverticular stomachs (*sensu* Langer, 2017; also “pouched” *sensu* Carleton, 1973) are distinguished by the confinement of glandular epithelium to a diverticulum or pouch located external to the main gastric lumen.

The bordering fold, defined by Carleton (1973) as the boundary between glandular and cornified epithelia, is not directly involved in the classification described above. However, as one of the most conspicuous anatomical features of the stomach, it warrants further consideration.

Most previous observations have focused on the form of the bordering fold in its medial portion, between the incisura angularis and the corresponding point along the greater curvature (*e.g*., Carleton, 1973; Pardiñas et al., 2020). This approach is informative primarily for assessing the extent of glandular epithelium within the *corpus*.

**Figure 4:**
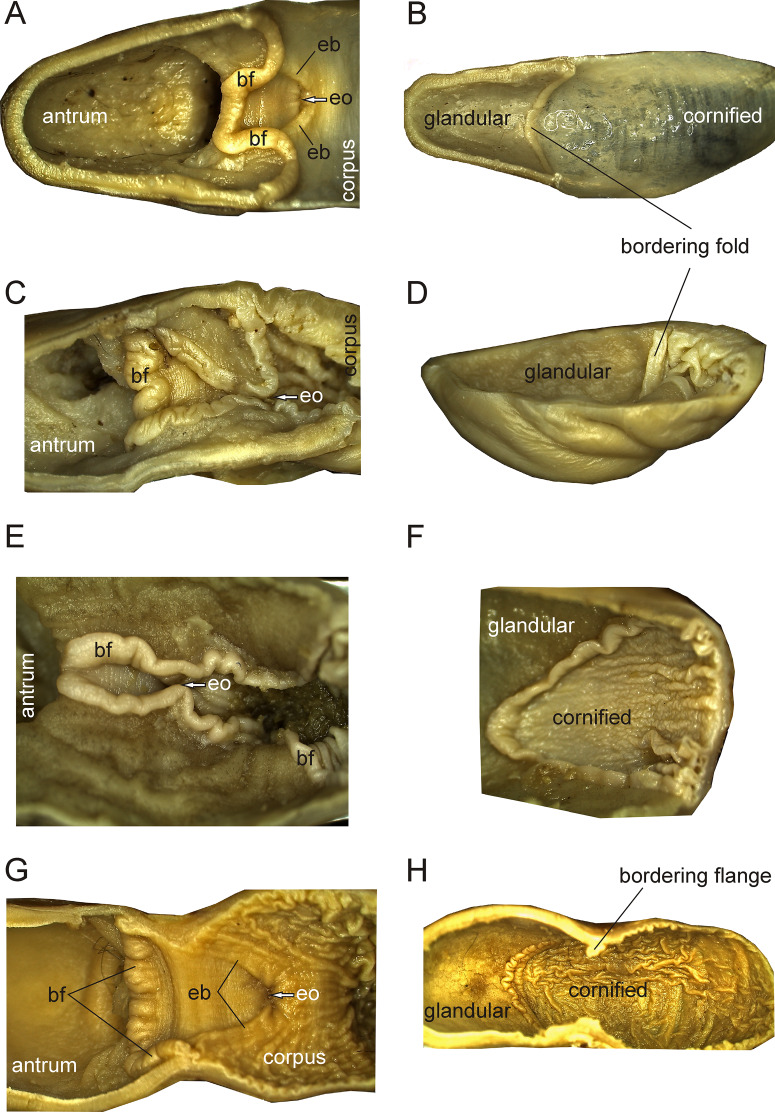
Diversity of internal morphologies surrounding the esophageal opening in sigmodontine stomachs. Squared type in *Abrothrix hirta* (A, B; CNP 6348); amphoral type in *Irenomys tarsalis* (C, D; CNP-D 216); irregular type in *Holochilus chacarius* (E, F; CNP 6645); triangular type in *Loxodontomys micropus* (G, H; CNP 5338). Left column: views centered on the esophageal opening. Right column: views showing the distribution of cornified and glandular epithelia along the greater curvature. Abbreviations: eo, esophageal opening; bf, bordering fold; eb, esophageal bordering fold. All figures are shown at approximately the same scale. Photographs by U. Pardiñas.

In the present study, transverse cuts applied to selected specimens allowed examination of the bordering fold around the esophageal opening. At least four main anatomical configurations were identified, involving the spatial relationships among the bordering fold, the plica angularis, the esophageal opening, and the boundary between the esophageal and cornified epithelia (Fig. 4).

One configuration, termed squared, consists of a bordering fold encircling the esophageal opening, with parallel “arms” connected by a transverse segment that conceals (or nearly conceals) the plica angularis. In addition, the junction between the esophageal and stomach cornified epithelia is expressed as two short margins (*i.e*., esophageal bordering fold). Another configuration, termed amphoral, displays elongated “arms” forming a triangular outline, with the right vertex occupied by the esophageal opening. A third configuration, irregular, is closely related to the latter and reflects variation in the length of the bordering fold “arms,” depending on the extent of glandular epithelium within the *corpus*. Finally, a distinctive configuration, termed triangular, is characterized by the dominance of the margins separating the esophageal and stomach cornified epithelia in the overall morphology, associated with very short “arms” and a markedly corrugated transverse connection passing over the plica angularis.

### Intratribal comparative anatomy

In this section, a comparative anatomical description of the gross internal morphology of the stomach is provided focusing on the intratribal variation. The presentation is alphabetically ordered (by tribes), and the characterizations employ the classification terminology advanced in the previous section.

**Figure 5:**
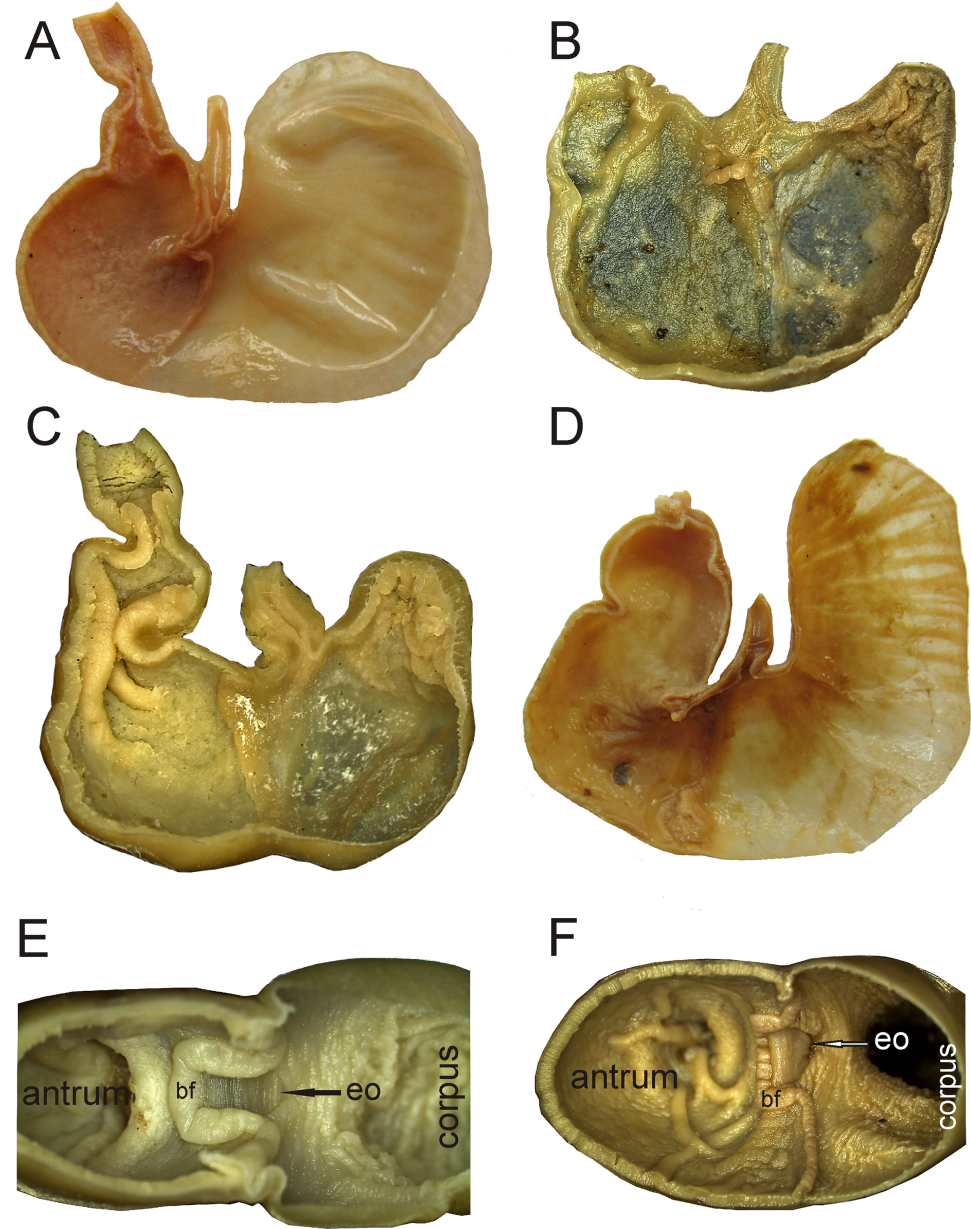
Internal stomach views (ventral and anterior aspects) in members of Abrotrichini. *Abrothrix olivacea* (A; CNP 6663), *Geoxus valdivianus* (B; CNP 6280), *Notiomys edwardsii* (C; CNP 6286), *Paynomys macronyx* (D; CNP 442), *Abrothrix gossei* (E; CNP 7089), and *P. macronyx* (F; CNP 3889). Abbreviations: bf, bordering fold; eo, esophageal opening. All figures are shown at approximately the same scale. Photographs by U. Pardiñas.

Abrotrichini (studied: two subtribes, four genera, seven species; Fig. 5). All examined stomachs are equiglandular, exhibiting a roughly equal internal distribution of glandular and cornified epithelia in the antrum and *corpus*, respectively. The contrast between these epithelial surfaces is not sharp, and the bordering fold runs straight and is not particularly conspicuous. *Geoxus* and *Notiomys* show a noticeable concentration of corrugated cornified epithelium restricted to the fornix ventricularis, associated with a more sacciform overall morphology. Members of the Abrotrichina (*i.e*., *Abrothrix*; Fig. 5A) show a deeper incisura angularis than those of the Notiomyina (except *Paynomys*; Fig. 5D), as well as a more developed fornix ventricularis. The pars pylorica is more distinctly defined in Notiomyina than in Abrotrichina. Around the esophageal opening, the bordering fold forms a squared outline that continues into the plica angularis (Figs. 5E, 5F).

Remarks. *Chelemys*, the only abrotrichine lacking available stomachs in collections, probably conforms to the equiglandular condition, given its close morphological resemblance to other members of the Notiomyina (Teta et al., 2017). Minor variation in the extension of the glandular epithelium into the *corpus* has been previously noted (*e.g*., Pardiñas, Udrizar Sauthier & Teta, 2008; Teta et al., 2017: fig. 16). With a larger sample examined, this difference appears to be an artefactual effect resulting from the inspection of incompletely expanded stomachs.

**Figure 6:**
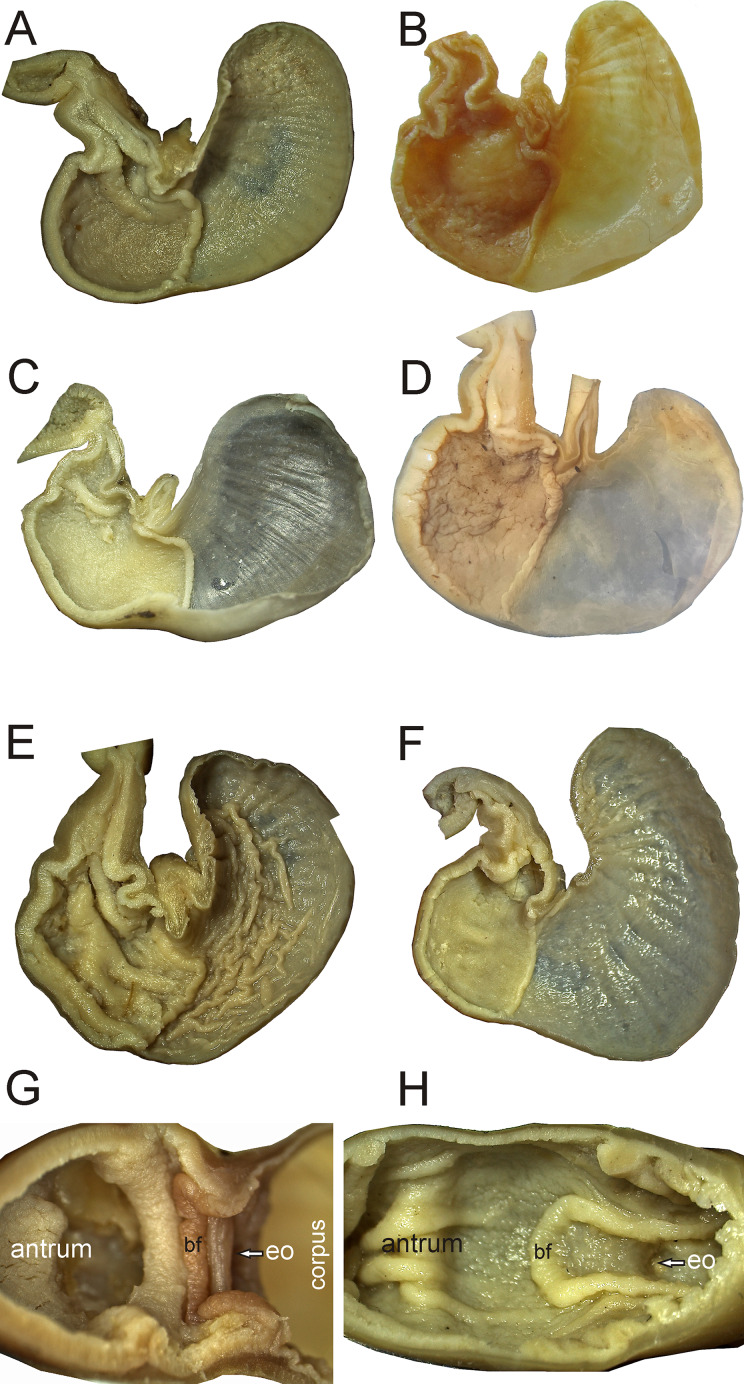
Internal stomach views of Akodontini (Akodontina). Ventral views (A–F): *Akodon oenos* (A; CNP-D 309), *Castoria angustidens* (B; CNP 449), *Deltamys kempi* (C; CNP 6713), *Microxus mimus* (D; CBF 9900), *Necromys lasiurus temchuki* (E; CNP 6576), and *Thaptomys nigrita* (F; CNP 7814). Anterior views: *Akodon azarae* (G; CNP 3115) and *Necromys obscurus* (H; CNP 8576). Abbreviations: bf, bordering fold; eo, esophageal opening. All figures are shown at approximately the same scale. Photographs by U. Pardiñas.

**Figure 7:**
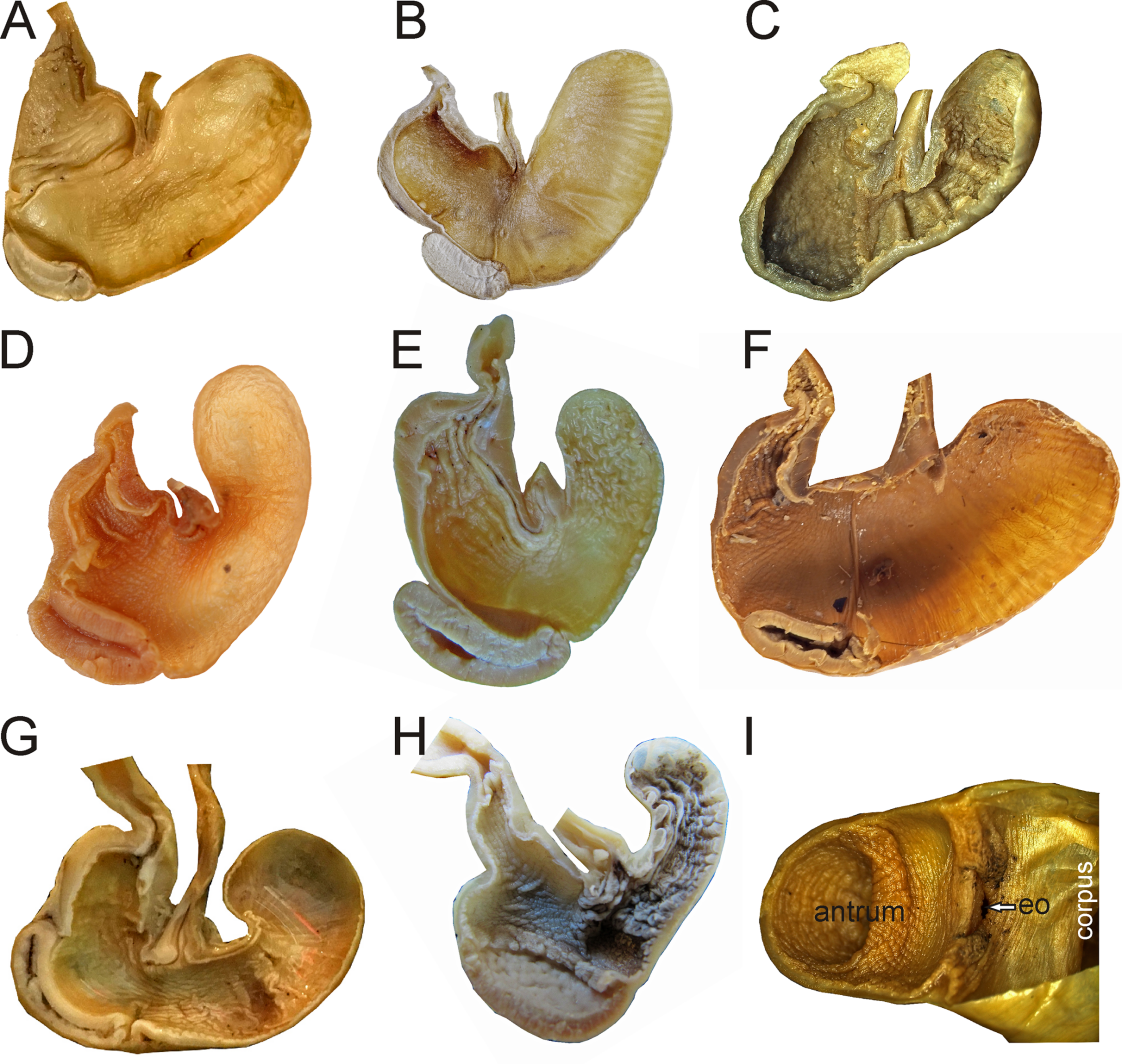
Internal stomach views of Akodontini (Oxymycterina and Scapteromyina). Ventral views (A–H) and an anterior view (I): Juscelinomys cf. *J. huanchacae* (A; CBF 12045), *Oxymycterus inca* (B; CBF 9355), *Bibimys labiosus* (C; CNP 8652), *Blarinomys breviceps* (D; ARA 306), *Brucepattersonius iheringi* (E; CNP 1932), *Kunsia tomentosus* (F; MN 62569), *Lenoxus apicalis* (G; CBF 10124), and *Scapteromys aquaticus* (H; CNP 6404; I; CNP 6666). Abbreviations: bf, bordering fold; eo, esophageal opening. All figures are shown at approximately the same scale. Photographs by U. Pardiñas.

Akodontini (studied: three subtribes, 15 genera, 34 species; Figs. 6, 7). This tribe exhibits at least three primary stomach configurations and a notable diversity of minor variations. The Akodontina are characterized by equiglandular stomachs, although one species of *Necromys* (*N. obscurus*) tends toward a supraglandular condition (Fig. S2). The Oxymycterina display a diverticular configuration, whereas the Scapteromyina include three types: *Bibimys* is equiglandular, *Scapteromys* is discoglandular, and the remaining members are diverticular (Fig. 7). The trenchant differences among these three subtribes extend beyond this basic classification. The Akodontina possess a deeper incisura angularis—although it never penetrates extensively—compared with the Oxymycterina and Scapteromyina. In addition, Akodontina show a larger *corpus*, typically smooth internally (except in *Necromys*; Fig. 6E), with a well-developed fornix ventricularis that often forms a prominent, anteriorly projecting, convoluted space (*e.g*., Figs. 6A, 6F). In contrast, the Oxymycterina and Scapteromyina possess a more developed and strongly muscular antrum, with a sharply contrasting cornified lining between the antrum and *corpus*. This contrast produces a false bordering fold, expressed as a ridge dividing the main cavity of the organ (*e.g*., Figs. 7F, 7H). Furthermore, the corrugated surface of the antrum and *corpus* in Oxymycterina and Scapteromyina (except *Bibimys*; Fig. 7C) is much more pronounced than in Akodontina, as is the greater development of the pars pylorica. The pouch in which the glandular epithelium is recessed in diverticular forms is generally similar among members of Oxymycterina and Scapteromyina, though it appears to be more developed in the latter (Fig. 7). This pouch connects to the main lumen of the stomach through a minute orifice that consistently opens into the *corpus*. In Akodontina, the configuration of the bordering fold around the esophageal opening resembles that described for the Abrotrichini (Figs. 6G, 6H).

Remarks. *Scapteromys* remains the only known discoglandular example among sigmodontines to date, as first identified by Carleton (1973). Although distinct within its subtribe, the glandular lining in the equiglandular condition of *Bibimys* is clearly less extensive than in equiglandular Akodontina (Pardiñas, Galliari & Voglino, 2017; Pardiñas et al., 2020). *Podoxymys*, not examined here, also exhibits a limited glandular covering of the antrum, consistent with *Bibimys* (cf. Carleton, 1973: fig. 5A).

**Figure 8:**
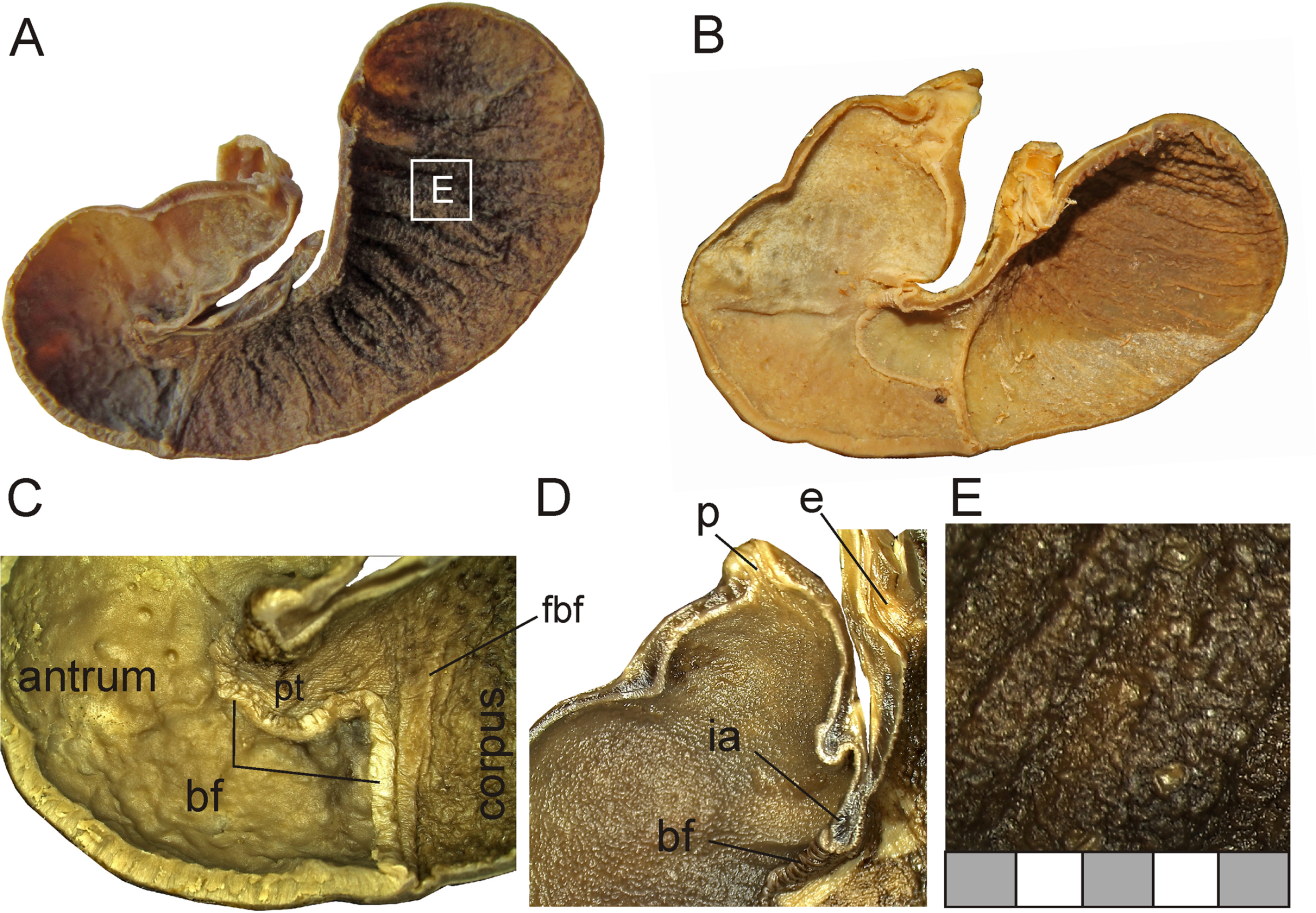
Internal stomach views of Andinomyini. Ventral views: *Andinomys edax* (A, C; CNP 5491) and Punomys cf. *P. kofordi* (B; MUSA 4692). (D) Detail of the plica angularis. (E) Magnified view of the internal surface of the *corpus*. Abbreviations: bf, bordering fold; e, esophagus; eo, esophageal opening; fbf, false bordering fold; ia, incisura angularis; p, pylorus; pt, cornified epithelium protrusion. Scale divisions in E are in millimeters. All figures are shown at approximately the same scale. Photographs by U. Pardiñas (A, C–E) and H. Zeballos (B).

Andinomyini (studied: two genera, two species; Fig. 8). Both genera included in this tribe share an equiglandular configuration. They also share a diagnostic trait consisting of an identifiable protrusion of the cornified epithelium toward the right side (Fig. 8C). In this anterior region, the bordering fold circumscribes an area that contacts a deep and distant plica angularis (Fig. 8D). Reinforcing this unique morphology, a false bordering fold—produced by the differing surface textures of the cornified epithelia between the *corpus* and the aforementioned anterior portion—extends toward the esophageal opening (*e.g*., Fig. 8B). The *corpus* itself is greatly developed, with a globose fornix ventricularis and an internal surface characterized by marked, parallel transverse ridges (accordion-like; Fig. 8E). Andinomyine stomachs are further distinguished by the noticeable difference in the penetration of the incisura cardialis relative to the incisura angularis, the latter being associated with a well-developed pars pylorica that imparts the organ a distinctly bi-chambered appearance (Figs. 8A, 8B).

Remarks. Salazar-Bravo et al. (2016) erroneously interpreted this anterior distinctive protrusion as being covered by glandular epithelium.

**Figure 9:**
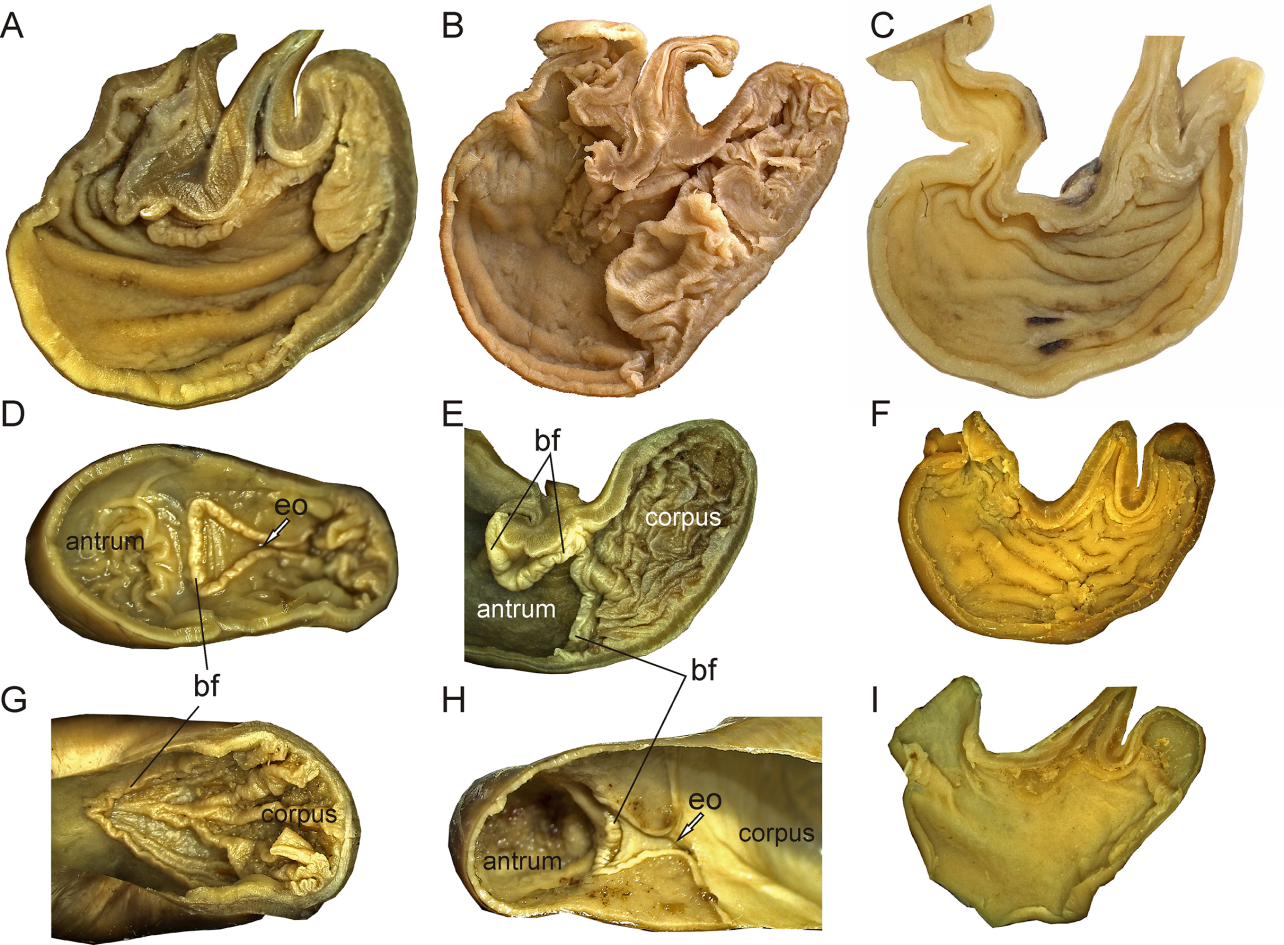
Internal stomach views of Euneomyini. Ventral (A–C, E–F, I), anterior (D, H), and posterior (G) views: *Euneomys chinchilloides* (A, D, G; CNP 7190, CNP 7188, CNP 7188), *Irenomys tarsalis* (B, E, H; CNP 5295, CNP-D 214, CNP 5424), and *Neotomys ebriosus* (C, F, I; RCC 479, CNP 3639, CNP 3639). Abbreviations: bf, bordering fold; eo, esophageal opening. All figures are shown at approximately the same scale. Photographs by U. Pardiñas (A, B, D–I) and R. Cairampoma (C).

Euneomyini (studied: three genera, three species; Fig. 9). All three genera included in this tribe share a supraglandular stomach configuration. The glandular lining extends extensively into the *corpus* in *Neotomys* (Figs. 9C, 9F, 9I), restricting the cornified epithelium to a small portion of the fornix ventricularis, whereas in *Irenomys* the intrusion is less pronounced (Figs. 9B, 9E). The cornified surface is sharply plicate, with some lamellar folds projecting prominently to the right (*e.g*., in *Euneomys*; Fig. 9A). The incisura cardialis is positioned markedly anterior to the incisura angularis. The anterior portion of the bordering fold around the esophageal opening forms a characteristic amphoral outline (Figs. 9D, 9H). Overall, the stomachs in this tribe are relatively small compared with body size.

Remarks. Carleton (1973: 25, 28) first noted the extensive development of the glandular epithelium in *Euneomys* and *Neotomys* (see also Pardiñas, Teta & Salazar-Bravo, 2015).

**Figure 10:**
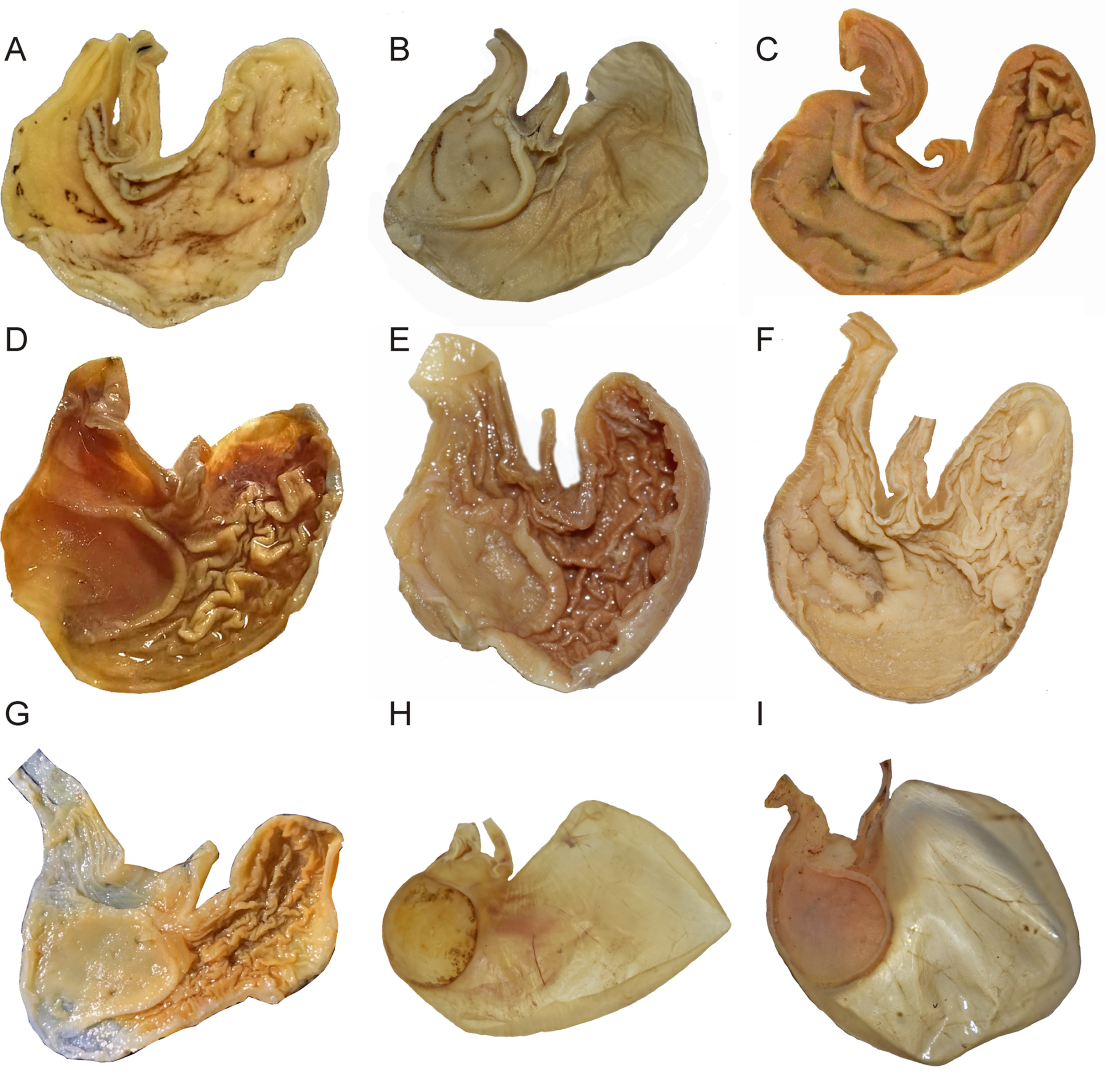
Internal stomach views of Ichthyomyini. Ventral views: *Daptomys peruviensis* (A, B; MECN 8072, MECN 7175), *Ichthyomys hydrobates* (C; QCAZ 818; D; CVULA 7821), *Ichthyomys pinei* (E; MECN 5613), *Ichthyomys orientalis* (F; MECN 6370), *Ichthyomys stolzmanni* (G; MUSA 18920), *Ichthyomys tweedii* (H; MECN 5772), and *Neusticomys vossi* (I; MECN 7666). All figures are shown at approximately the same scale. Photographs by J. Brito (A, B, E, F, H, I), C. Cañón (C), B. Rivas (D), and H. Zeballos (G).

Ichthyomyini (examined: one subtribe, three genera, eight species; Fig. 10). The examined stomachs exhibit a subglandular configuration. In *Ichthyomys* (*e.g*., Figs. 10D, 10F), the glandular epithelium occupies a smaller portion of the antral surface compared with *Daptomys* (Figs. 10A, 10B) and *Neusticomys* (Fig. 10I). These glandular areas are concentrated on the right side of the organ, leaving the greater curvature lined by cornified epithelium. Overall, the incisura angularis (very shallow) and the incisura cardialis lie at approximately the same level. The *corpus* is lined internally with cornified epithelium of a reticulate appearance, particularly in *Ichthyomys* (Figs. 10C–10G). The bordering fold presents a half-moon shape, and the pars pylorica is inconspicuous.

Remarks. Although the present assessment covers fewer than half of the known genera (<50% of generic diversity), the overall stomach morphology of this tribe can be complemented with published descriptions of *Rheomys* (Carleton, 1973), *Anotomys* and *Chibchanomys* (Voss, 1988), and the recently described *Incanomys* (Zeballos et al., 2025). The subglandular condition appears to be shared by all known members of the tribe.

**Figure 11:**
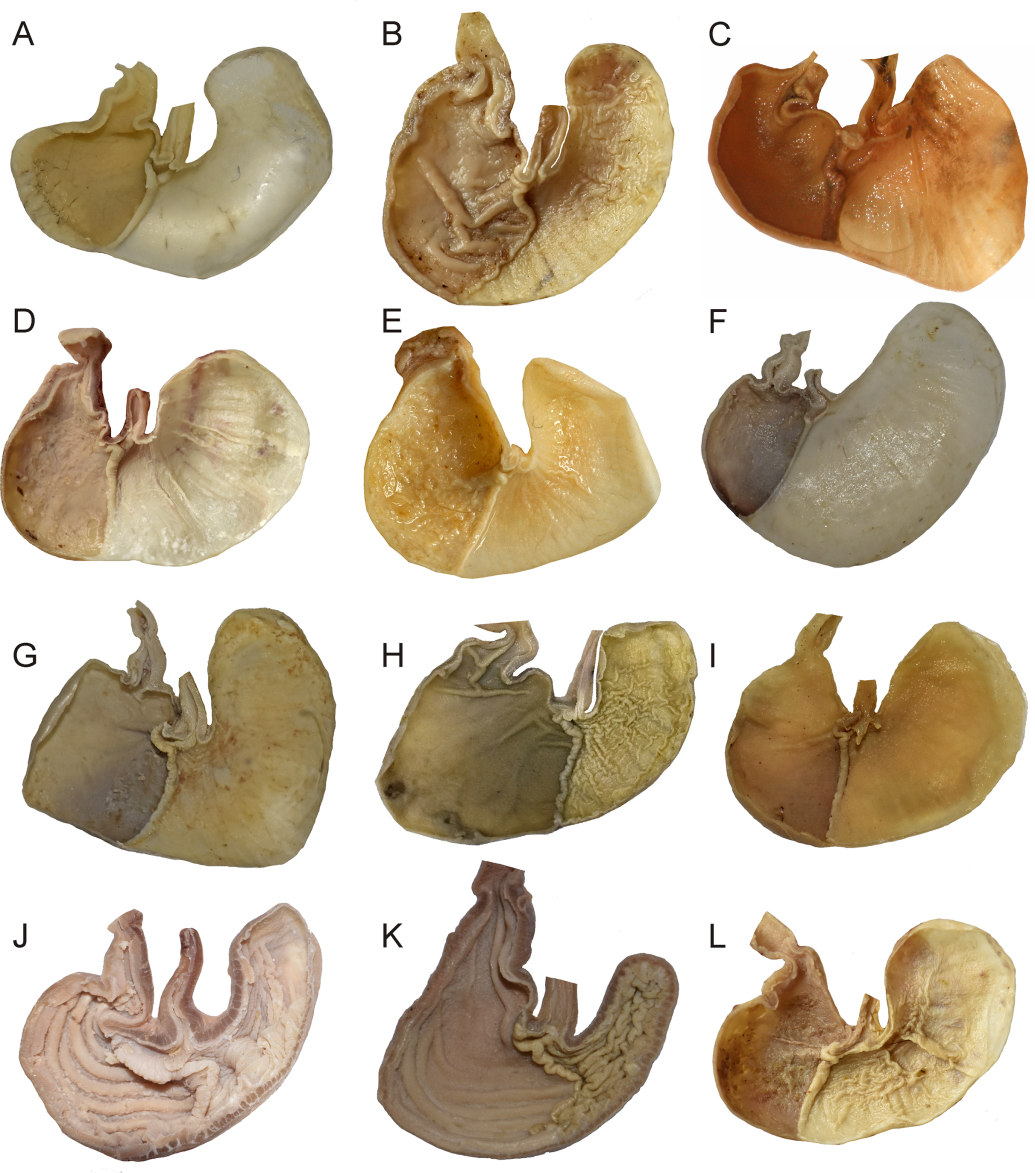
Internal stomach views of Neomicroxini and Oryzomyini (clades A and B). Ventral views: *Neomicroxus latebricola* (A; MECN 6927), *Scolomys melanops* (B; MECN 5777), *Zygodontomys brevicauda* (C; CVULA 194), Casiomys alfaroi (D; MECN 5753), *Casiomys rostratus* (E; CMZ 91), *Euryoryzomys macconnelli* (F; MECN 3164), *Hylaeamys tatei* (G; MECN 7145), *Mindomys hammondi* (H; MECN 6228), *Nephelomys moerex* (I; MECN 4936), *Oecomys bicolor* (J; MECN 6374), *Pattonimus ecominga* (K; MECN 6336), and *Transandinomys talamancae* (L; MECN 5768). All figures are shown at approximately the same scale. Photographs by J. Brito (A, B, D, F–L), B. Rivas (C), and L. León-Panigua (E).

Neomicroxini (examined: one genus, one species; Fig. 11A). The single representative of this tribe exhibits an equiglandular stomach, characterized by an approximately equal distribution of glandular and cornified epithelia in the antrum and *corpus*, respectively. Both main incisurae are very shallow and positioned at the same level. The bordering fold forms a broad cord that deflects slightly to the right near the esophageal opening.

**Figure 12:**
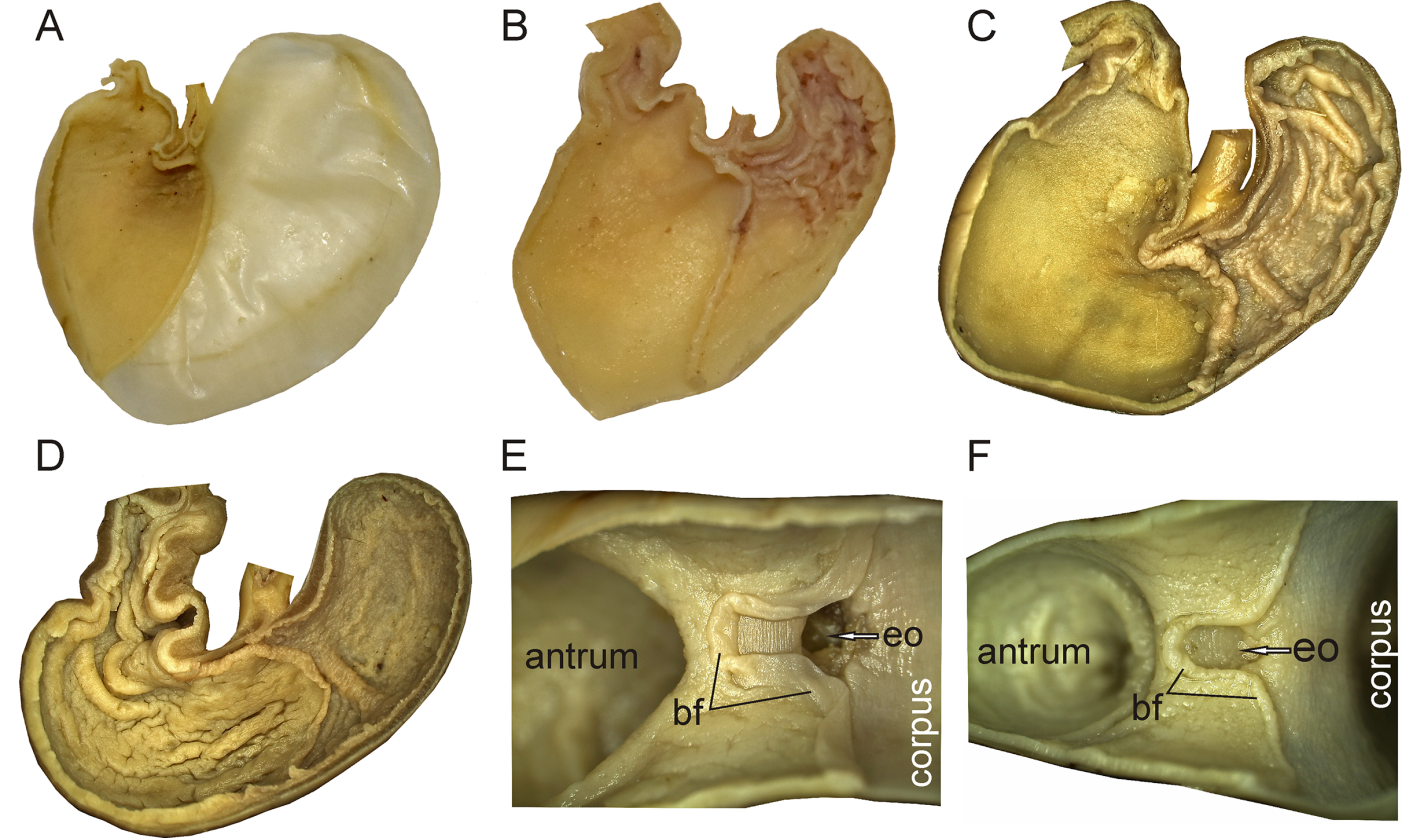
Internal stomach views of Oryzomyini (clade C). Ventral views (A–D) and anterior views (E, F): *Microryzomys minutus* (A; MECN 7687), *Neacomys rosalindae* (B; MECN 4159), Oligoryzomys brendae (C; CNP 7744), *Oligoryzomys longicaudatus* (D; CNP 7022), O. brendae (E; CNP 6646), and *Oligoryzomys flavescens* (F; CNP 7521). Abbreviations: bf, bordering fold; eo, esophageal opening. All figures are shown at approximately the same scale. Photographs by J. Brito (A, B) and U. Pardiñas (C–F).

**Figure 13:**
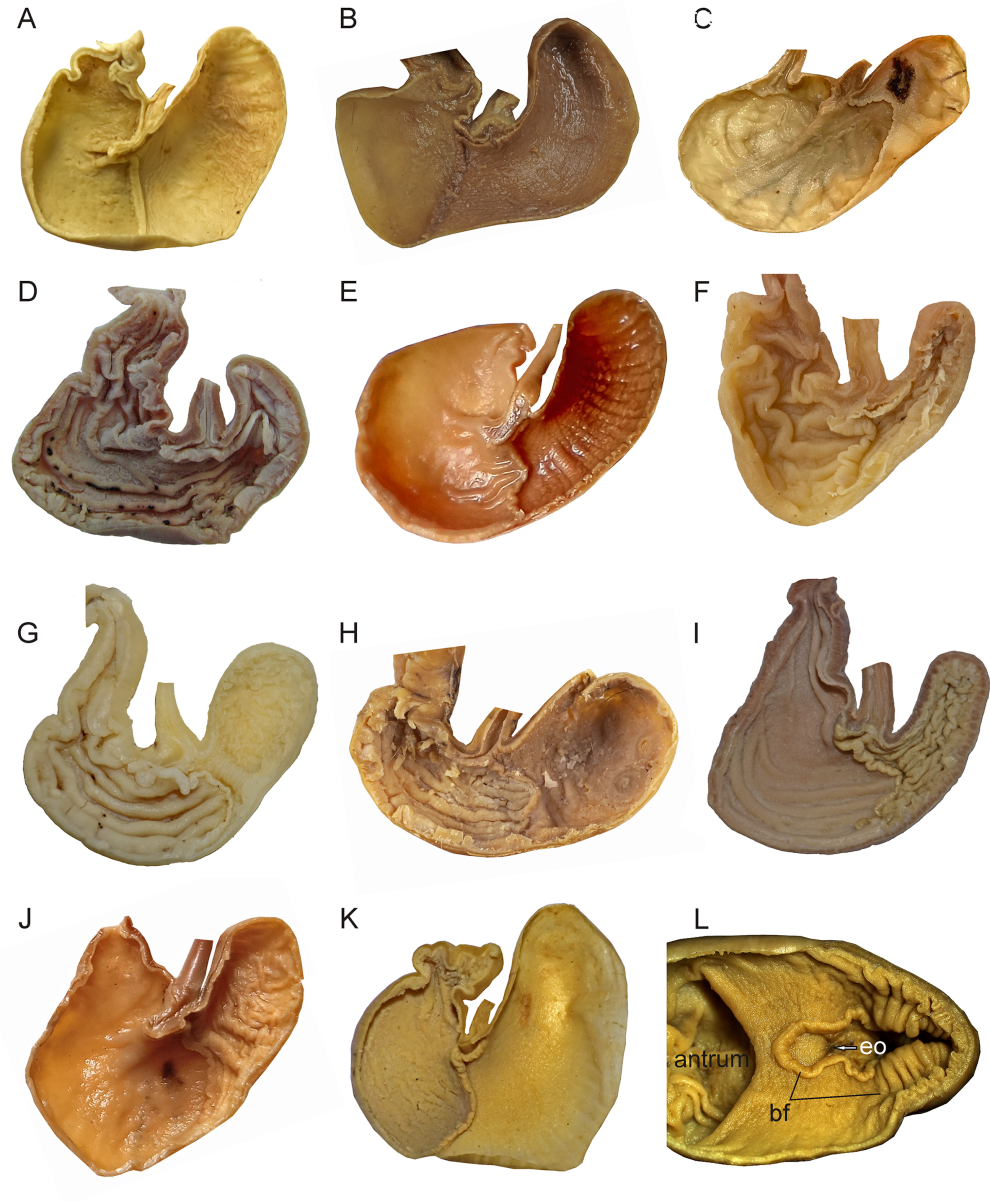
Internal stomach views of Oryzomyini (clade D). Ventral views (A–K) and an anterior view (L): *Aegialomys xanthaeolus* (A; MECN 8313), *Cerradomys vivoi* (B; MN 34433), *Holochilus nanus* (C; CBF 12062), *Holochilus brasiliensis* (D; CNP 8616; L; CNP 5314), *Lundomys molitor* (E; UFSC 5005), *Melanomys caliginosus* (F; MECN 5968), *Nectomys apicalis* (G; MECN 5864), *Pseudoryzomys simplex* (H; MN 66269), *Sigmodontomys alfari* (I; MECN 6022), *Sooretamys angouya* (J; UFSC 6031), and *Tanyuromys thomasleei* (K; MECN 6179). Abbreviations: bf, bordering fold; eo, esophageal opening. All figures are shown at approximately the same scale. Photographs by J. Brito (A, F, G, I, K), J. Alves de Oliveira (B, H), M. Hidalgo-Cossio (C), U. Pardiñas (D, L), and J. Cherem (E, J).

Oryzomyini (examined: four clades, 24 genera, 42 species; Figs. 11–13). This tribe, the most speciose among sigmodontines, includes almost exclusively species with equiglandular stomachs. Subtle variations in whether the bordering fold aligns precisely with the main longitudinal axis of the organ or is slightly displaced to the left appear to result primarily from differences in the degree of expansion of the examined specimens. As clear exceptions to this general pattern, one member of clade C (*Oligoryzomys*; Figs. 12C, 12D) and several representatives of clade D (*e.g*., *Nectomys*, *Sigmodontomys*, *Sooretamys*; Figs. 13G, 13I, 13J) exhibit glandular epithelium extending beyond the esophageal opening toward the left side. *Holochilus* (clade D) is the only genus displaying a distinct configuration, being supraglandular, with the glandular epithelium covering most of the *corpus* and the cornified, plicate epithelium restricted to the apical region of the *corpus* (fornix ventricularis; Figs. 13C, 13D, 13L). In general, the incisura angularis is very shallow—except in a few genera such as *Aegialomys* (Fig. 13A) and *Lundomys* (Fig. 13E)—and the bordering fold runs mostly straight, forming a roughly squared outline around the esophageal opening (Figs. 12E, 12F). As expected from its supraglandular condition, *Holochilus* exhibits an extended and morphologically irregular bordering fold (Fig. 13L). Overall, the pars pylorica is indistinct and largely confluent with the remainder of the antrum.

Remarks. Some oryzomyine genera not examined here (*i.e*., *Drymoreomys*, *Handleyomys*, *Nesoryzomys*, and *Oryzomys*) have been described as equiglandular (Carleton, 1973; Patton & Hafner, 1983; Carleton & Musser, 1989; Voss, Gómez-Laverde & Pacheco, 2002; Percequillo, Weksler & Costa, 2011). For *Amphinectomys*, *Eremoryzomys*, and *Microakodontomys*, no material suitable for assessing the digestive system is currently available in collections. Tribe-focused studies (*e.g*., Carleton & Musser, 1989; Weksler, 2006) have distinguished two main conditions in oryzomyine stomachs, depending on whether the glandular epithelium is confined to the antrum or extends to cover the proximal portion of the *corpus*. Voss & Carleton (1993) emphasized the supraglandular condition of *Holochilus*, in contrast to the presumably phylogenetically close *Lundomys*.

**Figure 14:**
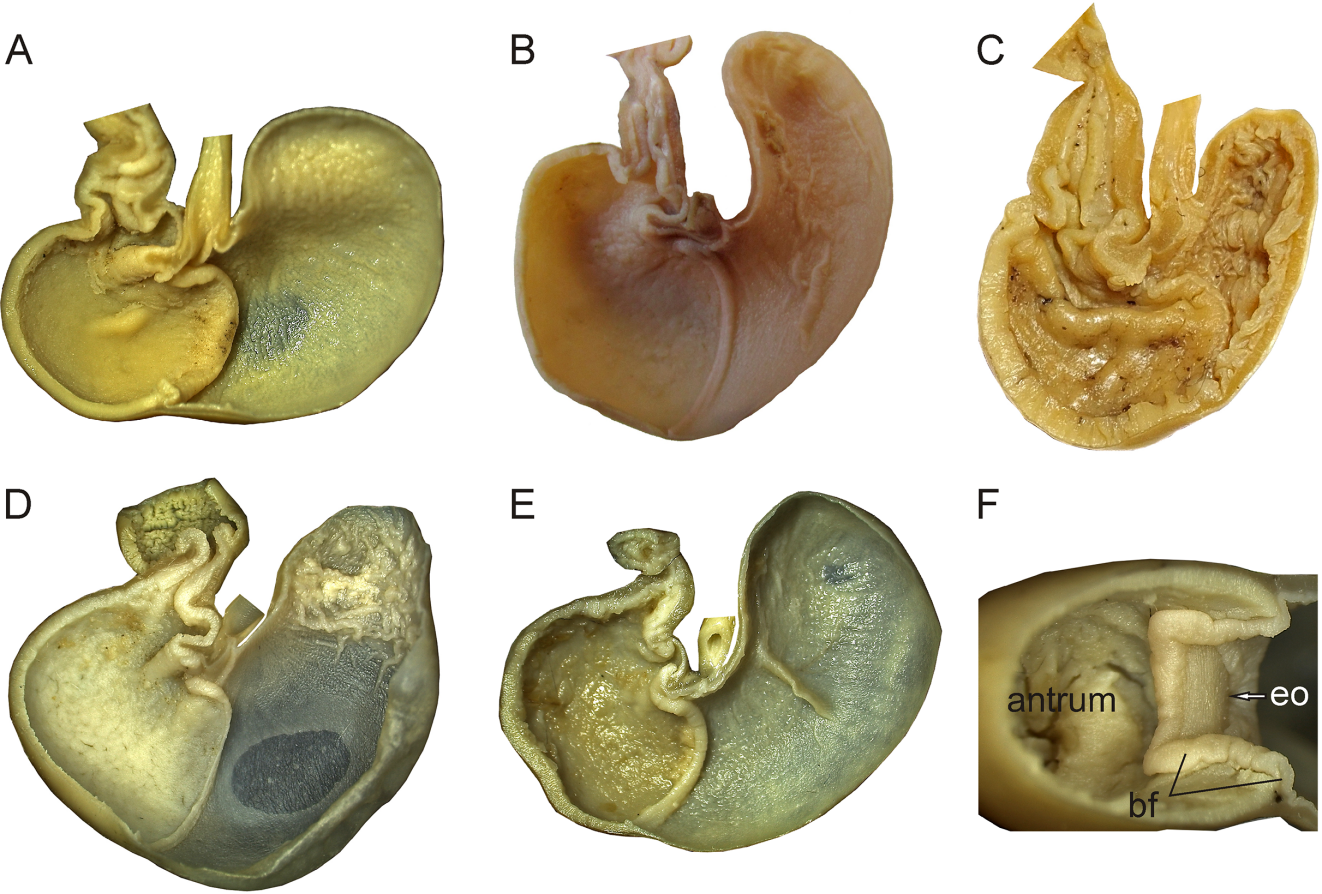
Internal stomach views of Phyllotini (Calomyina). Ventral views (A–E) and an anterior view (F): *Calassomys apicalis* (A; CNP 3437), *Calomys fecundus* (B; CNP 6806), *Calomys frida* (C; RCC 438), *Calomys laucha* (D; CNP-D 296; F; CNP 7598), and *Calomys musculinus* (E; CNP-D 367). Abbreviations: bf, bordering fold; eo, esophageal opening. All figures are shown at approximately the same scale. Photographs by U. Pardiñas (A, B, D–F) and R. Cairampoma (C).

**Figure 15:**
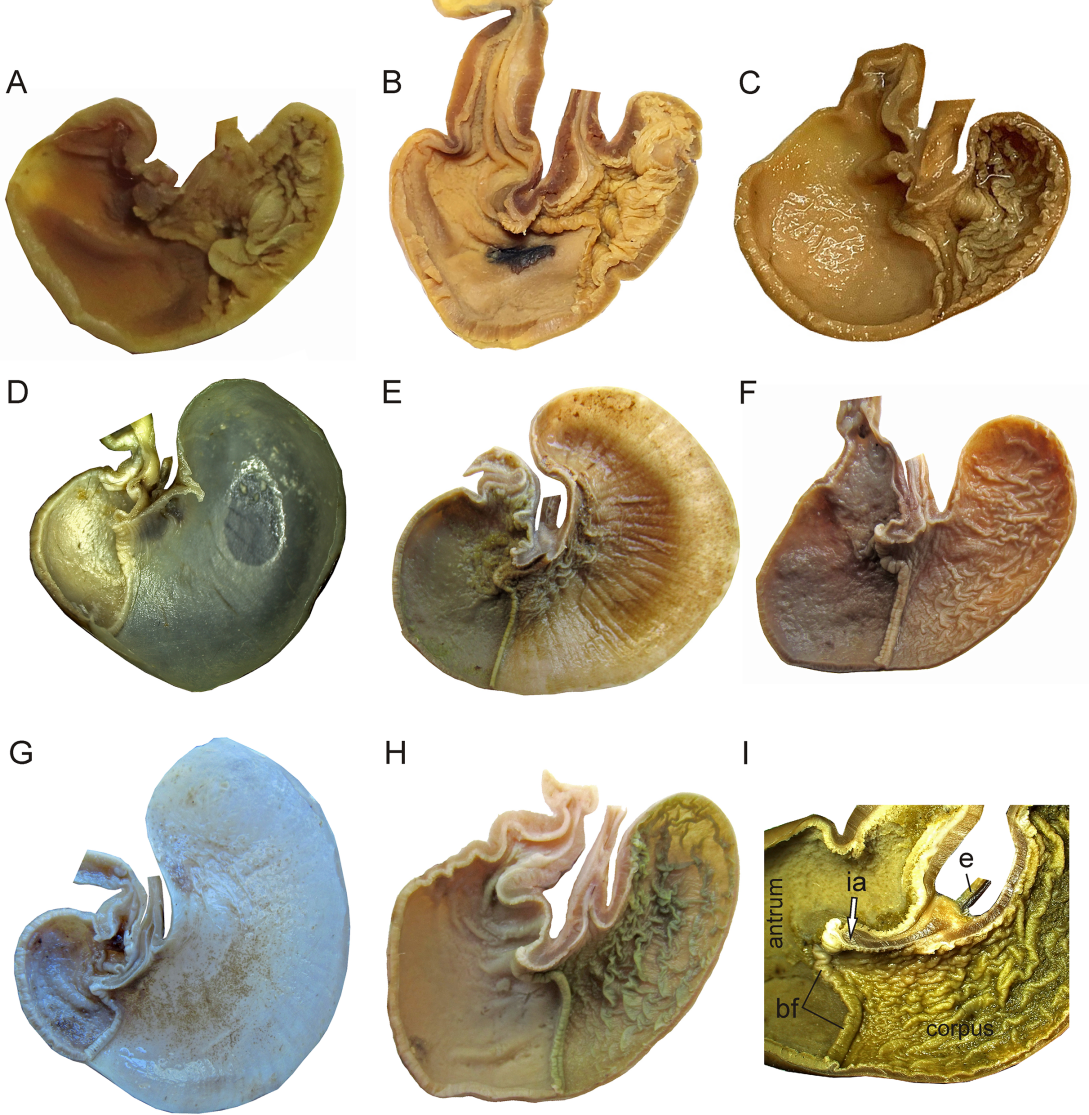
Internal stomach views of Phyllotini (Phyllotina). Ventral views: *Auliscomys boliviensis* (A; RCC 475), *Auliscomys pictus* (B; RCC 470), *Auliscomys sublimis* (C; CBF 9563), *Eligmodontia typus* (D; CNP 7650), *Graomys chacoensis* (E; CNP 6632), *Loxodontomys micropus* (F; CNP-D 213), *Phyllotis vaccarum* (G; CNP-D 326), *Tapecomys primus* (H; CNP 6607), and *Graomys griseoflavus* (I; CNP 5411). Abbreviations: bf, bordering fold; e, esophagus; ia, incisura angularis. All figures are shown at approximately the same scale. Photographs by R. Cairampoma (A, B), M. Hidalgo-Cossio (C), and U. Pardiñas (D–I).

**Figure 16:**
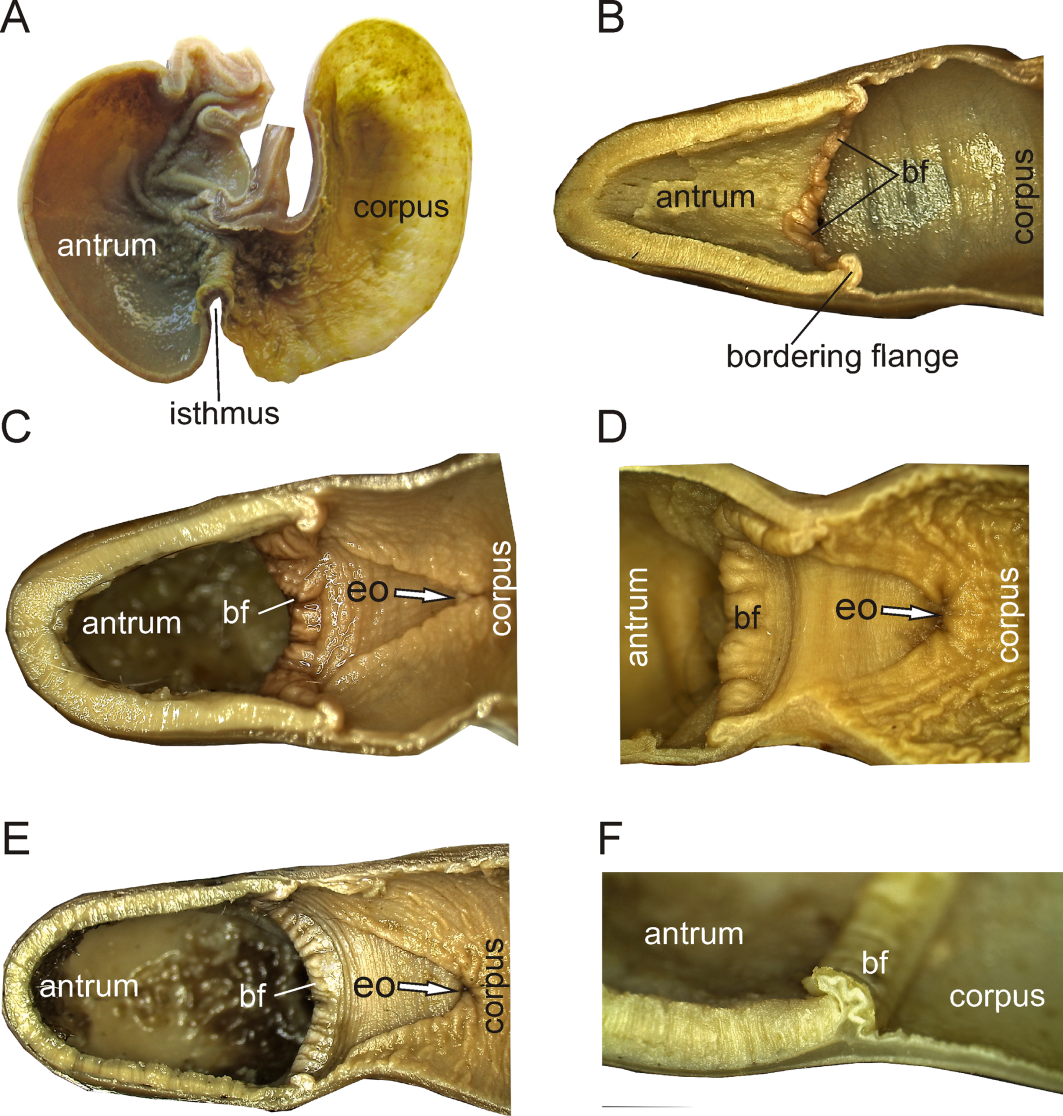
Internal stomach views of Phyllotini (Phyllotina). Ventral (A), posterior (B, F), and anterior (C–E) views: *Graomys chacoensis* (A; CNP 6638), *Eligmodontia morgani* (B, C; CNP-D 471), *Loxodontomys micropus* (D; CNP 5338), *Phyllotis xanthopygus* (E; CNP-D 424), and *Phyllotis vaccarum* (F; CNP-D 326). Abbreviations: bf, bordering fold; eo, esophageal opening. All figures are shown at approximately the same scale. Photographs by U. Pardiñas.

Phyllotini (examined: two subtribes, eight genera, 23 species; Figs. 14–16). All examined stomachs conform to the equiglandular condition, with the internal lining of the organ approximately equally divided between glandular and cornified epithelia. The less diverse subtribe Calomyina (Fig. 14) tends to exhibit smoother cornified surfaces than Phyllotina, the latter being characterized by plicate, accordion-like, or reticulate textures (Fig. 15). Both subtribes share a distinctive type of bordering fold that runs mostly straight from the plica angularis to the midpoint of the greater curvature. First, this bordering fold forms a pronounced relief resembling a flap, with its free margin bent to the right (Fig. 16F). Second, whereas in Calomyina the bordering fold appears as a thick but smooth cord, in Phyllotina its surface is conspicuously corrugated anteroposteriorly, showing a delaminated condition in some genera (*e.g*., *Loxodontomys*, *Tapecomys*; Figs. 15F, 15H). Third, transverse sections of the bordering fold reveal the presence of an accordion-like muscular layer (Fig. 16F). Some specimens exhibit an isthmus located to the right of the bordering fold, a condition that underscores the high expansion capacity of the *corpus* (Fig. 16A). Overall, members of this tribe are characterized by a deeply penetrating incisura angularis, contrasting with a shallow incisura cardialis (*e.g*., Fig. 15H). Additionally, the bordering fold surrounding the esophageal opening forms a triangular outline, with the antral margin concealing along the plica angularis (Figs. 16C, 16D).

**Figure 17:**
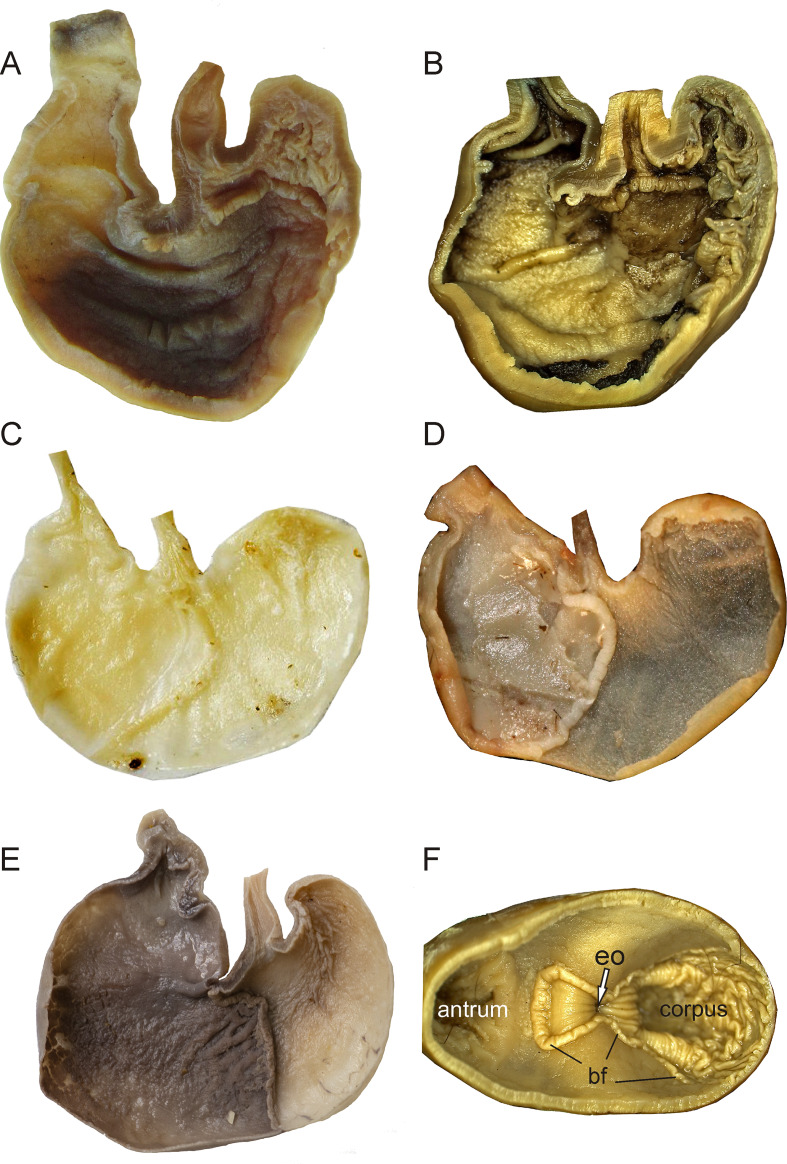
Internal stomach views of Reithrodontini, Rhagomyini, and Sigmodontini. Ventral views (A–E) and an anterior view (F): *Reithrodon auritus* (A; CNP 6309; B; CNP 7018; F; CNP 5441), *Rhagomys septentrionalis* (C; MECN 6172), *Rhagomys rufescens* (D; GL 909), and *Sigmodon peruanus* (E; MECN 4184). Abbreviations: bf, bordering fold; eo, esophageal opening. All figures are shown at approximately the same scale. Photographs by U. Pardiñas (A, B, F), J. Brito (C, E), and G. Lessa (D).

Reithrodontini (examined: one genus, one species; Figs. 17A, 17B). This emblematic monotypic tribe exhibits a supraglandular stomach, with almost the entire internal surface lined by glandular epithelium and the cornified epithelium restricted to a small portion of the fornix ventricularis. In that region, the cornified surface forms lamellae resembling the condition observed in Euneomyini. Moreover, the stomach of *Reithrodon* is comparatively small relative to the overall body size. Both principal incisurae—angularis and cardialis—are poorly developed. Finally, the configuration of the bordering fold around the esophageal opening displays the outline typical of supraglandular stomachs (cf. *Holochilus*; Fig. 17F).

Rhagomyini (examined: one genus, two species; Figs. 17C, 17D). Both examined species exhibit equiglandular stomachs that are externally shepherd’s bag-shaped, formed by the conjunction of two very shallow main incisurae and equally developed antral and corporal regions. The bordering fold runs medially curved from the right side, slightly surpassing the imaginary line determined by the esophageal opening.

Remarks. The two species examined likely represent different genera (see Pardiñas et al., 2022). A third species not examined here, *R. longilingua*, also possesses an equiglandular stomach (Luna & Patterson, 2003: fig. 11).

Sigmodontini (examined: one genus, one species; Fig. 17E). The type tribe of the subfamily exhibits an equiglandular configuration. The incisura angularis is more pronounced than the incisura cardialis, and the bordering fold runs straight, intersecting the greater curvature.

Remarks. Although poorly represented in our sample, the stomach of *Sigmodon*—a genus comprising 13 extant species (Patton, 2017)—has been described in several studies, particularly those focused on the North American *Sigmodon hispidus* (Blank, 1950; Vorontsov, 1962; Carleton, 1973). All authors reported an equiglandular, morphologically unremarkable stomach.

**Figure 18:**
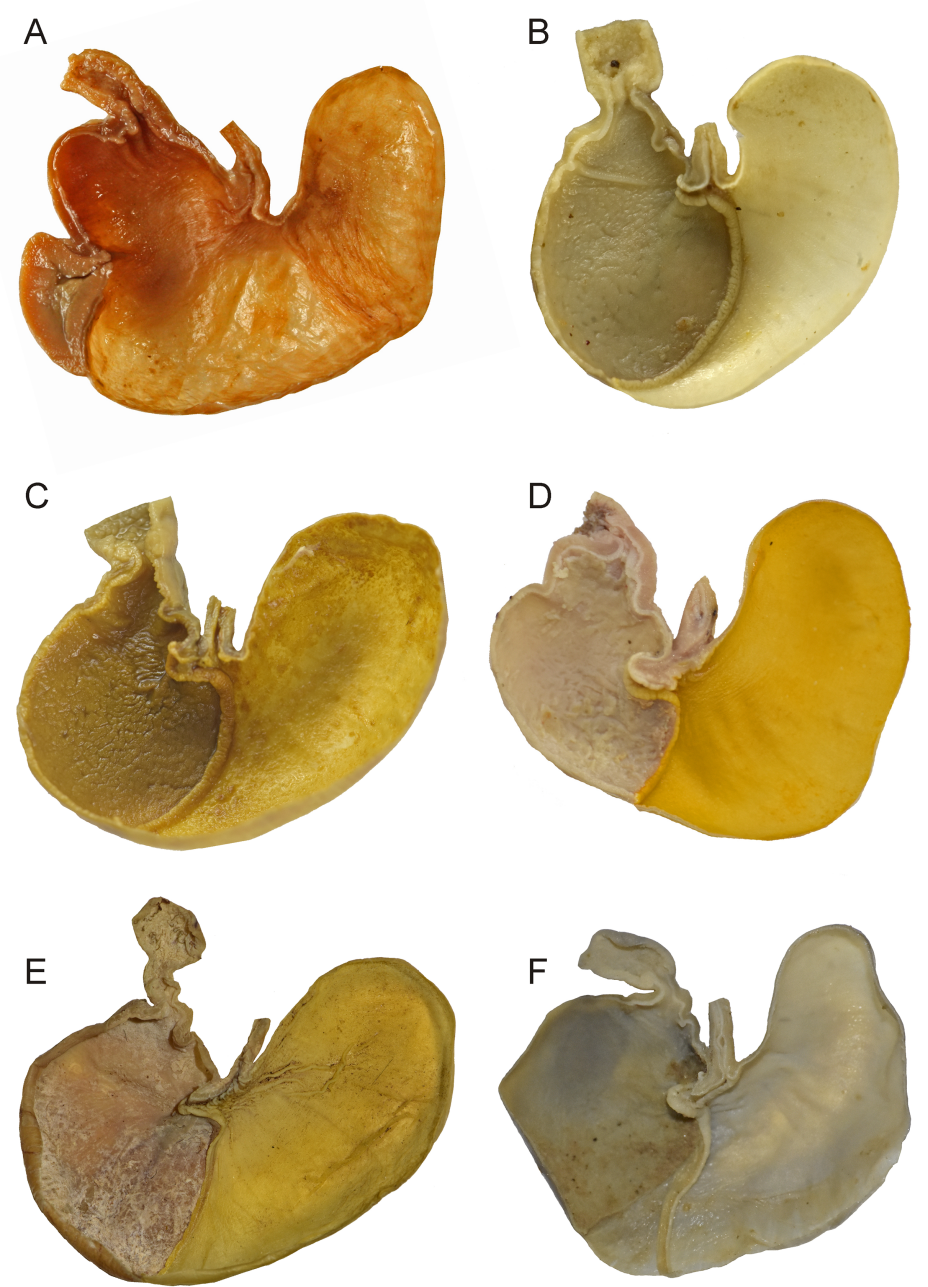
Internal stomach views of Thomasomyini. Ventral views: *Aepeomys lugens* (A; CVULA I-1057), *Chilomys georgeledecii* (B; MECN 6205), *Chilomys percequilloi* (C; MECN 6098), *Rhipidomys sp.* (D; MECN 8125), Rhipidomys cf. *R. latimanus* (E; MECN 5934), and *Rhipidomys leucodactylus* (F; MECN 7147). All figures are shown at approximately the same scale. Photographs by B. Rivas (A) and J. Brito (B–F).

**Figure 19:**
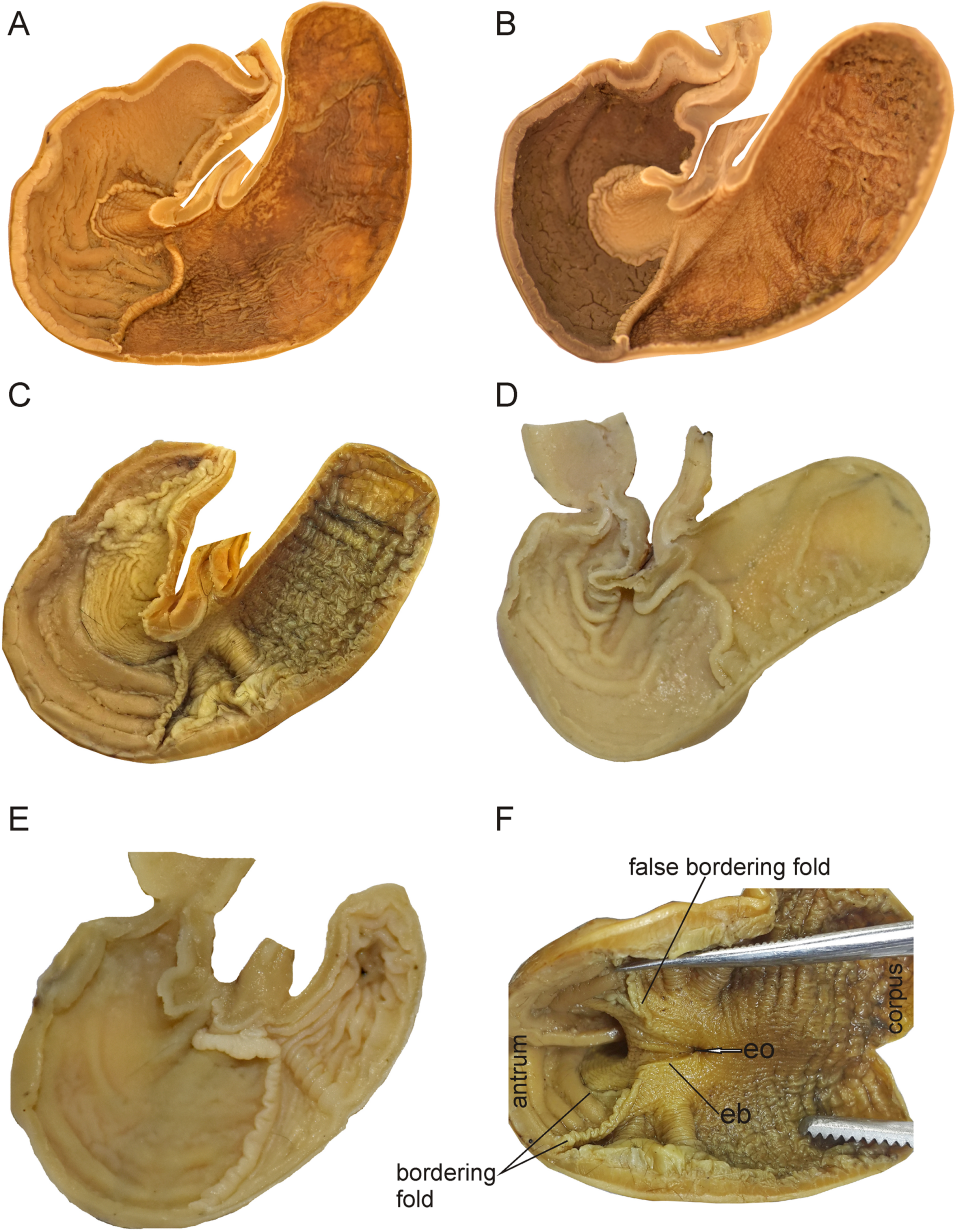
Internal stomach views of Thomasomyini, genus Thomasomys (aureus and baeops groups). Ventral views (A–E) and an anterior view (F): *Thomasomys cf.* *T. aureus* (A; MECN 6500), *Thomasomys burneoi* (B; MECN 7263), *Thomasomys sp.* (C, F; CBF 9510), *Thomasomys baeops* (D; MECN 7694), and *Thomasomys taczanowskii* (E; MECN 6827). Abbreviations: ef, esophageal bordering fold; eo, esophageal opening. All figures are shown at approximately the same scale. Photographs by J. Brito (A, B, D, E) and M. Hidalgo-Cossio (C, F).

**Figure 20:**
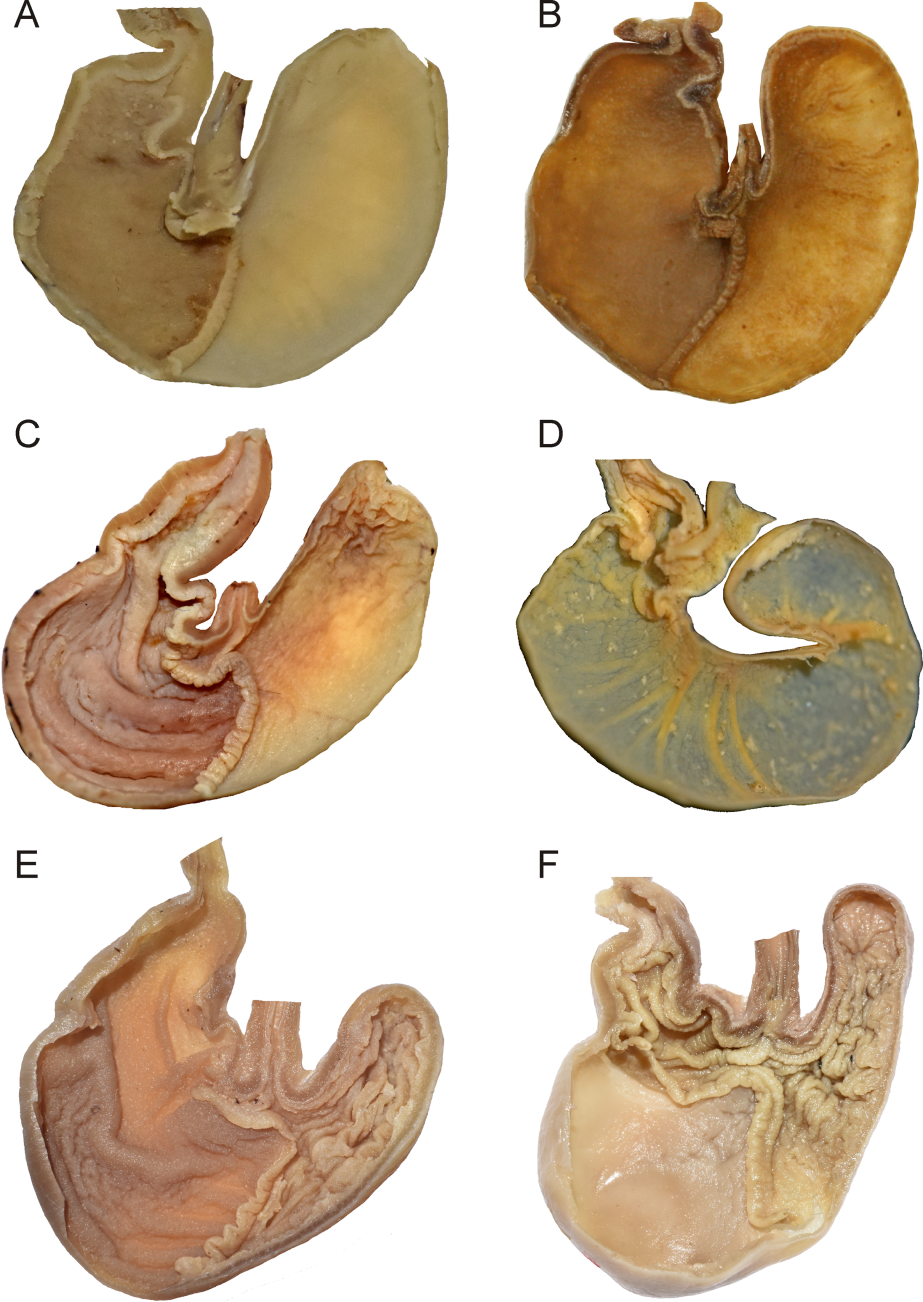
Internal stomach views of Thomasomyini, genus Thomasomys (cinereus group, in part). Ventral views: *Thomasomys cinnameus* (A; MECN 7685), *Thomasomys emeritus* (B; CVULA 4918), *Thomasomys fumeus* (C; MECN 6893), *Thomasomys hudsoni* (D; MECN 6089), *Thomasomys paramorum* (E; MECN 5277), and *Thomasomys erro* (F; MECN 6892). All figures are shown at approximately the same scale. Photographs by J. Brito (A, C–E) and B. Rivas (B).

**Figure 21:**
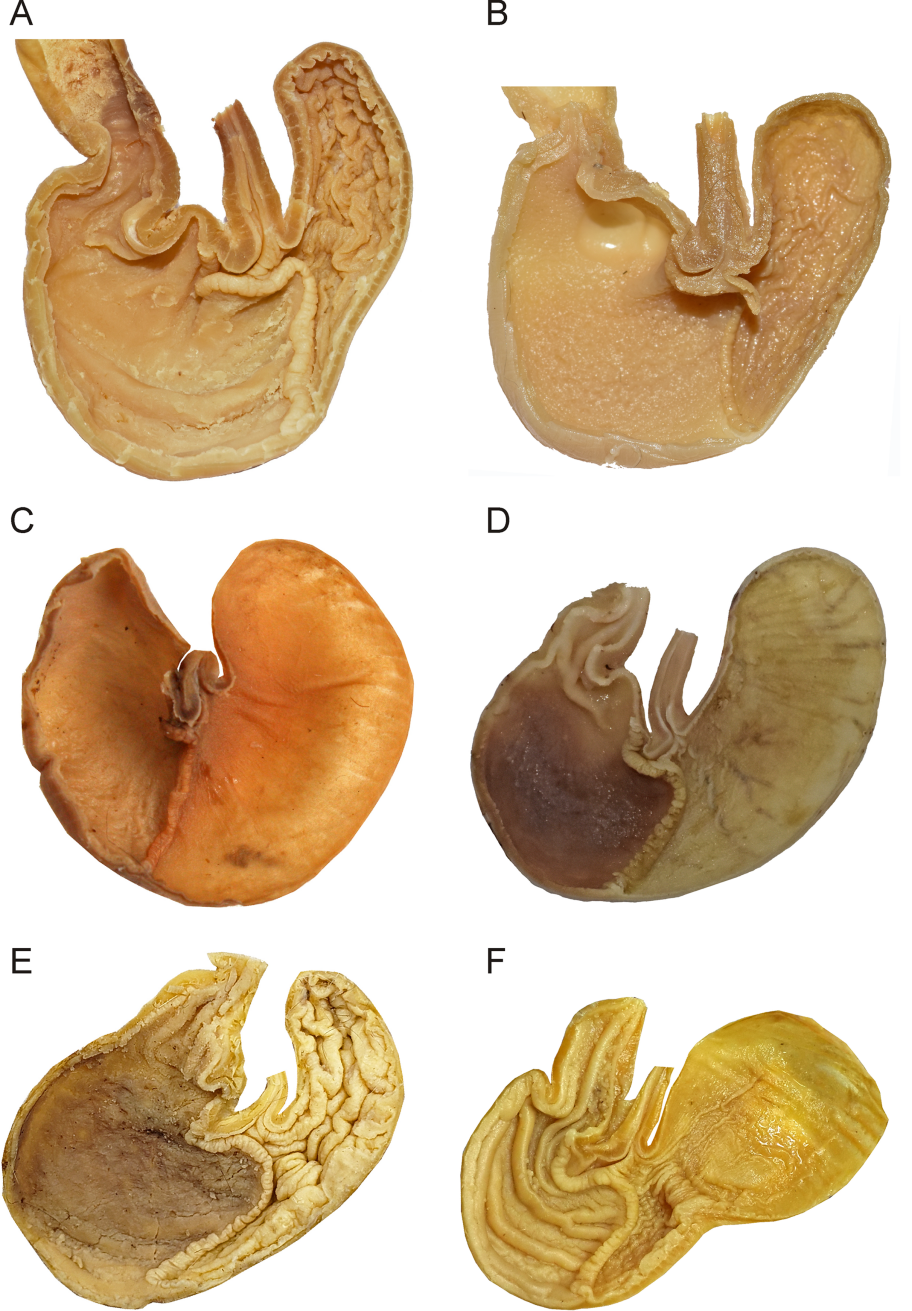
Internal stomach views of Thomasomyini, genus Thomasomys (cinereus [in part], gracilis, and incanus groups). Ventral views: Thomasomys salazari (A; MECN 5681), *Thomasomys silvestris* (B; MECN 2821), *Thomasomys vestitus* (C; CVULA 989), *Thomasomys vulcani* (D; MECN 7522), *Thomasomys andersoni* (E; CBF 9874), and *Thomasomys ladewi* (F; CBF 10209). All figures are shown at approximately the same scale. Photographs by J. Brito (A, B, D), B. Rivas (C), and M. Hidalgo-Cossio (E, F).

Thomasomyini (examined: four genera, 29 species; Figs. 18–21). This generically-poor Andean tribe exhibits a striking and interpretively challenging diversity of stomach configurations. The most widespread anatomical design is the equiglandular condition, shared by many members of three genera (*Chilomys*, *Rhipidomys*, and *Thomasomys*). However, the highly speciose *Thomasomys*—the richest sigmodontine genus at the species level—also includes one additional main configuration. It corresponds to the subglandular type and is shared, at least, by *T. aureus*, *T. burneoi*, and *T. erro* (Figs. 19A–19C, Fig. 20F). Nonetheless, these three species do not display identical conditions regarding the extent of the glandular epithelium; *T. erro* exhibits a clearly more extensive participation of cornified epithelium into the antrum than the two former species. The stomach morphology of *Thomasomys* is further remarkable because one species, *T. hudsoni*, displays a gastric anatomy that represents a previously undetected unique configuration (Fig. 20D). Apparently, glandular epithelium is entirely absent in this organ, at least as typically observed in other sigmodontines. Furthermore, the boundaries between antrum and *corpus* are indistinct, and the entire lumen appears as a broadly expanded “*corpus*.” This structural ambiguity is accentuated by the close spatial proximity of the esophagus and pylorus, which converge toward the right side. The uniqueness of the tribe extends even further, as *Aepeomys*, the fourth included genus, entirely departs from these patterns by exhibiting a diverticular configuration (Fig. 18A). In this case, the glandular pouch forms a small subtriangular “pocket” that opens directly into the pars pylorica. Moreover, and differing from the diverticular condition observed in Akodontini, the orifice connecting the diverticulum with the main stomach lumen is not minute but relatively large. With regard to the development of the main gastric incisurae, Thomasomyini display substantial variation. In *Chilomys*, the incisura angularis and incisura cardialis are unremarkable and situated at approximately the same level (Figs. 18B, 18C). In contrast, the predominant condition in *Rhipidomys* (Figs. 18D–18F) and *Thomasomys* (Figs. 19–21) involves a more pronounced incisura angularis and a moderately shallow incisura cardialis, a condition further accentuated in some subglandular species (*e.g*., *T. aureus*; Fig. 19C). The bordering fold also varies considerably within the tribe, ranging from a relatively smooth cord in *Chilomys* and *Rhipidomys* (Fig. 18), to a thick, corrugated fold in *T. fumeus* (Fig. 20C), or even a “double cord” in *T. aureus* and *T. burneoi* (Figs. 19A–19C). This latter configuration merits special attention, as its complexity results from the partial combination of two distinct folds: the classical bordering fold (*sensu* Carleton, 1973) and the posterior extension of the plica cardialis. Together, these two elements dissect the greater curvature as a conspicuous flange. It is noteworthy that in some species of *Thomasomys* (*e.g*., Figs. 20F, 21E), the bordering fold shifts to the right as it traverses the greater curvature.

Remarks. The “aglandular” condition implied by the stomach of *T. hudsoni* is particularly intriguing. Although it could correspond to a teratological specimen, no other aspect of the individual suggests such an anomaly. Considering that the present survey includes approximately 18 species of *Thomasomys*, and that at least ten additional species can be added from the literature (see Discussion), this represents roughly one-third of the genus’s estimated diversity (*Thomasomys* may encompass around 100 species; Ruelas et al., 2024). We therefore chose to describe the unique morphology of *T. hudsoni* rather than dismiss it as abnormal. Carleton (1973) was the first to recognize the striking stomach variation exhibited by *Thomasomys* and Voss, Gómez-Laverde & Pacheco (2002) briefly noted the distinctive condition of *Aepeomys*.

**Figure 22:**
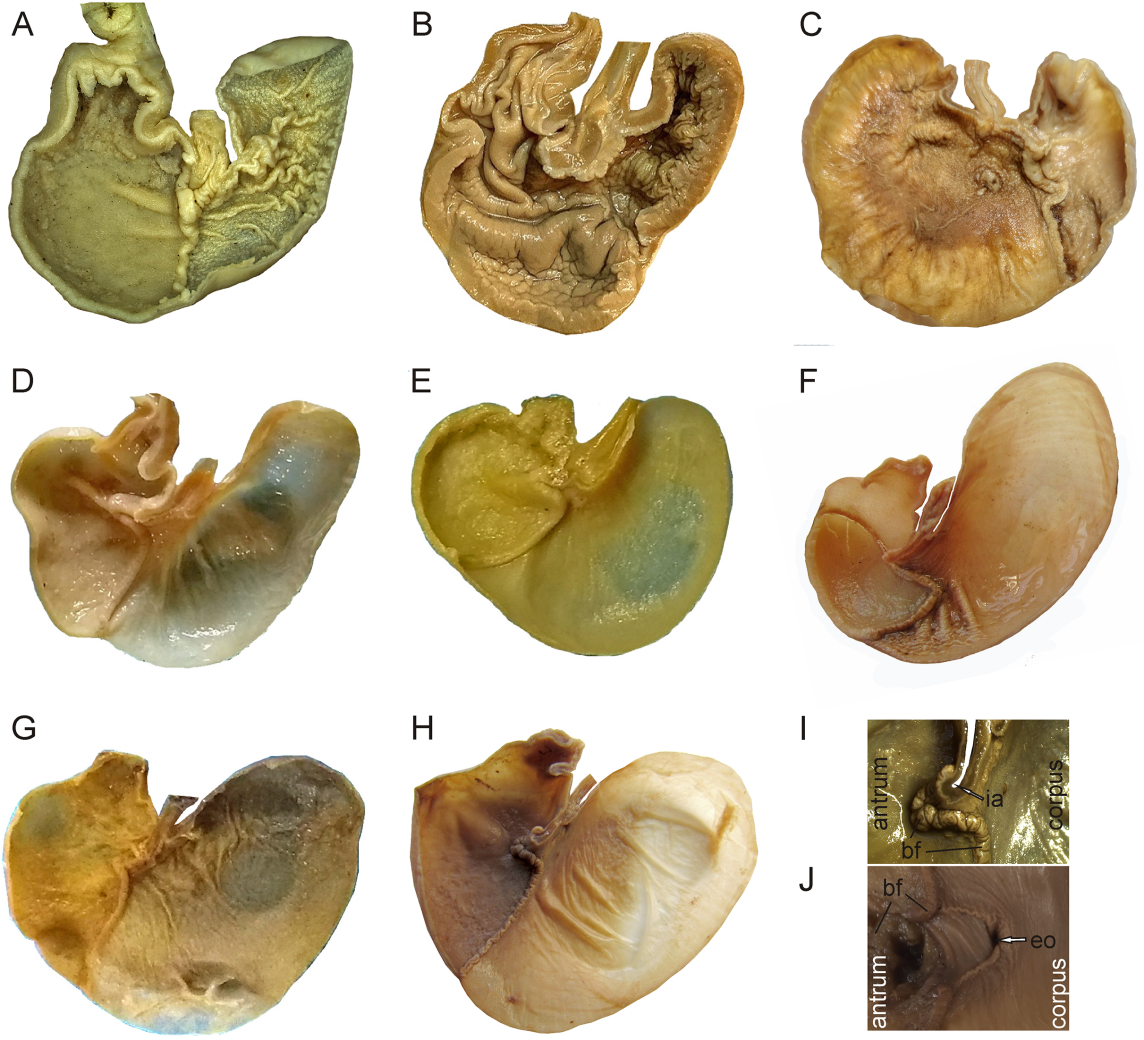
Internal stomach views of Sigmodontinae incertae sedis and Wiedomyini. Ventral views (A–I) and an anterior view (J): *Abrawayaomys chebezi* (A; CNP 3631), *Chinchillula sahamae* (B; CBF 9539; C; RCC 478), *Delomys sublineatus* (D; UFSC 5908), *Juliomys ossitenuis* (E; UFSC 5949), *Phaenomys ferrugineus* (F; CMUFLA 904), *Wiedomys pyrrhorhinos* (G, J; UFSC 5224), and *Wilfredomys oenax* (H, I; CNP-D 130). Abbreviations: bf, bordering fold; eo, esophageal opening; ia, incisura angularis. All figures are shown at approximately the same scale. Photographs by U. Pardiñas (A, F, H, I), M. Hidalgo-Cossio (B), R. Cairampoma (C), and J. Cherem (D, E, G, J).

Wiedomyini (examined: four genera, seven species; Fig. 22). The entire generic content of this tribe was examined. Three genera exhibit the equiglandular condition (Figs. 22E, 22G, 22H), whereas the remaining one (*Phaenomys*; Fig. 22F) displays a subglandular configuration. The latter also presents additional peculiarities, such as a very deep incisura angularis and a protruding fornix ventricularis. While the latter feature is comparable to examples observed in other tribes (*e.g*., Andinomyini), the depth of the incisura angularis in *Phaenomys* exceeds that of all other sigmodontines studied. This characteristic, together with the sharply delimited glandular epithelium, gives the stomach a distinctive “three-chambered” appearance (Fig. 22F). In the remaining wiedomyines, the incisura angularis is moderately penetrant, and consequently the bordering fold bends sharply to the right as it approaches the esophageal opening (Fig. 22I). The cornified epithelium appears smooth.

Remarks. The unique development of the incisura angularis in *Phaenomys* tempts one to invoke the exception that proves the rule regarding the predominantly unilocular nature of sigmodontine stomachs (Carleton, 1973). In fact, its degree of penetration resembles that seen in neotomines, which are typically described as having bilocular stomachs (Carleton, 1973). However, since only a single specimen of this rare genus was examined, additional material is needed to confirm the true nature of this condition.

Sigmodontinae incertae sedis (examined: three genera, three species; Fig. 22). The common configuration observed is the equiglandular type. However, whereas *Abrawayaomys* (Fig. 22A) and *Delomys* (Fig. 22D) share a typical condition characterized by a more or less equal distribution of glandular and cornified epithelium between the antrum and *corpus*, this is not the case for *Chinchillula*. This Altiplanic genus exhibits a morphology resembling that of Andinomyini, with an anterior protrusion of the cornified epithelium toward the right side. This protrusion is easily discernible in partially collapsed stomachs (Fig. 22B) but tends to disappear when the organ is fully expanded (Fig. 22C)—an observation that, strictly speaking, justifies classifying this stomach as equiglandular rather than subglandular. *Chinchillula* and *Delomys* share a more pronounced incisura angularis than incisura cardialis, whereas *Abrawayaomys* exhibits a very shallow incisura angularis and no rightward displacement of the anterior portion of the bordering fold. *Abrawayaomys* and *Chinchillula* share a plicate condition of the cornified epithelium, while in *Delomys* it is smooth.

Remarks. These three genera share the taxonomic condition of being incertae sedis—and are thus described together here—but this does not imply any phylogenetic relationship among them. In fact, *Abrawayaomys* was recently proposed within Akodontini, and *Chinchillula* and *Delomys* were each recognized as constituting monotypic tribes (Bangs et al., 2025). *Chinchillula* was previously illustrated by Dorst (1972), whose observations on stomach anatomy agree with those reported here.

### Phylogenetic approach

**Figure 23:**
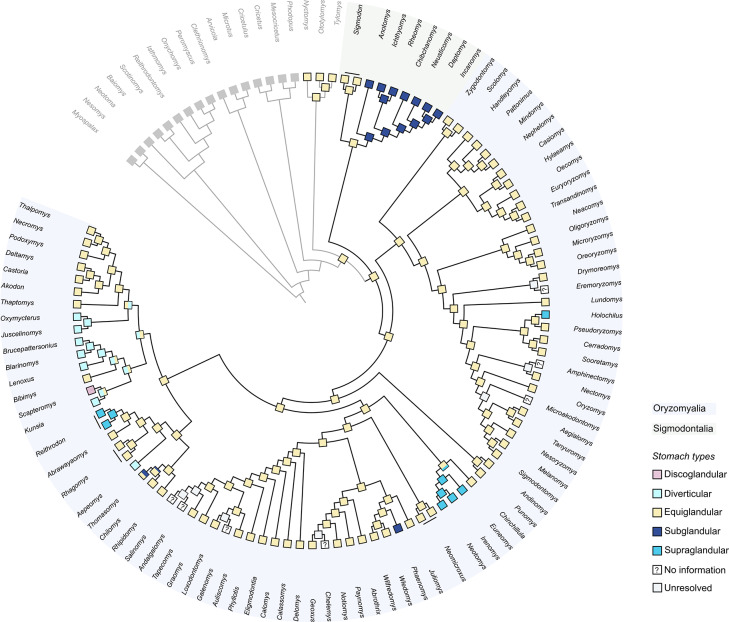
Ancestral state reconstruction of major stomach types in Sigmodontinae. Colors correspond to the five principal gastric designs, and squares at nodes indicate proportional likelihood estimates of ancestral states across the subfamily (see Table S3).

**Figure 24:**
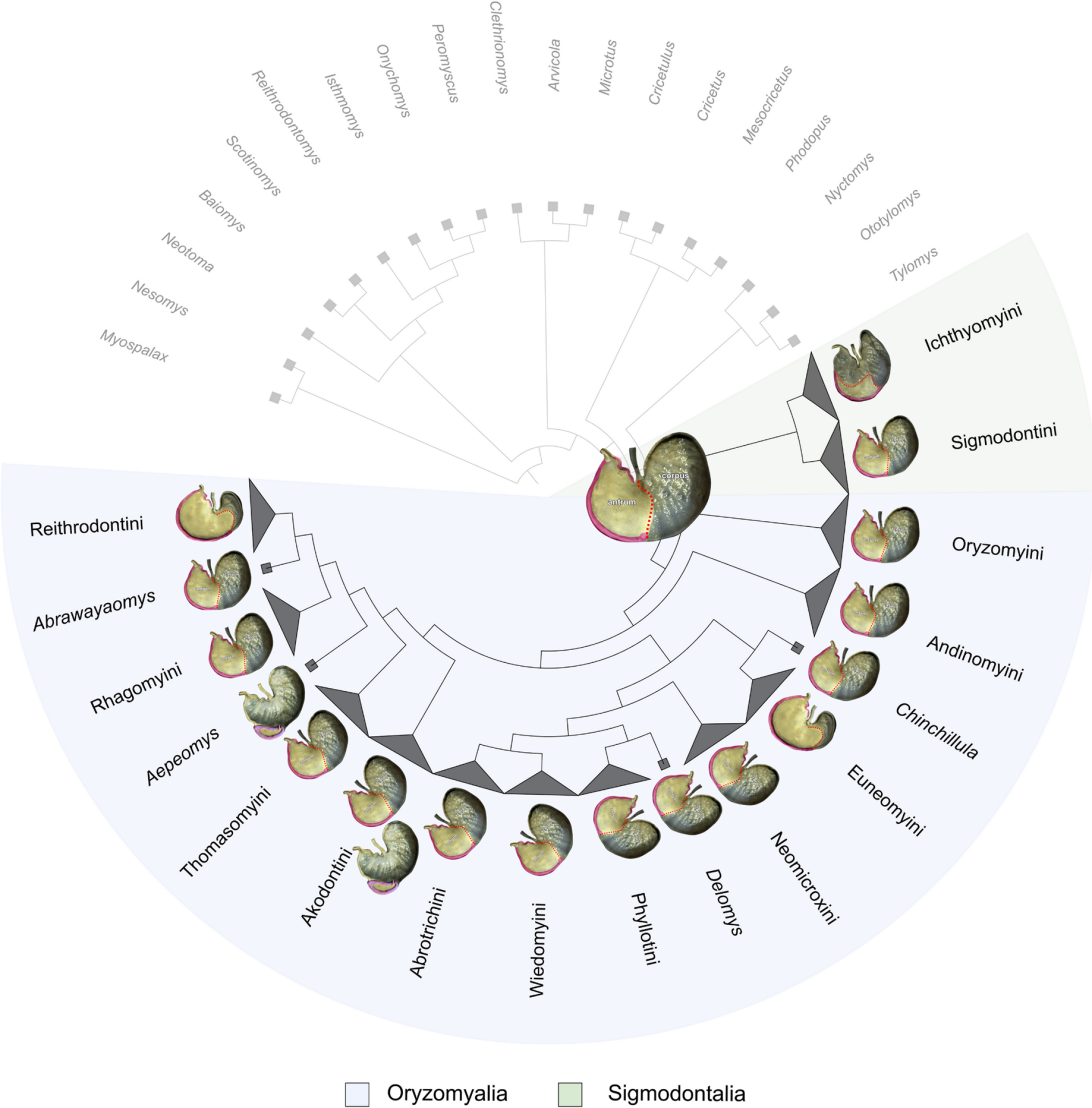
Collapsed phylogeny of Sigmodontinae showing the most likely ancestral stomach type inferred for each tribe. Tribes are collapsed, and the inferred most probable ancestral stomach type is indicated (silhouettes as in Fig. 3). The two major clades, Oryzomyalia (blue shading) and Sigmodontalia (green shading), are shown for reference.

Maximum Likelihood analyses support the monophyly of Sigmodontinae, with Tylomyinae recovered as its sister group. Within Sigmodontinae, two major clades—Oryzomyalia and Sigmodontalia—were identified (Figs. 23, 24), in agreement with previous multilocus phylogenies (*e.g*., Pardiñas et al., 2024, 2022; Gonçalves et al., 2020; Schenk & Steppan, 2018). All tribes were recovered as monophyletic, with the exception of Thomasomyini (Fig. 23). Taxa traditionally considered *incertae sedis* were retrieved as independent lineages rather than nested within Akodontini or Phyllotini, as previously suggested for genera such as *Abrawayaomys* and *Delomys* (*e.g*., Gonçalves et al., 2020; Bangs et al., 2025). Support for the deepest nodes (*i.e*., Sigmodontinae, Sigmodontalia, and Oryzomyalia) reached maximal bootstrap values (BT = 100), whereas nodes defining individual tribes were also strongly supported (BT = 90–100).

Mapping stomach morphology onto the sigmodontine phylogeny revealed a heterogeneous pattern across the subfamily (Fig. 23). The equiglandular condition is the most widespread, occurring in multiple tribes within both Sigmodontalia and Oryzomyalia. In contrast, the subglandular and supraglandular gastric designs show a more restricted distribution, occurring predominantly in ichthyomyine, euneomyine, and reithrodontine lineages, where they represent derived conditions. The diverticular type, comparatively rare, was detected only in akodontines and *Aepeomys*. *Scapteromys* is the only representative exhibiting a discoglandular stomach.

Maximum Likelihood reconstructions indicate that the equiglandular condition is the most probable ancestral stomach type for several extant tribes and for the base of Sigmodontinae (Table S3). Deep nodes favor this state over alternative configurations, suggesting that the early diversification of the group was characterized by a relatively generalized stomach anatomy. Independent transitions to other gastric configurations are inferred in tribes such as Akodontini, Euneomyini, Ichthyomyini, and Reithrodontini, indicating that specialized stomach types evolved under particular ecological or dietary contexts during sigmodontine evolution.

## Discussion

### Intrageneric gastric variability

This study was not designed specifically to evaluate intrageneric variation. However, 33 of the examined genera were represented by more than one species (Table 1). Among those with five or more sampled species are *Thomasomys* (18 species), *Akodon* (14), *Phyllotis* (7), *Oxymycterus* (6), *Calomys* (5), *Ichthyomys* (5), *Necromys* (5), and *Oligoryzomys* (5). This sample indicates that, in at least four tribes (*i.e*., Akodontini, Ichthyomyini, Oryzomyini, and Phyllotini), congeners typically share the same stomach type. Even in genera whose species inhabit markedly different environments, the main stomach design appears to be a generic trait. For example, examined species of *Oxymycterus* (*e.g*., Hershkovitz, 1994; Oliveira & Gonçalves, 2015)—including forms from Rioplatan grasslands (*O. nasutus*, *O. rufus*; Tullberg, 1899; Vorontsov, 1962, 1967; Carleton, 1973; Pardiñas et al., 2020), Atlantic forests (*O. quaestor*; Pardiñas et al., 2020), montane forests (*O. akodontius*; Pardiñas et al., 2020), high-Andean grasslands (*O. hiska*; Pardiñas et al., 2020), and Cerrado shrublands (*O. itapeby*; Bezerra et al., 2025)—all exhibit the same basic diverticular stomach type first described by Tullberg (1899).

The only clear exception to this intrageneric conservatism is *Thomasomys*, the most species-rich sigmodontine genus (*e.g*., Brito et al., 2024; Ruelas et al., 2024; Brito & Pardiñas, 2025). At least two stomach types have been documented within this genus (*e.g*., Carleton, 1973; Pacheco & Ruelas, 2023; Brito et al., 2024; this study). Most examined species possess equiglandular stomachs, but some are subglandular; at least one case represents a condition in which glandular epithelium is apparently absent (“aglandular”). In addition, among the five species of *Necromys* analyzed, *N. obscurus* shows a tendency toward a supraglandular condition, diverging from the remaining congeners, which typically exhibit equiglandular stomachs (Pardiñas et al., 2020).

Although the available data are insufficient for definitive conclusions, evidence on sigmodontine stomach morphology suggests that the main anatomical design is largely invariant within genera (*e.g*., Vorontsov, 1962, 1967; Carleton, 1973; Pardiñas et al., 2020; this study). Similar patterns occur in other species-rich muroid groups. In his study of Arvicolinae, Carleton (1981) found that most genera exhibit a single predominant stomach morphology (*i.e*., discoglandular; see also Vorontsov, 1967: fig. 118). The only detected exception is *Myodes*, which presents at least two types; within that genus, a single species, *M. gapperi*, includes both subglandular and discoglandular stomachs (Carleton, 1981: fig. 5).

The gastric diversity within *Thomasomys* was first noted by Carleton (1973), who identified it as the only sigmodontine genus showing intrageneric variation in stomach morphology. In particular, Carleton (1973) emphasized the contrast between the subglandular condition of a single specimen of *T. aureus* and that of six other species examined. Carleton (1973: 28) noted that “if further evidence on gastric morphology reveals a pervasive hemiglandular pattern [in other species of *Thomasomys*], then the distinctive features of the glans penis and stomach of *aureus* argue strongly for its separation from *Thomasomys*.” Pacheco (2003) similarly highlighted the distinctive gastric anatomy of this species relative to other *Thomasomys* and Thomasomyini. Subsequent studies have expanded this pattern. Stomach morphology is now known for at least 27 species of *Thomasomys*, representing approximately 50% of recognized species (Table 4). While some species groups are relatively well sampled (*e.g*., the *cinereus* group, with data for 15 of 29 species), others remain poorly represented (*e.g*., the *aureus* group, 2 of 11 species). The subglandular condition remains characteristic of the *aureus* group, as originally noted by Carleton (1973), but also occurs in members of at least two other groups (*i.e*., *apeco* and *cinereus*). The equiglandular condition appears widespread across at least five groups (Table 4). The *apeco* case is notable, as its gastric similarity to *aureus* was recognized by Leo & Gardner (1993) prior to phylogenetic analyses; subsequent genetic evidence supports a close relationship between *apeco* and the *aureus* complex (*e.g*., Ruelas et al., 2024).

**Table 4:** Known diversity of gastric morphology within Thomasomys.

Species	Group	Stomach	Main reference
*Thomasomys apeco* Leo & Gardner, 1993	Apeco	Subglandular	Leo & Gardner (1993)
*Thomasomys antoniobracki* Ruelas & Pacheco, 2021	Aureus		
*Thomasomys aureus* (Tomes, 1860)	Aureus	Subglandular	Carleton (1973)
*Thomasomys auricularis* Anthony, 1923	Aureus		
*Thomasomys burneoi* Lee, Tinoco & Brito, 2022	Aureus	Subglandular	This article
*Thomasomys nicefori* Thomas, 1921	Aureus		
*Thomasomys pardignasi* Brito et al., 2021	Aureus		
*Thomasomys popayanus* Allen, 1912	Aureus		
*Thomasomys praetor* (Thomas, 1900)	Aureus		
*Thomasomys princeps* (Thomas, 1895)	Aureus		
*Thomasomys pyrrhonotus* (Thomas, 1886)	Aureus		
*Thomasomys rosalinda* Thomas & St. Leger, 1926	Aureus		
*Thomasomys baeops* (Thomas, 1899)	Baeops	Equiglandular	This article
*Thomasomys taczanowskii* (Thomas, 1882)	Baeops	Equiglandular	This article
*Thomasomys australis* Anthony, 1925	Cinereus		
*Thomasomys bombycinus* Anthony, 1925	Cinereus		
*Thomasomys caudivarius* Anthony, 1923	Cinereus	Equiglandular	This article
*Thomasomys cinereiventer* Allen, 1912	Cinereus		
*Thomasomys cinereus* (Thomas, 1882)	Cinereus	Equiglandular	Carleton (1973)
*Thomasomys cinnameus* Anthony, 1924	Cinereus	Equiglandular	This article
*Thomasomys contradictus* Anthony, 1925	Cinereus		
*Thomasomys daphne* Thomas, 1917	Cinereus		
*Thomasomys dispar* Anthony, 1925	Cinereus		
*Thomasomys emeritus* Thomas, 1916	Cinereus	Equiglandular	This article
*Thomasomys erro* Anthony, 1926	Cinereus	Subglandular	This article
*Thomasomys fumeus* Anthony, 1924	Cinereus	Equiglandular	This article
*Thomasomys hudsoni* Anthony, 1923	Cinereus	"Aglandular"	This article
*Thomasomys hylophilus* Osgood, 1912	Cinereus	Equiglandular	Carleton (1973)
*Thomasomys igor* Brito et al., 2024	Cinereus		
*Thomasomys laniger* (Thomas, 1895)	Cinereus		
*Thomasomys lojapiuranus* Pacheco & Ruelas, 2023	Cinereus	Equiglandular	Pacheco & Ruelas (2023)
*Thomasomys monochromos* (Bangs, 1900)	Cinereus		
*Thomasomys niveipes* (Thomas, 1896)	Cinereus		
*Thomasomys onkiro* Luna & Pacheco, 2002	Cinereus		
*Thomasomys otavalo* Brito et al., 2024	Cinereus	Equiglandular	Brito et al. (2024)
*Thomasomys pagaibambensis* Pacheco & Ruelas, 2023	Cinereus	Equiglandular	Pacheco & Ruelas (2023)
*Thomasomys paramorum* Thomas, 1898	Cinereus	Equiglandular	Carleton (1973)
*Thomasomys salazari* Brito et al., 2019	Cinereus	Equiglandular	This article
*Thomasomys shallqukucha* Pacheco & Ruelas, 2023	Cinereus	Equiglandular	Pacheco & Ruelas (2023)
*Thomasomys silvestris* Anthony, 1924	Cinereus	Equiglandular	This article
*Thomasomys ucucha* Voss, 2003	Cinereus	Equiglandular	Voss (2003)
*Thomasomys vestitus* (Thomas, 1898)	Cinereus	Equiglandular	This article
*Thomasomys vulcani* (Thomas, 1898)	Cinereus	Equiglandular	Carleton (1973)
*Thomasomys andersoni* Salazar-Bravo & Yates, 2007	Gracilis	Equiglandular	This article
*Thomasomys gracilis* Thomas, 1917	Gracilis		
*Thomasomys oreas* Anthony, 1926	Gracilis		
*Thomasomys eleusis* Thomas, 1926	Incanus		
*Thomasomys incanus* (Thomas, 1894)	Incanus		
*Thomasomys ischyrus* Osgood, 1914	Incanus	Equiglandular	Carleton (1973)
*Thomasomys kalinowskii* (Thomas, 1894)	Incanus		
*Thomasomys ladewi* Anthony, 1926	Incanus	Equiglandular	This article
*Thomasomys macrotis* Gardner & Romo, 1993	Incanus		
*Thomasomys notatus* Thomas, 1917	Notatus	Equiglandular	Pacheco (2003)
Total (species/known stomachs)	53	27	

In summary, although intrageneric variation is uncommon, it is not unprecedented and does not necessarily indicate that a genus is artificial. However, in the case of *Thomasomys*, the accumulation of pronounced morphological differences suggests that the genus may require division into smaller, more cohesive units (*e.g*., Pacheco, 2003, 2015; Brito & Pardiñas, 2025). Comparable cases can be found in Neotominae, as documented by Carleton (1973). One example is *Reithrodontomys*, in which stomach morphology ranges from equiglandular to discoglandular (Carleton, 1973: fig. 8). Another is *Peromyscus*, which predominantly exhibits a discoglandular condition but includes a distinct diverticular type in members of the *mexicanus* species group (Carleton, 1973: fig. 9). Recent integrative taxonomic revisions have led to substantial changes in the classification of this subfamily (*e.g*., Castañeda-Rico et al., 2020; Kelly et al., 2023). In this context, subgenera long recognized within these genera have been elevated to generic rank (*e.g*., Castañeda-Rico et al., 2024), eliminating the apparent intrageneric gastric variation noted in earlier studies.

### Intratribal and intertribal gastric variability

Table 5 summarizes the main anatomical stomach designs recorded for each tribe, subtribe, or suprageneric group. Most sigmodontine tribal clades are characterized by a single stomach type (Fig. 24). Even the most speciose group, Oryzomyini—which includes 31 extant genera (*e.g*., Brito et al., 2020; Percequillo et al., 2021; Voss, 2024)—consists largely of equiglandular stomachs, with *Holochilus* as the only supraglandular exception. Similarly low variation is observed in other well-defined, though less diverse, tribes such as Phyllotini (*e.g*., Salazar-Bravo, Pardiñas & D’Elía, 2013; Carrizo & Catalano, 2015), Ichthyomyini (*e.g*., Voss, 1988; Salazar-Bravo et al., 2023; Zeballos et al., 2025), and Abrotrichini (*e.g*., Cañón et al., 2014; Teta et al., 2017), each of which exhibits a single predominant gastric design.

**Table 5:** Diversity of gastric morphology within major suprageneric groups of Sigmodontinae.

Tribe/Subtribe/Clade	Gastric morphology (main design)	Ratio (design/genera)	Genera
**Abrotrichini**	1	0.2	5
Abrotrichina	1	1.0	1
Notiomyina	1	0.3	4
**Akodontini**	3	0.2	17
Akodontina	1	0.1	8
Oxymycterina	1	0.5	2
Scapteromyina	3	0.4	7
**Andinomyini**	1	0.5	2
**Euneomyini**	1	0.3	3
**Ichthyomyini**	1	0.1	7
Anotomyina	1	1.0	1
Ichthyomyina	1	0.2	6
**Neomicroxini**	1	1.0	1
**Oryzomyini**	2	0.1	31
Clade A	1	0.5	2
Clade B	1	0.1	9
Clade C	1	0.3	4
Clade D	2	0.1	16
**Phyllotini**	1	0.1	11
Calomyina	1	0.5	2
Phyllotina	1	0.1	9
**Reithrodontini**	1	1.0	1
**Rhagomyini**	1	1.0	1
**Sigmodontini**	1	1.0	1
**Thomasomyini**	3	0.7	4
**Wiedomyini**	2	0.5	4

In contrast, two suprageneric groups stand out for their diversity. One is Akodontini (*e.g*., Pardiñas et al., 2020; Cañón, 2021; Missagia et al., 2021), which contains at least three stomach types, including one exclusive condition—the discoglandular type found in *Scapteromys*—and the highest proportion of diverticular stomachs (*e.g*., Vorontsov, 1962, 1967; Carleton, 1973; Hershkovitz, 1998; Geise et al., 2008; Bezerra et al., 2007, 2025; Pardiñas et al., 2020). The other is Thomasomyini, comprising four genera (*e.g*., Pardiñas et al., 2022). Within this tribe, a dual pattern emerges. *Thomasomys* is the only sigmodontine genus known to include more than one main anatomical stomach design (see previous section). In addition, Aepeomys is the only non-akodontine genus with a diverticular stomach (Voss, Gómez-Laverde & Pacheco, 2002; Pacheco, 2003). An undescribed genus (referred to in the literature as *Thomasomys* sp. 4; see Pacheco, 2003, 2015; Ruelas et al., 2024) also belongs to this tribe and is characterized by an equiglandular stomach (Pacheco, 2003).

These observations raise a long-standing question in sigmodontine systematics: how much morphological similarity or disparity can be accommodated within a tribe? (*e.g*., Barbière et al., 2021; Pardiñas et al., 2022; Missagia et al., 2023; Bangs et al., 2025). There is no *a priori* expectation of a fixed number of stomach types per tribe, just as there are no strict limits on molar or penile variation. However, because tribes are intended to represent natural groups whose members share common attributes—one of the basic principles of classification (*e.g*., Mayr, Linsley & Usinger, 1953; Crisci & López Armengol, 1983)—a low level of internal morphological variation is generally expected. This expectation partly reflects the assumption that stomach morphology is linked to diet and that dietary strategies—major ecological axes—are often shared among members of a tribe. In addition, transitions between major stomach types are unlikely to be evolutionarily inexpensive; gastric morphology tends to be conservative within lineages (Langer, 2017). Some of these assumptions can be evaluated using the present results.

It is tempting to associate variation in major anatomical stomach designs with species richness, assuming a direct positive relationship between the number of species or genera and the number of stomach types. However, this potential correlation is not supported. Oryzomyini, the most species-rich tribe, shows two stomach types, one of which strongly predominates, whereas Thomasomyini, a comparatively species-poor tribe, includes three types. Similarly, Wiedomyini, which includes four genera and fewer than ten species, displays two contrasting stomach designs.

Within this framework, two hypotheses may account for variation in primary gastric designs among sigmodontine tribes. The first proposes that tribes differ intrinsically in their levels of gastric diversity, with some exhibiting greater morphological disparity and others remaining comparatively conservative. Such differences parallel inequalities commonly observed in biological systems, including patterns of species diversity (*e.g*., Anderson, 1975; Reig, 1989). The alternative hypothesis is that tribes exhibiting pronounced gastric heterogeneity are artefactual assemblages that combine more than one natural suprageneric group (Pardiñas et al., 2020). These hypotheses are not mutually exclusive.

If major aspects of stomach anatomy are linked to diet, then cohesive groups of genera (*i.e*., tribes) would be expected to share the same gastric configuration (*e.g*., Vorontsov, 1967; Carleton, 1973; Perrin & Curtis, 1980; Ellis et al., 1994; Langer, 2017; Barbero, Teta & Cassini, 2021). Although dietary variation has been reported within Ichthyomyini, all members rely on an animalivorous or carnivorous strategy, foraging for prey in freshwater habitats (*e.g*., Voss, 1988, 2015). Accordingly, a shared gastric pattern is expected, and all examined ichthyomyines possess a subglandular stomach (*e.g*., Carleton, 1973; Voss, 1988; Salazar-Bravo et al., 2023; Zeballos et al., 2025; Pacheco et al., 2025). Similar consistency occurs in other tribes with distinct dietary profiles. For example, in two small Andean tribal assemblages focused on specialized herbivory—although characterized by diversity in molar occlusal morphology (*e.g*., Hershkovitz, 1962; Pardiñas, Teta & Salazar-Bravo, 2015)—stomach patterns remain cohesive. Andinomyini (both genera equiglandular but with a distinctive “pocket” of cornified epithelium intruding into the antrum near the lesser curvature) and Euneomyini (all three genera supraglandular) illustrate this pattern. In this context, the presence of several stomach types within a tribe may reflect dietary diversification, a hypothesis that can be evaluated using available data.

Abrotrichini includes five genera and 16 species (Brito & Pardiñas, 2025), and their diets are moderately documented (*e.g*., Pearson, 1983, 1984; Meserve, Lang & Patterson, 1988; Perez Calvo, Maser & Maser, 1989; Silva, 2005). *Geoxus* and *Notiomys* specialize in invertebrates. In contrast, *Paynomys* consumes a substantial proportion of plant material, whereas *Abrothrix* can be considered omnivorous. Despite these dietary differences, all genera share the equiglandular stomach type (Teta et al., 2017; this article).

Akodontini includes 16 genera and 89 species (Brito & Pardiñas, 2025), and their diets have been studied using stomach contents and isotopic analyses (*e.g*., Pinotti, Naxara & Pardini, 2011; Missagia, Patterson & Perini, 2019; Pardiñas et al., 2020; Barbero, Teta & Cassini, 2021). Most akodontines are likely omnivorous, with a tendency toward grass and herb consumption in *Necromys*. *Oxymycterus* (Oxymycterina) is specialized in invertebrates, and *Blarinomys* (Scapteromyina) shows similar habits. Most Akodontina are equiglandular, whereas the other two subtribes contain a high frequency of diverticular stomachs. This condition is associated with soil-invertebrate feeders not only in sigmodontines but also in neotomines (*e.g*., *Onychomys*; Horner, Taylor & Padykula, 1965) and deomyines (*e.g*., *Lophuromys*; Genest-Villard, 1968). However, Scapteromyina also includes an equiglandular genus (*Bibimys*) and a discoglandular genus (*Scapteromys*).

In summary, when Akodontini are organized into subtribes, gastric variation is concentrated in Scapteromyina, whereas Akodontina and Oxymycterina are each characterized by a single predominant design. The presence of three distinct patterns within Scapteromyina may reflect dietary specialization, although available data remain limited. For example, *Kunsia*, the largest living sigmodontine, was originally described as a root-eater (Miranda Ribeiro, 1914). Isotopic analyses indicate elevated δ¹³C values, suggesting a tendency toward herbivory (Missagia, Patterson & Perini, 2019). This interpretation is supported by its pronounced hypsodonty (*e.g*., Hershkovitz, 1966; Pardiñas, D’Elía & Teta, 2009) and mandibular mechanics (Missagia et al., 2021). Notably, *Kunsia* possesses a diverticular stomach type also observed in invertebrate-feeding rodents. One possibility is that dietary diversification within Scapteromyina is independent of gastric morphology, as *Kunsia* shares the predominant stomach type of the subtribe. Alternatively, current knowledge of its diet may be incomplete. Observations of captive individuals indicate insect consumption (Bezerra et al., 2007). In addition, although hypsodonty is often associated with grass consumption, it may also relate to soil ingestion (*e.g*., Madden, 2015). Under this scenario, *Kunsia* could represent a permanent or seasonal myrmecophage (*sensu* Redford, 1987), consistent with its gastric morphology.

In summary, and recognizing that these observations resist simple interpretation, the available evidence remains insufficient to determine whether tribes with high gastric variability represent artefactual groupings or deviations from expected morphological conservatism.

### A refined but flexible stomach classification

Voss (1988) represents one of the earliest attempts to incorporate a gastric trait explicitly into a phylogenetic analysis. Following Carleton (1973), that study recognized two character states regarding the distribution of glandular epithelium in the stomachs of ichthyomyines. However, both conditions are included within a single category, subglandular, in the present framework.

Subsequent analyses that incorporated gastric characters into phylogenetic contexts also emphasized differences within certain sigmodontine tribes. Weksler (2006) scored oryzomyines according to whether the glandular epithelium was limited to the antrum or extended slightly into the *corpus*. Teta et al. (2017) applied the same distinction to abrotrichines. Pardiñas et al. (2020) introduced subvariants within stomach “types” to account for differences observed in some akodontine genera, such as *Bibimys* and *Necromys*. In all cases discussed above, these stomach designs are recognized here as belonging to the equiglandular category.

These contrasting interpretations do not diminish the importance of the differences highlighted by the studies cited above concerning the distribution of the glandular lining. Although stomach morphology should be treated with caution—often because it is evaluated from a single specimen—it is evident that not all organs assigned to the same category share identical morphology. This was noted by Carleton (1973: 15), who observed that the akodontine *Podoxymys* “…departs somewhat from the hemiglandular pattern. The bordering fold intersects the lesser curvature at a point midway between the incisura angularis and pylorus. Consequently, some cornified epithelium occupies the part of the antrum….” However, Carleton (1973) did not establish a distinct gastric category to accommodate *Podoxymys*.

Thus, intrinsic flexibility exists within the system, expressed to varying degrees depending on the category. This flexibility is minimal in the diverticular configuration: no major variation is observed or expected. Either the glandular epithelium is restricted to an external pouch, or it is not. The boundaries of this category—and therefore the identification of its members—are unambiguous. The discoglandular condition presents a similar situation; no other comparable distribution of glandular epithelium, restricted to the fundus along the greater curvature, has been identified.

In contrast, the remaining categories recognized here (*i.e*., subglandular, equiglandular, and supraglandular) exhibit some degree of flexibility. These broader limits accommodate observable variation, as acknowledged by Carleton (1973). Nearly all oryzomyines can be distinguished according to whether the glandular epithelium is restricted to the antrum or extends slightly into the *corpus* (*e.g*., Carleton, 1973; Carleton & Musser, 1989; Weksler, 2006; this study). These differences among genera may prove informative for refining taxonomic boundaries or intergeneric relationships (*e.g*., Patton & Hafner, 1983; Voss, Gómez-Laverde & Pacheco, 2002). However, when the objective is an intertribal assessment, such variants can be treated as intrinsic variation within a single category.

### Stomach convergences

The diverticular condition is among the most distinctive patterns in muroid stomach diversity. This stomach type has attracted attention since early studies (*e.g*., Tullberg, 1899) and has been repeatedly discussed in functional terms (*e.g*., Vorontsov, 1962, 1967; Echave Llanos & Vilchez, 1964; Carleton, 1973; Langer, 2017). Diverticular stomachs are present in two tribes of Sigmodontinae (Akodontini and Thomasomyini; Carleton, 1973; Voss, Gómez-Laverde & Pacheco, 2002), two tribes of Neotominae (Onychomyini and Peromyscini; Horner, Taylor & Padykula, 1965; Carleton, 1973; Kelly et al., 2023), and at least one genus in Deomyinae (*Lophuromys*; Genest-Villard, 1968) and one in Murinae (*Echiothrix*; Musser & Durden, 2002). This distribution supports the interpretation of the diverticular condition as a recurrent convergence within the muroid radiation, including multiple independent origins within Sigmodontinae.

Within Akodontini, diverticular stomachs occur in two genera of Oxymycterina (*Juscelinomys* and *Oxymycterus*) and four genera of Scapteromyina (*Blarinomys*, *Brucepattersonius*, *Kunsia*, and *Lenoxus*). In *Brucepattersonius* and *Oxymycterus*, all examined species possess this stomach type (two in the former and six in the latter; *e.g*., Tullberg, 1899; Echave Llanos & Vilchez, 1964; Vorontsov, 1967; Carleton, 1973; Hershkovitz, 1998; Pardiñas et al., 2020; Bezerra et al., 2025). All akodontine diverticular stomachs are highly similar. They are characterized by a protrusive *fornix ventricularis*, a cornified epithelium that is not markedly spongy but bears a faint median false bordering fold, and glandular pouches generally situated slightly to the right of the greater curvature. Communication between the pouch and the main lumen occurs through a minute diverticular orifice that consistently opens toward the left—*i.e*., toward the fundus of the *corpus* rather than the antrum.

*Aepeomys* represents the only non-akodontine diverticular example known within the sigmodontine radiation. Voss, Gómez-Laverde & Pacheco (2002: 15) first described its condition, noting that “gastric glandular epithelium in *Aepeomys* is restricted to a small, pouch-like structure on the greater curvature (closely resembling the condition illustrated by Carleton (1973: fig. 5C) for *Oxymycterus rutilans*).” Pacheco (2003: 105, fig. 10D), however, interpreted the stomach of this genus as discoglandular and provided a schematic drawing depicting a closed pouch. Examination of multiple specimens in the present study confirms that *Aepeomys* possesses a diverticular stomach. Some variability was observed in the attachment point of the pouch to the stomach wall (sometimes on the greater curvature, but more often on the right side of the antrum), but a major difference from the akodontine condition lies in its communication with the lumen. In *Aepeomys*, the pouch is perforated medially by a channel rather than by a minute orifice. In transverse section, the pouch has a subtriangular outline, further distinguishing it from akodontine examples. Its communication with the stomach cavity is directed toward the antrum rather than the *corpus*. This condition closely resembles that found in the North American genus *Onychomys*, a specialized neotomine predator of desert invertebrates (*e.g*., Horner, Taylor & Padykula, 1965; Kelly & Martin, 2025). In *Onychomys*, the pouch is likewise perforated medially and exhibits a subtriangular profile in sagittal section (Vorontsov, 1967, figs. 98 and 112).

In summary, these observations indicate that, although the diverticular condition is convergent between akodontines and thomasomyines, the two lineages differ in several anatomical details. A broader implication is that other potential convergences within sigmodontine gastric diversity warrant careful examination. For example, this study demonstrates the similarity between the stomachs of the *incertae sedis* genus *Chinchillula* and members of Andinomyini (Salazar-Bravo et al., 2016). A recent genomic analysis proposed recognizing the former as the sole member of a new tribe, Chinchillulini (Bangs et al., 2025). If this arrangement is supported, the stomachs of Andinomyini and Chinchillulini may represent a case of convergence. In this instance, convergence is detectable because, although equiglandular, these stomachs share a distinctive feature: an anterior, circumscribed intrusion of cornified epithelium into the antrum, bounded on the left by a false bordering fold (Salazar-Bravo et al., 2016; this study). Conversely, when equiglandular stomachs lack such distinctive features, convergence may remain undetected. It should be noted that phylogenetic relationships and ancestral-state reconstructions may vary depending on the dataset and tree topology used, and such variation may influence the inferred evolutionary patterns.

### Prospective anatomical expectations (and their taxonomic implications)

Based on patterns observed in related taxa, prospective expectations can be outlined for stomach morphology in genera that remain undescribed in this respect. In addition, working hypotheses relating stomach gross morphology to taxonomy can be proposed.

The akodontine *Gyldenstolpia* is among the least known sigmodontines. The genus was erected to accommodate a species previously assigned to *Kunsia* (*e.g*., Hershkovitz, 1966; Pardiñas, D’Elía & Teta, 2009). One might therefore expect its gastric morphology to align with that of *Kunsia*, *i.e*., the diverticular condition. However, a morphologically related genus, *Scapteromys*, exhibits a distinct discoglandular pattern (Carleton, 1973; Pardiñas et al., 2020). *Gyldenstolpia* has not yet been included in any molecular-based phylogeny, and its intergeneric relationships remain unresolved beyond its placement within Scapteromyina (Cañón, 2021). Within this subtribe, equiglandular, discoglandular, and diverticular stomachs are all represented. A discoglandular pattern in *Gyldenstolpia* would be consistent with a closer relationship to *Scapteromys* and would reinforce its distinctiveness relative to *Kunsia* (Pardiñas, D’Elía & Teta, 2009).

*Microakodontomys* is a small and distinctive terrestrial oryzomyine (*e.g*., Hershkovitz, 1993). Although its validity was questioned early on—at one point being treated as a junior synonym of *Oligoryzomys* in a table footnote (Weksler, Percequillo & Voss, 2006)—its generic distinctiveness has been progressively supported. A recent molecular result places it in close relationship with the giant amphibious *Lundomys* (Weksler et al., 2025). Most oryzomyines exhibit equiglandular stomachs, whereas *Holochilus*, a herbivorous genus, is supraglandular (and some species of *Nectomys* show a tendency toward this condition). Based on its molar morphology, a supraglandular tendency may be expected in *Microakodontomys*.

*Andalgalomys* and *Salinomys* are two rare phyllotines specialized in the consumption of halophytic plants (*e.g*., Williams & Mares, 1978; Braun & Mares, 1995). Although their intergeneric relationships remain unresolved, they appear closely related in both morphology and ancestry (*e.g*., Salazar-Bravo, Pardiñas & D’Elía, 2013; Carrizo & Catalano, 2015). All known Phyllotini exhibit equiglandular stomachs. The most parsimonious expectation is that *Andalgalomys* and *Salinomys* share this pattern.

*Aepeomys* is the only known non-akodontine example of a diverticular stomach. This genus is currently interpreted as part of Thomasomyini, following the first molecular analysis of the tribe (D’Elía et al., 2006a). However, its craniodental morphology does not align with the general pattern of the tribe (cf. Voss, Gómez-Laverde & Pacheco, 2002), a situation comparable to that of *Rhagomys*. The latter has been recognized as representing its own tribe, Rhagomyini (Pardiñas et al., 2022). The available evidence suggests that *Aepeomys* represents an ancient lineage—apparently monotypic—diverging from a basal thomasomyine-like stock. Given its gastric distinctiveness, *Aepeomys* may warrant recognition as a separate tribe.

Finally, *Chinchillula* exhibits a distinctive gastric morphology—although equiglandular—characterized by a “pocket” of cornified epithelium extending into the lesser curvature (*e.g*., Dorst, 1972; this study). This condition is also present in members of Andinomyini, *Andinomys* and *Punomys* (Salazar-Bravo et al., 2016). These genera also share several craniodental features and a common biogeographic pattern (*e.g*., Pearson, 1951; Patton, 2015). Although *Chinchillula* has recently been interpreted as the sole representative of Chinchillulini (Bangs et al., 2025), it may represent a basal member of Andinomyini.

### Gross stomach anatomy: limitations and further directions

Do equiglandular stomachs in abrotrichines, akodontines, oryzomyines, or phyllotines represent cases of convergence? Do they reflect a genuinely shared anatomical pattern, or do they exhibit broadly similar morphology while differing in subtle details, as observed for diverticular stomachs? These and related questions remain unresolved under the gross anatomical approaches applied thus far to sigmodontine stomachs. It is increasingly evident that this widely used methodology is insufficient and requires refinement (*e.g*., Pardiñas et al., 2020; Steiner et al., 2022). Histological studies—particularly those addressing the internal distribution and microanatomy of gastric glands—represent a direct path toward improving our understanding of the evolutionary diversification of gastric morphology. Although histology was routine in mammalogical research throughout much of the twentieth century (*e.g*., Blank, 1950; Golley, 1960; Echave Llanos & Vilchez, 1964; Genest-Villard, 1968; Dearden, 1969; Maggese, 1969; Lee et al., 1982; Perrin, 1986; Perrin & Kokkinn, 1986), it has largely disappeared from the current paradigm of “integrative taxonomy” in muroid rodents, which is now dominated by genetic data.

**Figure 25:**
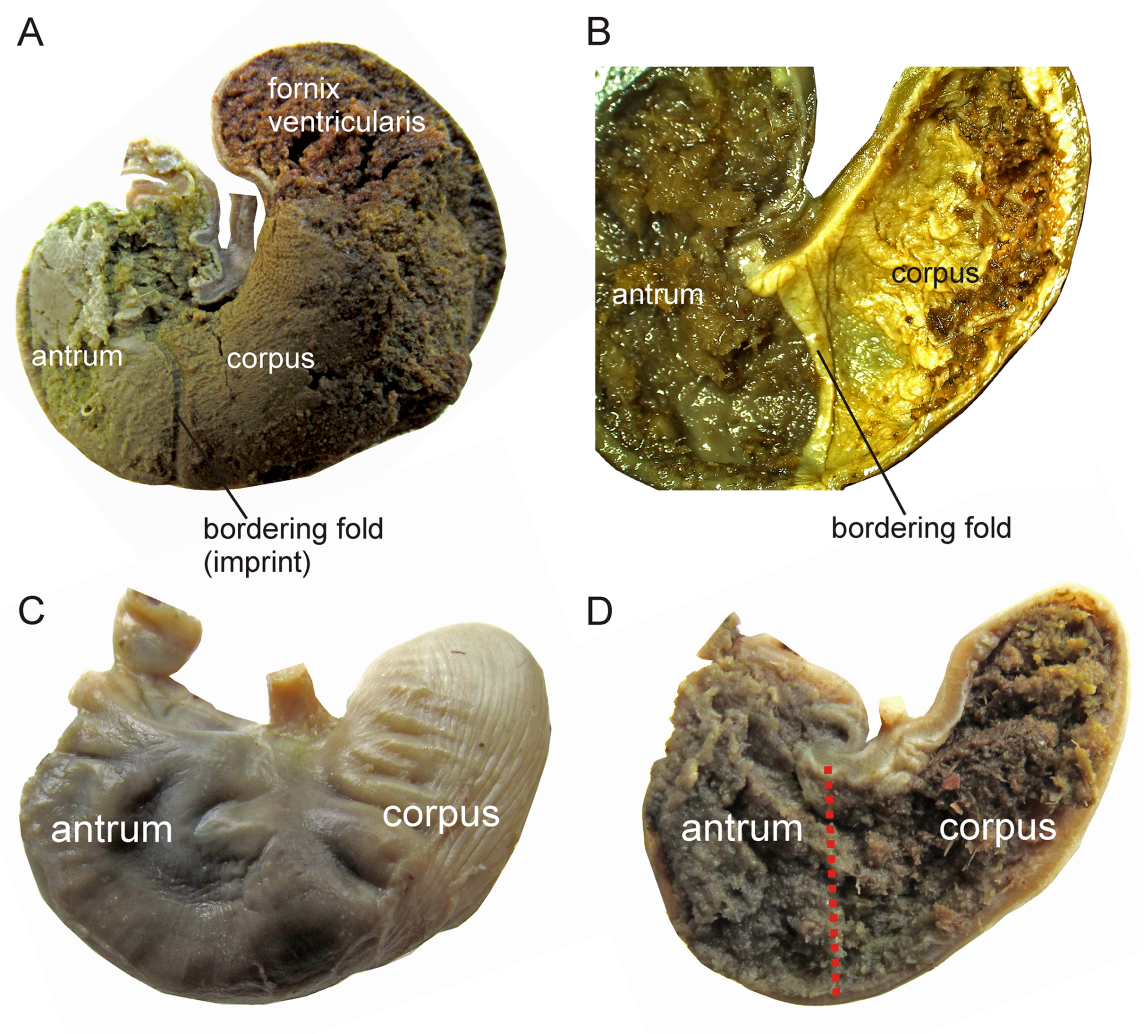
Sigmodontinae stomachs filled with food. Ventral views (A, B, D) and an external view (C): *Graomys chacoensis* (A; CNP 6632), *Loxodontomys micropus* (B; CNP-D 227), and *Irenomys tarsalis* (C, D; CNP-D 214). All figures are shown at approximately the same scale. Photographs by U. Pardiñas.

The present study indicates that the bordering fold warrants particular attention. Largely overlooked in previous works (*e.g*., Vorontsov, 1967; Carleton, 1973; Pardiñas et al., 2020), its parietal configuration and transverse structure suggest that it is more than a “pronounced ridge that marks the juncture of the two types of gastric mucosa” (Carleton, 1973: 10). Luciano & Reale (1992: 137) noted that the bordering fold “has various and more complex functions, as suggested by its architecture and by the brush cells which are particularly numerous in this region.” The morphology of the bordering fold differs among major sigmodontine tribes. Whereas abrotrichines, akodontines, and several smaller tribes (*e.g*., ichthyomyines, neomicroxines) exhibit relatively simple folds, the condition in phyllotines and thomasomyines is markedly different. Luciano & Reale (1992) proposed that the bordering fold may regulate the movement of fluid contents between the two principal stomach compartments. Bisected stomachs filled with food frequently show a differential distribution of contents demarcated by this fold (Fig. 25). This separation, which occurs even in unilocular stomachs, indicates that functional “barriers” influence alimentary processing (Perrin, 1986: 233).

Another aspect requiring closer examination is the texture of the cornified epithelium, both among tribes and within individual stomachs. This includes variation between the *corpus* and the fornix ventricularis. Since Vorontsov (1967), it has been recognized that this epithelium exhibits a range of textural irregularities that serve as substrates for dense bacterial communities associated with food processing. A few studies of muroid rodents have investigated the taxonomic composition of these symbionts, with or without reference to anatomical or histological features of the underlying tissues (*e.g*., Maddock & Perrin, 1981; Perrin, 1986; Kohl et al., 2011, 2013, 2014; Shinohara et al., 2016), but virtually nothing is known for sigmodontines. Both macroscopic and microscopic aspects appear relevant. For example, the cornified lining of the *corpus* and antrum in *Scapteromys* is macroscopically distinctive, with sets of rugae diverging in opposite directions (Pardiñas et al., 2020; Fig. 7H). In *Andinomys*, the highly expandable *corpus*—crossed by multiple accordion-like folds—bears a finely textured, spongy cornified epithelium (Figs. 8A, 8E). Mechanical factors likely play a major role, as this portion of the stomach undergoes extensive expansion during feeding. How these features vary within and among tribes remains an open question.

The anatomy of the region associated with the esophageal opening, the lesser curvature, and the pars pylorica also merits detailed study. Although relatively simple in several tribes (*e.g*., Abrotrichini, Akodontini, Euneomyini), it differs markedly in others (*e.g*., Ichthyomyini, some Phyllotini and Thomasomyini). Depending on the taxon, folds and channels characterize this region regardless of the epithelia involved. Langer (1984) described these groove-like structures across various mammals and discussed their functional implications (see also Dearden, 1966). Studies on African murids have documented the presence of complex esophageal structures—including grooves and valves—that enable alternate routes of digesta flow and facilitate regurgitation (*e.g*., Perrin & Curtis, 1980; Perrin, 1986; Perrin & Kokkinn, 1986). One of these functions may relate to the duration of lactation and the timing of weaning—critical aspects explored in only a few sigmodontine species of zoonotic or laboratory relevance (*e.g*., De Villafañe, 1981; Piantanida & Nani, 1993). Whether these structures in ichthyomyines are linked to precocial development, for example by facilitating rapid milk passage and enhancing stomach expansibility (cf. Genest-Villard, 1968: 654), remains unresolved.

In light of these considerations, it is noteworthy that approximately 10% of sigmodontine genera remain unsampled with respect to digestive anatomy. The gap is even greater at the species level: among the roughly 500 described species in the subfamily (Brito & Pardiñas, 2025), stomach morphology has been characterized for only about 30%.

As in other areas of soft-anatomy research (*e.g*., rhinaria; see Pardiñas et al., 2024), it is clear that although curatorial practices are improving (*e.g*., Timm, McLaren & Genoways, 2021), they still require further development. The preservation of specimens in fluid, allowing diverse anatomical investigations, should be encouraged. Despite recent field collections (*e.g*., Bazán-León et al., 2016; Uturunco & Pacheco, 2016), sigmodontine genera such as *Chelemys* and *Eremoryzomys* remain unstudied with respect to the digestive system. New specimens of the rare oryzomyine *Microakodontomys* have provided valuable phylogenetic insights (Weksler et al., 2025), but were preserved only as skins and skulls (J. Prado, email to UFJP, November 2025), precluding any assessment of gastric morphology.

Over the past decade, 58 new species of American cricetids have been described, yet fewer than 20% include information on stomach morphology (Table 6). This situation raises questions about the scope of integrative taxonomy currently practiced and how the “Vorontsov shortfall”—the persistent lack of basic anatomical data—is being addressed. A survey of Muridae reveals similarly limited anatomical knowledge beyond craniodental and external characters. Despite the contributions of researchers such as Guy Musser (*e.g*., Musser & Durden, 2002), recent murid descriptions often rely on a restricted set of traditional morphological traits. A recurring inconsistency is that stomach contents are sometimes reported, whereas stomach morphology itself is omitted (*e.g*., Esselstyn et al., 2015; Rowe, Achmadi & Esselstyn, 2014, 2016). These limitations partly reflect the nature of available material, particularly older specimens represented only by skins and skulls. However, many recent descriptions are based on newly collected specimens obtained through substantial field effort (*e.g*., Heaney et al., 2009). Predictions such as those proposed by Vorontsov (1967)—for example, the hypothesis of a diverticular stomach in Sulawesi’s carnivorous rodents—remain untested despite the availability of new material (*e.g*., Rickart et al., 2019). In summary, the scientific potential of collected specimens is often not fully realized, and the bioethical implications of specimen collection warrant careful consideration.

**Table 6:** List of American (non-arvicoline) cricetid species described since 2020 (total = 58), with indication of the presence or absence of published stomach morphology descriptions.

Species	Stomach description?
*Akodon diauarum* Brandão, Carmignotto, Percequillo, Christoff, Mendes-Oliveira & Geise, 2022	No
*Akodon kadiweu* Brandão, Percequillo, D’Elía, Paresque & Carmignotto, 2021	No
*Chilomys carapazi* Brito & Pardiñas, 2022	No
*Chilomys georgeledecii* Brito, Tinoco, García, Koch & Pardiñas, 2022	Yes
*Chilomys neisi* Brito, Tinoco, García, Koch & Pardiñas, 2022	No
*Chilomys percequilloi* Brito, Tinoco, García & Pardiñas, 2022	Yes
*Chilomys weksleri* Brito, García, Pinto & Pardiñas, 2022	No
*Daptomys nunashae* Pacheco, Sánchez-Vendizú, Fajardo, Cossíos & Cadenillas, 2025	Yes
*Euryoryzomys cerqueirai* Percequillo & Weksler, 2023	No
*Geoxus lafkenche* Teta & D’Elía, 2020	No
*Holochilus oxe* Prado, Knowles & Percequillo, 2021	No
*Ichthyomys pinei* de Córdova, Nivelo-Villavicencio, Reyes-Puig, Pardiñas & Brito, 2020	Yes
*Incanomys mayopuma* Zeballos, Pari, Medina & Pino, 2025	Yes
*Mindomys kutuku* Brito, Koch, Tinoco & Pardiñas, 2022	No
*Neacomys aletheia* Semedo, Silva, Carmignotto & Rossi, 2021	No
*Neacomys auriventer* Brito, Tinoco, Burneo, Koch, Arguero, Vargas & Pinto, 2021	Yes
*Neacomys elieceri* Semedo, Silva, Carmignotto & Rossi, 2021	No
*Neacomys jau* Semedo, Silva, Carmignotto & Rossi, 2021	No
*Neacomys leilae* Caccavo & Weksler, 2021	No
*Neacomys marajoara* Semedo, Silva, Gutiérrez, Ferreira, Nunes, Mendes-Oliveira, Farias & Rossi, 2020	No
*Neacomys marci* Brito & Tinoco, 2023	Yes
*Neacomys oliveirai* Caccavo & Weksler, 2021	No
*Neacomys serranensis* Colmenares-Pinzón, 2021	No
*Neacomys vossi* Semedo, Silva, Gutiérrez, Ferreira, Nunes, Mendes-Oliveira, Farias & Rossi, 2020	No
*Neacomys xingu* Semedo, Silva, Gutiérrez, Ferreira, Nunes, Mendes-Oliveira, Farias & Rossi, 2020	No
*Nephelomys ricardopalmai* Ruelas, Pacheco, Inche & Tinoco, 2021	No
*Oecomys galvez* Voss, Fleck & Giarla, 2024	No
*Oecomys hiceae* Voss, Giarla, Lim & Engstrom, 2025	No
*Oecomys jamari* Saldanha, Semedo, Mendonça, Lima-Silva, Messias, Sampaio, Brandão & Rossi, 2023	No
*Oecomys makampi* Voss, Fleck & Giarla, 2024	No
*Oecomys matogrossensis* Saldanha & Rossi, 2021	No
*Oecomys nanus* Voss, Fleck & Giarla, 2024	No
*Oligoryzomys gri* Bonvicino & Weksler, 2024	No
*Oligoryzomys guille* Hurtado, 2021	No
*Oreoryzomys huancabambensis* Llancachahua-Tarqui, Ruelas, Escobar & Pacheco, 2025	No
*Oreoryzomys jumandi* Brito, Vargas, García, Tinoco & Pardiñas 2025 in Brito et al. (2025)	No
*Oxymycterus willkaurco* Zeballos, Medina, Rico-Cernohorska & Salazar-Bravo, 2021	No
*Pattonimus ecominga* Brito, Koch, Percequillo, Tinoco, Weksler, Pinto & Pardiñas, 2020	Yes
*Pattonimus musseri* Brito, Koch, Percequillo, Tinoco, Weksler, Pinto & Pardiñas, 2020	No
*Peromyscus ensinki* Bradley, Ordóñez-Garza, Thompson, Wright, Ceballos, Kilpatrick & Schmidly, 2022	No
*Peromyscus greenbaumi* Bradley, Ordóñez-Garza, Thompson, Wright, Ceballos, Kilpatrick & Schmidly, 2022	No
*Peromyscus purepechus* Léon-Tapia, Fernández, Rico, Cervantes & Espinosa de los Monteros, 2020	No
*Phyllotis camiari* Teta, Jayat, Steppan, Ojeda, Ortiz, Novillo, Lanzone & Ojeda, 2022	No
*Phyllotis pehuenche* Jayat, Teta, Ojeda, Steppan, Osland, Ortiz, Novillo, Lanzone & Ojeda, 2022	No
*Rhagomys jequitiba* Bonvicino, Pires, Lanes & Faria, 2024	No
*Rhagomys septentrionalis* Moreno Cárdenas, Tinoco, Albuja & Patterson, 2021	No
*Rhipidomys bezerrensis* Campos, Percequillo & Langguth in Campos, Percequillo, Miranda & Langguth, 2022	No
*Rhipidomys caracolensis* Campos, Percequillo & Langguth in Campos, Percequillo, Miranda & Langguth, 2022	No
*Rhipidomys ochoagrateroli* García, Almeida, Machado, Delgado-Jaramillo, Araujo-Reyes, Vásquez-Parra & Flórez, 2020	No
*Rhipidomys ybyrae* Lanes & Bonvicino, 2023	No
*Thomasomys antoniobracki* Ruelas & Pacheco, 2021	No
*Thomasomys burneoi* Lee, Tinoco & Brito, 2022	No
*Thomasomys igor* Brito, García, Castellanos, Gavilanes, Curay, Carrión-Olmedo, Reyes-Barriga, Guayasamin, Salazar-Bravo & Pinto, 2024	No
*Thomasomys lojapiuranus* Pacheco & Ruelas, 2023	Yes
*Thomasomys otavalo* Brito, García, Castellanos, Gavilanes, Curay, Carrión-Olmedo, Reyes-Barriga, Guayasamin, Salazar-Bravo & Pinto, 2024	Yes
*Thomasomys pagaibambensis* Pacheco & Ruelas, 2023	Yes
*Thomasomys pardignasi* Brito, Vaca-Puente, Koch & Tinoco, 2021	No
*Thomasomys shallqukucha* Pacheco & Ruelas, 2023	Yes

### A new classification: failure or success

Reig (1972, 1977) identified a fundamental conceptual and terminological inconsistency in the description of muroid occlusal molar patterns and proposed a new topographical system to address it. This proposal—essentially a novel classificatory framework—largely replaced the nomenclature dominant at the time (*e.g*., Hershkovitz, 1962) and, over the last five decades, has become a prevailing paradigm in dental morphology (*e.g*., Carleton & Musser, 1989; Brito et al., 2020). It therefore represents a clear example of a successful new classification. In contrast, the ICAMER system proposed by Barbière et al. (2019), conceived to redefine molar occlusal morphology, appears to have achieved limited adoption. Although more recent, this topological approach has not yet been widely applied (with the exception of Barbière et al., 2021). Several phylogenies incorporating dental characters continue to rely on traditional—sometimes even sui generis—classificatory systems (*e.g*., Dirnberger, Peláez-Campomanes & López-Antoñanzas, 2024). Within this context, the success or failure of a classificatory system can be assessed by the extent of its adoption within the scientific community.

A closer examination of Reig’s (1977) classification reveals a more nuanced situation. While widely adopted in South America—supported by influential contributions such as Carleton (1980) and Voss (1988)—other regions retained earlier descriptive traditions. In particular, the system proposed for muroid molars did not gain traction in the Old World. Africa, Asia, and especially Europe have continued to describe dentitions using earlier frameworks (*e.g*., Freudenthal, Hugueney & Moissenet, 1994; Lindsay, 1988; Marivaux, Vianey-Liaud & Jaeger, 2004; Lazzari et al., 2008; Rodrigues et al., 2011). This pattern suggests that provincialism, entrenched traditions, permissiveness in publication practices, and other factors can hinder the adoption of new classificatory systems.

A comparable dynamic can be observed in the development of gastric classifications. Carleton (1980) emphasized the need to refine his earlier scheme. Although this study was largely overlooked in subsequent analyses of American cricetid radiations, it contains the basis for an unrealized revision of stomach morphology. In that work, Carleton (1980: 58) assigned rodents to the categories hemiglandular, intermediate grade I, intermediate grade II, discoglandular, hemipouched, and fully pouched. Some of these designs correspond to those described previously (Carleton, 1973), including the hemiglandular and discoglandular types, and likely the “fully pouched” condition. However, the intermediate categories were not explained, and the proposed scheme was not subsequently adopted. It remained unacknowledged even in broad treatments of the mammalian gastrointestinal tract (*e.g*., Langer, 2017).

Instead, the earlier scheme of Carleton (1973) became the standard for describing stomach gross anatomy. Unlike the regional success of Reig’s (1977) dental system, however, this gastric classification achieved only moderate adoption beyond the Americas. A non-exhaustive survey of stomach descriptions indicates that the two key traits emphasized by Carleton (1973)—penetration of the *incisura angularis* and the relative distribution of glandular *vs*. cornified epithelium—have been applied in studies of murids and other muroid groups (*e.g*., Perrin & Curtis, 1980; Boonzaier et al., 2013; Walters et al., 2014). Nevertheless, adoption has not been consistent. Earlier or hybrid classifications persist, as reflected in the continued use of terms such as forestomach and limiting ridge (*e.g*., Byanet et al., 2010; Likidkarnchanakornkij, Jindatip & Wannaprasert, 2023). Carleton’s framework has also been largely absent from laboratory and biomedical studies, particularly those involving *Mus* and *Rattus* (*e.g*., Lee et al., 1982; Ghoshal & Bal, 1989).

Given the current landscape of sigmodontine research—characterized by multiple groups working on closely related topics, often in a competitive context, and not always engaging with alternative proposals (*e.g*., Barbero, Teta & Cassini, 2021)—the future adoption of the classification proposed here remains uncertain.

## Final considerations

About half a century ago, the influential work of Carleton (1973, 1981) promoted the use of a standardized English and Latin nomenclature to distinguish the principal components of the rodent stomach. This terminology was widely adopted in studies of American cricetids, but persistent terminological diversity continues to characterize part of the broader muroid literature, likely due to the influence of veterinary and laboratory-animal traditions that have largely overlooked glirological sources (*e.g*., Fox, 2015; Lossi et al., 2016; Chawla & Jena, 2021; Venkataraman & Raajkamal, 2021). The refined classification advanced here aims to address this situation with two principal goals: first, to reestablish a coherent framework that facilitates standardized and informative stomach descriptions; and second, to promote more effective scientific communication among researchers working on muroid rodents.

The comparative evidence assembled in this study indicates that sigmodontine gastric morphology is neither uniform nor chaotic, but structured across multiple hierarchical levels. The predominance of the equiglandular condition, supported as the ancestral state for Sigmodontinae and its sister clade, contrasts with derived and often lineage-specific deviations observed in several tribes. Some groups—such as Oryzomyini, Phyllotini, and Ichthyomyini—exhibit marked morphological conservatism, whereas others, notably Akodontini and Thomasomyini, display a broader range of anatomical configurations, including the diverticular condition. These contrasting patterns indicate that stomach morphology retains substantial phylogenetic signal while also documenting repeated, independent innovations.

The identification of cases of convergence—most clearly in the diverticular stomachs of akodontines and thomasomyines—further shows that similar ecological pressures can produce superficially similar configurations through distinct anatomical pathways. These findings underscore the importance of evaluating not only the overall distribution of glandular and cornified epithelia, but also the detailed architecture of structures such as the bordering fold, the fornix ventricularis, and the attachment and shape of glandular pouches. These features often reveal evolutionary patterns that are not evident from gross proportional relationships alone.

At the same time, this study highlights the limitations of relying exclusively on macroscopic observations. The internal distribution of glands, the histological structure of the bordering fold and associated regions of the lesser curvature, and the microscopic texture of the cornified epithelium remain insufficiently characterized across most sigmodontine lineages. These gaps hinder the evaluation of potential convergence among equiglandular stomachs and limit deeper functional and phylogenetic interpretations. Renewed investment in histological series, detailed anatomical documentation, and improved curatorial practices—particularly the preservation of specimens suitable for internal anatomical study—is therefore essential. Addressing this persistent “Vorontsov shortfall” will be necessary to fully realize the evolutionary and systematic value of digestive-system morphology.

## Supplemental Information

10.7717/peerj.21405/supp-1 Supplemental Information 1 Appendix S1. Exhaustive list of the stomachs studied for this contribution.

10.7717/peerj.21405/supp-2 Supplemental Information 2 GenBank accession numbers for all mitochondrial and nuclear DNA sequences analyzed in this study. Loci include cytochrome b (cytb), interphotoreceptor retinoid-binding protein (IRBP), growth hormone receptor (GHR), and recombination activating gene 1 (RAG1).

10.7717/peerj.21405/supp-3 Supplemental Information 3 Proportional likelihoods of stomach morphology types at ancestral nodes for each tribe. Values were estimated using maximum likelihood under the Mk1 model (equal transition rates). For each principal node, proportional likelihoods are reported for the five stomach types: discoglandular, diverticular, equiglandular, subglandular, and supraglandular.

10.7717/peerj.21405/supp-4 Supplemental Information 4 Stomach of Oxymycterus by Vorontsov. Original illustration of the stomach of *Oxymycterus rufus* from Vorontsov (1967: fig. 100).

10.7717/peerj.21405/supp-5 Supplemental Information 5 Stomach of Necromys obscurus. Necromys obscurus, internal view of the stomach (ventral aspect) based on specimen CNP 6035.

10.7717/peerj.21405/supp-6 Supplemental Information 6 Raw data - stomach states for ancestral reconstruction.

10.7717/peerj.21405/supp-7 Supplemental Information 7 Raw data - stomach tree reconstruction.

10.7717/peerj.21405/supp-8 Supplemental Information 8 Raw data - sequences used for Sigmodontinae phylogeny.

## Additional Information and Declarations

### Data Availability

The following information was supplied regarding data availability:

The raw data is available in the Supplemental Files.
