# Supplementary material for: Gastric morphology in sigmodontine rodents (Mammalia: Cricetidae): a comprehensive comparative classification
Source: PeerJ. 2026 Jun 10;14:e21405. doi: 10.7717/peerj.21405 (PMC13264281; doi:10.7717/peerj.21405)
Supplement: Supplemental Information 1 [file peerj-14-21405-s001.docx]

**Appendix S1. Exhaustive list of the stomachs studied for this contribution.**

Studied specimens (n = 602) belong to the following mammal collections:

CBF, Colección Boliviana de Fauna, La Paz, Bolivia;

CNP, Colección de Mamíferos del Centro Nacional Patagónico, Chubut, Argentina;

CNP-D, Colección de Mamíferos [anexo digestivos] del Centro Nacional Patagónico, Chubut, Argentina;

CML, Colección Mamíferos Lillo, Tucumán, Argentina;

CMUFLA, Colección de Mamíferos de la Universidade Federal de Lavras, Minas Gerais, Brazil;

CMZ, Colección Museo de Zoología “Alfonso Herrera”, Universidad Autónoma de México, Mexico;

CVULA, Colección de Vertebrados, Universidad de Los Andes, Mérida, Venezuela;

MECN, Colección de Mastozoología del Instituto Nacional de Biodiversidad (INABIO), Quito, Ecuador;

MN, Museu Nacional, Rio de Janeiro, Brazil;

MUSA: Museo de Historia Natural, Universidad Nacional de San Agustín, Arequipa, Peru;

MZUFV, Museu de Zoologia, Departamento de Biologia Animal, Universidade Federal de Viçosa, Viçosa, Minas Gerais, Brazil;

QCAZ, Museo de Zoología de la Pontificia Universidad Católica del Ecuador, Quito, Ecuador;

UFSC: Universidade Federal de Santa Catarina, Florianópolis, Santa Catarina, Brazil.

Specimens listed with numbers of collector or field catalogues, including CG (Carlos Galliari), JBM (Jorge Brito Molina), LTU (Proyecto Localidades Típicas Ulyses Pardiñas), RCC (Raisa Cairampoma Cotrado), UP (Ulyses Pardiñas), will be deposited in the following collections: CNP (those CG, LTU, and UP), MECN (those JBM).

Tribe Abrotrichini

Subtribe Abrotrichina

Genus *Abrothrix* (n = 22)

*Abrothrix gossei* (n = 2): Argentina, Mendoza, Uspallata (CNP 7089 [CNP-D 302]), Diamante River (CNP 8044 [CNP-D 306]).

*Abrothrix hirta* (n = 8): Argentina, Santa Cruz, Lago Strobel (CNP 4302 [CNP-D 7]), Chubut, Carhué Niyeu (PPA 286 [CNP-D 26]); Mendoza, Portezuelo del Viento (CNP 6348 [CNP-D 63]); Santa Cruz, Estancia La Ensenada (CNP 2712 [CNP-D 285], CNP 2715 [CNP-D 287]), CNP 2724 [CNP-D 289]); Chubut, Lago Fontana, 1 km E RP 57 desde cabaña municipal (CNP 5438 [CNP-D 406], CNP 4808 [CNP-D 408]).

*Abrothrix lanosa* (n = 2): Argentina, Tierra del Fuego, Ushuaia, CADIC (CNP 1389 [CNP-D 2], CNP 1393 [CNP-D 19]).

*Abrothrix olivacea* (n = 10): Argentina, Tierra del Fuego, Ea. San Julio, Río Grande (LTU 454 [CNP-D 4]); Mendoza, Portezuelo del Viento (CNP 6355 [CNP-D 74]); Río Negro, Bariloche (CNP 6663 [CNP-D 195], CNP 6662 [CNP-D 196], CNP 6664 [CNP-D 197], CNP 6661 [CNP-D 203]); Neuquén, Villa Litrán (CNP 7691 [CNP-D 529]); Chubut, Cholila (CNP 8584 [CNP-D 543], CNP 8585 [CNP-D 542]). Bolivia, Potosí, Khastor (CBF 11989).

Subtribe Notiomyina

*Geoxus* (n = 3)

*Geoxus valdivianus* (n = 3): Argentina, Neuquén, Villa Litrán (CNP 6280 [CNP-D 34], Riscos Negros (CNP 7624 [CNP-D 508], CNP 6281 [CNP-D 538]).

*Notiomys* (n = 2)

*Notiomys edwardsii* (n = 2): Argentina, Río Negro, Altiplanicie del Somuncurá (CNP 1 [CNP-D 30]); Chubut, Estancia El Huetel (CNP 6286 [CNP-D 36]).

*Paynomys* (n = 8)

*Paynomys macronyx* (n = 8): Argentina, Santa Cruz, Estancia Tucu-Tucu (CNP 3887 [CNP-D 1], CNP 3889 [CNP-D 53]); Neuquén, Mirador del Arroyo Pil-pil (CNP 436 [CNP-D 321], CNP 440 [CNP-D 437]), laguna Varvarco Tapia (CNP 442 [CNP-D 21], Villa La Angostura (CNP 3630 [CNP-D 275]); Chubut, Cañadón de la Madera (CNP 2375 [CNP-D 6], CNP 7077 [CNP-D 25]).

Tribe Akodontini

Subtribe Akodontina

Genus *Akodon* (n = 111)

*Akodon aerosus* (n = 8): Ecuador, Morona Santiago, Kutukú (MECN 5820, MECN 5796, MECN 5861); Tungurahua, Baños, Reserva Vizcaya (MECN 6109, MECN 6110); Zamora Chinchipe, Machinaza (MECN 6129). Bolivia, La Paz, Cargadero (CBF 9835, CBF 9851).

*Akodon albiventer* (n = 4): Argentina, Jujuy, Sierra de Zenta (CNP 5490, CNP 5075, CNP 6062, CNP 6063).

*Akodon azarae* (n = 8): Argentina, Buenos Aires, Arroyo de las Brusquitas (UP 349), Laguna Chascomús (CNP 6582, CNP 6701, CNP 6824), Arroyo El Pescado (CNP 7604 [CNP-D 263]), Calle 143 y Diagonal 630 (CNP 7546 [CNP-D 535]), EEA INTA Hilario Ascasubi, linde norte (CNP 3115 [CNP-D 419]); Formosa, Estación de Animales Silvestres Guaycolec, Ruta Nacional 11, km 1201 (CG 447).

*Akodon budini* (n = 3): Argentina, Jujuy, El Matadero, 26.2 km SE Tilcara (CML 11110, CML 11113); Salta, El Queñoal, 54 km O San Andrés (CML 11118).

*Akodon cursor* (n = 1): Brasil, Minal Gerais, Mata do Paraíso (MZUFV 4481).

*Akodon dayi* (n = 1): Bolivia, La Paz, Mamacona (CBF 9831).

*Akodon dolores* (n = 15): Argentina, Catamarca, Camino Trampasacha-Chumbicha (CNP 3871); Córdoba, Deán Funes (CNP 430, CNP 4730); Parque Provincial Chancani (CNP 4731, CNP 4732), Quilino (CNP 4891); Neuquén, 1 km aguas abajo puente RN 40 sobre río Neuquén (CNP 2381), calle América del Sur, 200 m toma de agua, Neuquén (CNP 2346, CNP 2351), Rincón de los Sauces (CNP 6676 [CNP-D 252]); Chaco, río de Oro, a 300 m río arriba del puente que atraviesa la RP 33 (CNP 5275); Mendoza, Diamante River (CNP 7091 [CNP-D 531]), Estación Transformadora 25 de Mayo (CNP 7655 [CNP-D 308]), Río Negro, Est. El Cóndor, 500 m tranquera, boca del río Negro (CNP 3861 [CNP-D 457]); Buenos Aires, Laguna Chasicó (CNP 3857 [CNP-D 463]).

*Akodon iniscatus* (n = 11): Argentina, Chubut, Playa Fracasso (CNP 1408, CNP 1440, CNP 1458, CNP 5177 [CNP-D 100]), Playa Doradillo (CNP 1411), Bahía Crácker (CNP 1421, CNP 1438, CNP 5176 [CNP-D 59]), Los Altares (CNP 1417, CNP 5175 [CNP-D 55]), Parque Eólico “Malaspina” (CNP 6410 [CNP-D 18]).

*Akodon mollis* (n = 17): Ecuador, Pichincha, Reserva Geobotánica Pululahua (MECN 5201, MECN 5202), Quito, Volcán Pichincha, en las antenas (QCAZ 2644); El Oro, cuenca alta del río Santa Rosa (MECN 5767, MECN 5765, MECN 5759, MECN 5760, MECN 5758, MECN 5755, MECN 5766); Morona Santiago, Parque Nacional Sangay (MECN 4367, MECN 4369, MECN 5650, MECN 5673, MECN 5676, MECN 5679); Tungurahua, Pondoa (MECN 6074).

*Akodon montensis* (n = 25): Argentina, Chaco, 7 km NW Puerto Las Palmas (CNP 3093); Misiones, Parque Provincial Piñalito (CG 776 [CNP-D 115], CG 797 [CNP-D 88], CG 806 [CNP-D 118], CG 819); Club de Pesca Parana-í Guazú (CNP 4007, CNP 3051), Parque Provincial Urugua-í (CNP 3703), Parque Provincial Cruce Caballero, sendero Carayá Pytha (CNP 3030), Parque Provincial Moconá, sendero Chachí (CNP 3004), Refugio Moconá (CNP 4003, CNP 4004, CNP 4013, CNP 4011, CNP 4012) Reserva de Usos Múltiples Guaraní (UP 983 [CNP-D 113], UP 1006 [CNP-D 104], CNP 4015, CNP 4006, CNP 4010, CNP 4002, CNP 4008, CNP 4025), Reserva de Vida Silvestre Urugua-í, Fundación Vida Silvestre (CNP 3707, CNP 3716).

*Akodon oenos* (n = 9): Argentina, Mendoza, 10 km SSE Bardas Blancas (CNP 6334, UP 4053 [CNP-D 3]); río Atuel, margen izquierda, 500 m aguas abajo puente RP 175 (CNP 5311, CNP 5312); Diamante River (UP 4146 [CNP-D 309], UP 4154 [CNP-D 306], UP 4153 [CNP-D 307]); San Juan, Parque Nacional San Guillermo (CNP 3244, CNP 3245).

*Akodon philipmyersi* (n = 4): Argentina, Misiones, Estancia Santa Inés (CNP 3013, CNP 3019, CNP 3020, CNP 3021).

*Akodon polopi* (n = 3): Argentina, Córdoba, Pampa de Achala, Repetidora La Posta (CNP 5162, CNP 5163, CNP 5164).

*Akodon simulator* (n = 2): Argentina, Jujuy, El Ceibal (CNP 1532, CNP 1533).

Genus *Castoria* (n = 3)

*Castoria angustidens* (n = 3): Argentina, Misiones, RP2, 6 km NE arroyo Paraíso (CNP 449 [CNP-D 10]). Brasil, Paraná, Mananciais da Serra (MN 78417, MN 78460).

Genus *Deltamys* (n = 8)

*Deltamys kempi* (n = 8): Argentina, Buenos Aires, La Balandra, Club de Pesca La Terraza (CNP 581, CNP 893, CNP 3086, CNP 3087, CNP 5756 [CNP-D 45], CNP 6713 [CNP-D 97]), Ciudad Autónoma de Buenos Aires, Reserva Ecológica Costanera Sur (CNP 6295, CNP 6297 [CNP-D 33]).

Genus *Microxus* (n = 12)

*Microxus mimus* (n = 12): Bolivia, La Paz, Isañuyoj (CBF 9880, CBF 9886, CBF 9900, CBF 9895, CBF 9903, CBF 9922), Puina (CBF 9457, CBF 9490), Chullo (CBF 10162, CBF 10165, CBF 10202, CBF 10216).

Genus *Necromys* (n = 34)

*Necromys amoenus* (n = 2): La Paz, Machariapo (CBF 9353, CBF 9370).

*Necromys lactens* (n = 4): Argentina, Jujuy, San Francisco (CNP 4124), río Lozano, 3 km aguas arriba RN 9, León (CNP 6040); Salta, Abra de Volcán, 38.8 km ENE Humahuaca (CML 11483), El Queñoal, 54 km O San Andrés (CML 11485).

*Necromys lasiurus benefatus* (n = 6): Argentina, Buenos Aires, 5 km N Monte Hermoso (CNP 5244 [CNP-D 44]); Córdoba, Deán Funes (CNP 4727, CNP 4780, CNP 432, CNP 694, CNP 698).

*Necromys lasiurus liciae* (n = 3): Argentina, Chaco, 5 km NW Puerto Las Palmas (CNP 3036), Parque Nacional El Impenetrable (CNP 6634 [CNP-D 182]); Formosa, Estación de Animales Silvestres Guaycolec, Ruta Nacional 11, km 1201 (CNP 5011).

*Necromys lasiurus temchuki* (n = 6): Argentina, Misiones, Estancia Santa Inés (CNP 3041, CNP 3043, CNP 8287, CNP 7155, CNP 6576 [CNP-D 66]), EEA INTA Villa Miguel Lanús (CNP 3042).

*Necromys lasiurus*? (n = 1): Bolivia, La Paz, Pampas de Heath (CBF 9954).

*Necromys obscurus* (n = 11): Argentina, Buenos Aires, Estación San José (CNP 6030, CNP 6034 [CNP-D 249], CNP 6035), Arroyo de las Brusquitas (CNP 2380, CNP 3039, CNP 3055, CNP 3056), Arroyo El Pescado (CNP 7156 [CNP-D 547], CNP 7675 [CNP-D 270], CNP 8576 [CNP-D 273), UP 5002 [CNP-D 265]).

*Necromys urichi* (n = 1): Venezuela, exact locality not recorded (CNP-D 84).

Genus *Thalpomys* (n = 4)

*Thalpomys cerradensis* (n = 2): Brasil, Distrito Federal, Parque Nacional de Brasília (MN 75700, MN 75703).

*Thalpomys lasiotis* (n = 2): Brasil, precise locality not recorded, bioterio (MN 75695, MN 75704).

Genus *Thaptomys* (n = 19)

*Thaptomys nigrita* (n = 19): Argentina, Misiones, 2 km aguas abajo desembocadura Parana-í Guazú (CNP 3009), Asentamiento aborigen Kaaguy Poty, 1 km al NNO de la intersección de la Ruta Provincial 7 y el arroyo Cuña Pirú (CNP 1970 [CNP-D 92], CNP 2370, CNP 2371, CNP 2372), Parque Provincial Piñalito (CNP 7812, CNP 6755, CNP 7334, CNP 7338, CNP 7812 [CNP-D 125], CNP 7813 [CNP-D 127], CNP 7814 [CNP-D 119], CNP 7815 [CNP-D 123]), Parque Provincial Urugua-í (CNP 4262), Refugio Moconá (CNP 3011 [CNP-D 78], CNP 3008, LTU 859 [CNP-D 51], CNP 7203 [CNP-D 93]), Reserva de Vida Silvestre Urugua-í, Fundación Vida Silvestre (CNP 3875 [CNP-D 27]).

Subtribe Oxymycterina

Genus *Juscelinomys* (n = 2)

*Juscelinomys* cf. *J. huanchacae* (n = 2): Bolivia, Beni, Lago Guachuna (CBF 12041, CBF 12045).

Genus *Oxymycterus* (n = 31)

*Oxymycterus akodontius* (n = 3): Argentina, Jujuy, San Francisco (CNP 6298, CNP 6301), 3 km aguas arriba río Lozano (CNP 6299).

*Oxymycterus hiska* (n = 1): Bolivia, La Paz, Machariapo (CBF 9341).

*Oxymycterus inca* (n = 1): Bolivia, La Paz, Machariapo (CBF 9355).

*Oxymycterus quaestor* (n = 3): Argentina, Misiones, Parque Provincial Piñalito (CG 785, CNP 6603 [CNP-D 128]). Brasil, Santa Catarina, UHE Quebra Queixo, municipality of Ipuaçu (UFSC 6030).

*Oxymycterus rufus* (n = 20): Argentina, Buenos Aires, Estación San José (CNP 6307 [CNP-D 35], CNP 6308 [CNP-D 39]), Calle 143 y Diagonal 630, Arana, La Plata (CNP 7540 D548), Entre Ríos, Ruta Nacional 130, km 9, Villa Elisa, Colón (CNP 6568, CNP 7304), Est. Experimental Olmos (CNP 7571 [CNP-D D65]), Arroyo El Pescado (CNP 6932 [CNP-D 257], CNP 8596 [CNP-D 258], CNP 6929 [CNP-D 261], CNP 8034 [CNP-D 268], CNP 8033 [CNP-D 269], CNP 6933 [CNP-D 271], CNP 8614 [CNP-D 274], CNP 6931 [CNP-D 281], CNP 8608 [CNP-D 545]), Arroyo Sauce Grande (CNP 4946 [CNP-D 330], CNP 4956 [CNP-D 506]), Campamento Base, Sierra de la Ventana (CNP 4948 [CNP-D 461], CNP 4953 [CNP-D 428]). Brasil, Minas Gerais, Mata do Paraíso (ARA 353).

*Oxymycterus willkaurko* (n = 3): Bolivia, La Paz, Isañuyoj (CBF 9891), Puina (CBF 9480, CBF 9481).

Subtribe Scapteromyina

Genus *Bibimys* (n = 8)

*Bibimys chacoensis* (n = 2): Argentina, Chaco, 7 km S Puerto Las Palmas (CNP 1891 [CNP-D 9]), Cancha Larga (CNP 756).

*Bibimys labiosus* (n = 6): Brasil, Minas Gerais, Mata do Paraíso (CNP 8652 [CNP-D 13], MN 62062, MN 62063, MZUFV 4465, MZUFV 4470, MZUFV 4469).

Genus *Blarinomys* (n = 23)

*Blarinomys breviceps* (n = 23): Brasil, Minas Gerais, Mata do Paraíso (ARA 045 [CNP-D 57], MZUFV 4443, MZUFV 4444, MZUFV 4445, MZUFV 4446, MZUFV 4447, MZUFV 4448, MZUFV 4449, MZUFV 4450, MZUFV 4451, MZUFV 4452, MZUFV 4453, MZUFV 4454, MZUFV 4455, MZUFV 4456, MZUFV 4457, MZUFV 4458, MZUFV 4459, MZUFV 4460, MZUFV 4461, MZUFV 4462, MZUFV 4463, MZUFV 4464).

Genus *Brucepattersonius* (n = 7)

*Brucepattersonius iheringi* (n = 7): Argentina, Misiones, Parque Provincial Urugua-í (CNP 5507 [CNP-D 12]), RP2, 6 km NE arroyo Paraíso (CNP 1933 [CNP-D 79]), Salto El Paraíso (CNP 1932 [CNP-D 11]), Refugio Moconá (CNP 1999 [CNP-D 16]), Alrededores del asentamiento aborigen Kaaguy Poty, 1 km al NNO de la intersección de la Ruta Provincial 7 y el arroyo Cuña Pirú (CNP 1973 [CNP-D 96]), Parque Provincial Moconá, sendero de la Gruta (CNP 2331), Arroyo Liso (CNP 2368).

Genus *Kunsia* (n = 1)

*Kunsia tomentosus* (n = 1): Brasil, Goiás, Parque Nacional das Emas (MN 62569).

Genus *Lenoxus* (n = 5)

*Lenoxus apicalis* (n = 5): Bolivia, La Paz, Cargadero (CBF 9859, CBF 9877), Sarayoj (CBF 10085, CBF 10124, CBF 10080).

Genus *Scapteromys* (n = 17)

*Scapteromys aquaticus* (n = 17): Argentina, Buenos Aires, La Balandra, Club de Pesca La Terraza (CNP 6404 [CNP-D 40], CNP 6405, CNP 6406, CNP 6407, CNP 6408, CNP 6409, CNP 6622 [CNP-D 117], CNP 6665 [CNP-D 244], CNP 6666 [CNP-D 255], CNP 6586 [CNP-D 114], CNP 6989 [CNP-D 544], CNP 7541 [CNP-D 112]); Corrientes, Ea. Loma Alta, La Cruz (CNP 6307); Entre Ríos, Ruta Nacional 130, km 9, Villa Elisa (CNP 6308, CNP 6605 [CNP-D 107]); Formosa, Estación de Animales Silvestres Guaycolec, Ruta Nacional 11, km 1201 (CNP 5067), Corrientes, Ea. Loma Alta, La Cruz (CNP 8597 [CNP-D 109]).

Tribe Andinomyini

Genus *Andinomys* (n = 2)

*Andinomys edax* (n = 2): Argentina, Jujuy, Pucará, Humahuaca (CNP 5401 [CNP-D 243]), camino a Garganta del Diablo, Tilcara (CNP 5491 [CNP-D 242]).

Genus *Punomys* (n = 1)

*Punomys* cf. *P. kofordi* (n = 1): Peru, Puno, Hacienda Aricoma, Sandia (MUSA 4692).

Tribe Euneomyini

Genus *Euneomys* (n = 6)

*Euneomys chinchilloides* (n = 6): Argentina, Mendoza, Parque Provincial Aconcagua (CNP 8581 [CNP-D 8]); Río Negro, Altiplanicie del Somuncurá, Laguna Blanca (CNP 7187 [CNP-D 541], CNP 7188 [CNP-D 250]); Santa Cruz, Estancia La Ensenada (CNP 7092 [CNP-D 291]), Estancia Librun (CNP 6529 [CNP-D 140]), Meseta del Strobel (CNP 7190 [CNP-D 515]).

Genus *Irenomys* (n = 12)

*Irenomys tarsalis* (n = 12): Argentina, Neuquén, inmediaciones Lago Traful (MNT 154 [CNP-D 222], MNT 161 [CNP-D 226], MNT 163 [CNP-D 221], MNT 164 [CNP-D 216], MNT 165 [CNP-D 214], MNT 167 [CNP-D 228], MNT 169 [CNP-D 229], MNT 172 [CNP-D 213], MNT 173 [CNP-D 210]); Santa Cruz, Lago Fontana, inmediaciones cabaña municipal (CNP 5295 [CNP-D 388], CNP 5424 [CNP-D 404], CNP 7181 [CNP-D 46]).

Genus *Neotomys* (n = 4)

*Neotomys ebriosus* (n = 4): Argentina, Salta, San Antonio de los Cobres (CNP 3639 [CNP-D 48], CNP 3640 [CNP-D 237]). Peru, Áncash, Huari, San Marcos (RCC 473), Tacna, Candarave (RCC 479).

Tribe Ichthyomyini

Subtribe Ichthyomyina

Genus *Daptomys* (n = 3)

*Daptomys peruviensis* (n = 3): Ecuador, Pastaza, Piatúa (MECN 8072); Morona Santiago, Chalwayaku (MECN 7175). Peru, Coronel Portillo, Iparía, río Shesha (MUSA 12657).

Genus *Ichthyomys* (n = 10)

*Ichthyomys hydrobates* (n = 3): Ecuador, Cotopaxi, San Franscisco de las Pampas (QCAZ 818). Venezuela, Mérida, río Mocoties, via a Tovar (CVULA 1291), río Limones, 7 Km NE La Azulita (CVULA 7821).

*Ichthyomys orientalis* (n = 1): Ecuador, Tungurahua, Baños (MECN 6370).

*Ichthyomys pinei* (n = 1): Ecuador, Azuay, Nabón (MECN 5613).

*Ichthyomys stolzmanni* (n = 4): Ecuador, Tungurahua, río Zúñac (MEPN 12672). Peru, Jatumpampa (MUSA 18920), Urubamba, Aguas Calientes, Mandorpampa (MUSA 18953, MUSA 18954).

*Ichthyomys tweedii* (n = 1): Ecuador, El Oro, Santa Rosa, Sabayan (MECN 5772).

Genus *Neusticomys* (n = 2)

*Neusticomys monticolus* (n = 1): Ecuador, Napo, Oyacachi (QCAZ 927).

*Neusticomys vossi* (n = 1): Ecuador, Tungurahua, Patate, Los Manteles (MECN 7666).

Tribe Neomicroxini

Genus *Neomicroxus* (n = 2)

*Neomicroxus latebricola* (n = 2): Ecuador, Imbabura, Otavalo, Mojanda (MECN 6927); Carchi, El Angel, Bosque de Polylepis (MECN 3748).

Tribe Oryzomyini

Clade A

Genus *Scolomys* (n = 2)

*Scolomys melanops* (n = 2): Ecuador, Morona Santiago, Shuin Mamus (MECN 5777); Zamora Chinchipe, Bosque Montano Bajo de la Cordillera del Cóndor (QCAZ 16662).

Genus *Zygodontomys* (n = 1)

*Zygodontomys brevicauda* (n = 1): Venezuela, Portuguesa, Colonia Agrícola Turen, S Acarigua (CVULA 194).

Clade B

Genus *Casiomys* (n = 4)

*Casiomys alfaroi* (n = 3): Ecuador, El Oro, Sabayan (MECN 5753); Imbabura, Reserva Río Manduriacu (MECN 8012); Pichincha, Hacienda El Milagro, unión del río Guayllabamba e Intag (QCAZ 10998).

*Casiomys rostratus* (n = 1): Mexico (CMZ 91).

Genus *Euryoryzomys* (n = 2)

*Euryoryzomys macconelli* (n = 1): Ecuador, Sucumbíos, La Balsareña (MECN 3164).

*Euryoryzomys russatus* (n = 1): Argentina, Misiones, Parque Provincial Piñalito, San Pedro (CNP 6602 [CNP-D 106]).

Genus *Hylaeamys* (n = 3)

*Hylaeamys perenensis* (n = 1): Ecuador, Sucumbíos, Reserva de Producción Faunística, Amarumpoza, Río Cuyabeno (QCAZ 12656).

*Hylaeamys tatei* (n = 1): Ecuador, Morona Santiago, Chalwayaku (MECN 7145).

*Hylaeamys yunganus* (n = 1): Ecuador, Orellana, Parroquia Cononaco, Territorio Huaorani, Zona Intangible, 5 km E de Boanamo (QCAZ 13573).

Genus *Mindomys* (n = 1)

*Mindomys hammondi* (n = 1): Ecuador, Carchi, Reserva Drácula (MECN 6228).

Genus *Nephelomys* (n = 4)

*Nephelomys albigularis* (n = 1): Ecuador, El Oro, Chilla (MECN 6976).

*Nephelomys* *auriventer* (n = 1): Ecuador, Tungurahua, Reserva Río Zúñac (MECN 8285).

*Nephelomys* *moerex* (n = 1): Ecuador, Pichincha, Reserva Geobotánica Pululahuax (MECN 4936).

*Nephelomys* sp. (n = 1): Ecuador, Carchi, Golondrinas (JBM 3099).

Genus *Oecomys* (n = 2)

*Oecomys bicolor* (n = 1): Ecuador, Tungurahua, Reserva Río Zúñac (MECN 6374).

*Oecomys franciscorum* (n = 1): Argentina, Chaco, junction between Río de Oro and Ruta Provincial 33 (CNP 5076).

Genus *Pattonimus* (n = 3)

*Pattonimys ecominga* (n = 1): Ecuador, Carchi, Reserva Drácula (MECN 6336).

*Pattonimys musseri* (n = 2): Ecuador, Imbabura, Reserva Río Manduriacu (MECN 8008, MECN 8010).

Genus *Transandinomys* (n = 4)

*Transandinomys bolivaris* (n = 2): Ecuador, Manabí, Reserva Ateles (MECN 8127); Pichincha, Mashpi Logde y Reserva Mashpi (QCAZ 18681).

*Transandinomys talamancae* (n = 2): Ecuador, Esmeraldas, Capulí (QCAZ 9780); Ecuador, El Oro, Santa Rosa (MECN 5768).

Clade C

Genus *Microryzomys* (n = 2)

*Microryzomys altissimus* (n = 1): Ecuador, Tungurahua, La Palmera (MECN 7197).

*Microryzomys minutus* (n = 1): Ecuador, Tungurahua, Los Manteles (MECN 7687).

Genus *Neacomys* (n = 2)

*Neacomys auriventer* (n = 1): Ecuador, Zamora Chinchipe, Paquisha (MECN 7345).

*Neacomys rosalindae* (n = 1): Ecuador, Orellana, Tiputini (MECN 4159).

Genus *Oligoryzomys* (n = 16)

*Oligoryzomys brendae* (n = 3): Argentina, Jujuy, San Francisco (CNP 6646 [CNP-D 173]), Río Jordan y RP 83, San Francisco (CNP 6651 [CNP-D 188]), camino a Alto Calilegua (CNP 7744 [CNP-D 331]).

*Oligoryzomys chacoensis* (n = 4): Argentina, Chaco, Laguna El Riacho, Parque Nacional El Impenetrable (CNP 6837 [CNP-D 190]), Laguna El Breal, Parque Nacional El Impenetrable (CNP 7742 [CNP-D 339]); Salta, Laguna Los Rastrojos, Reserva Natural Privada Palma Chueca, La Unión (CNP 6626 [CNP-D 177]), Palmar de Copernicia, 32 km en línea recta al SW de La Unión (CNP 6839 [CNP-D 179]).

*Oligoryzomys flavescens* (n = 2): Argentina, Buenos Aires, La Balandra (CNP 7521 [CNP-D 37]), Arroyo El Pescado (CNP 7758 [CNP-D 298]).

*Oligoryzomys longicaudatus* (n = 3): Argentina, Río Negro, inmediaciones de Bariloche (CNP 7022 [CNP-D 333], CNP 7023 [CNP-D 338], CNP 7024 [CNP-D 335]).

*Oligoryzomys nigripes* (n = 4): Argentina, Buenos Aires, La Balandra (CNP 7516 [CNP-D 38]); Corrientes, Estancia San Nicolás, 22 km al SE de San Miguel, Departamento San Miguel (CNP 7703 [CNP-D 334); Entre Ríos, Isla Queguay, río Uruguay (CNP 5242 [CNP-D 332]); Misiones, Estancia Santa Inés, Departamento Capital (CNP 6648 [CNP-D 108]).

Genus *Oreoryzomys* (n = 3)

*Oreoryzomys balneator* (n = 3): Ecuador, Morona Santiago, Kutuku (MECN 5857, MECN 5860); Tunguragua, Baños, Vizcaya (MECN 6404).

Clade D

Genus *Aegialomys* (n = 2)

*Aegialomys xanthaeolus* (n = 2): Ecuador, Santo Domingo de los Tsáchilas, Plantabal (MECN 8313); Manabí, El Bejuco (QCAZ 18402).

Genus *Cerradomys* (n = 1)

*Cerradomys vivoi* (n = 1): Brasil, Minas Gerais, Projeto Jaíba, Riacho Mocambinho (MN 34433).

Genus *Holochilus* (n = 16)

*Holochilus brasiliensis* (n = 11): Argentina, Buenos Aires, La Balandra (CNP 5047 [CNP-D 131]), 5 km N Monte Hermoso, sobre el arroyo de las Cortaderas (CNP 5321 [CNP-D 132], CNP 5269 [CNP-D 133], CNP 5314 [CNP-D 134], CNP 5349 [CNP-D 135], CNP 6263 [CNP-D 138], CNP 5321 [CNP-D 156], CNP 5349 [CNP-D 157], CNP 5269 [CNP-D 162]); Entre Ríos, Ruta Nacional 130, km 9, Villa Elisa, Colón (CNP 8616 [CNP-D 15], CNP 6594 [CNP-D 126]).

*Holochilus chacarius* (n = 4): Argentina, Laguna Los Rastrojos, Reserva Natural Privada Palma Chueca, La Unión (CNP 6606 [CNP-D 161], CNP 6645 [CNP-D 165], CNP 7712 [CNP-D 336], CNP 7185 [CNP-D 337]).

*Holochilus nanus* (n = 1): Bolivia, Beni, Lago Rogaguado (CBF 12062).

Genus *Lundomys* (n = 1)

*Lundomys molitor* (n = 1): Brasil, Santa Catarina, PCH Passos Maia, municipality of Passos Maia (UFSC 5005).

Genus *Melanomys* (n = 3)

*Melanomys caliginosus* (n = 2): Ecuador, Carchi, Reserva Drácula (MECN 5968); Cotopaxi, San Francisco de Las Pampas (QCAZ 12117).

*Melanomys colombianus* (n = 1): Venezuela, Mérida, Mirabel 3 km SSE La Azulita (CVULA 1052).

Genus *Nectomys* (n = 2)

*Nectomys apicalis* (n = 1): Ecuador, Morona Santiago, Gualaquiza (MECN 5864).

*Nectomys squamipes* (n = 1): Argentina, Misiones, Parque Provincial Piñalito, San Pedro (CNP 6950 [CNP-D 122]).

Genus *Pseudoryzomys* (n = 3)

*Pseudoryzomys simplex* (n = 3): Brasil, Tocantins, Parque Nacional do Araguaia (MN 60480, MN 60496, MN 66269).

Genus *Sigmodontomys* (n = 2)

*Sigmodontomys alfari* (n = 2): Ecuador, Carchi, Reserva Drácula (MECN 6022); Imbabura, Lita (MECN 6595).

Genus *Sooretamys* (n = 1)

*Sooretamys angouya* (n = 1): Brasil, Santa Catarina, UHE Quebra Queixo, municipality of Ipuaçu (UFSC 6031).

Genus *Tanyuromys* (n = 2)

*Tanyuromys thomasleei* (n = 2): Ecuador, Carchi, Reserva Drácula (MECN 6179); Imbabura, Reserva Río Manduriacu (MECN 6484).

Tribe Phyllotini

Subtribe Calomyina

Genus *Calassomys* (n = 1)

*Calassomys albimaculatus* (n = 1): Brasil, Minas Gerais, Sempre Vivas National Park, 3.25 km by rd NW Macacos, Pedreira do Gaio (CNP 3437 [CNP-D 110]).

Genus *Calomys* (n = 14)

*Calomys fecundus* (n = 1): Argentina, Salta, Palmar de Copernicia, 32 km en línea recta al SW de La Unión (CNP 6806 [CNP-D 178]).

*Calomys frida* (n = 1): Peru, Apurímac, Cotabambas, Challhuahuacho (RCC 472).

*Calomys laucha* (n = 5): Argentina, Buenos Aires, Arroyo El Pescado (UP 5022 [CNP-D 296]), Est. Experimental Olmos (CNP 7614 [CNP-D 68], CNP 7598 [CNP-D 71]), La Rioja, Faimalla (CNP 5049 [CNP-D 207], CNP 5050 [CNP-D 211]).

*Calomys musculinus* (n = 6): Argentina, Pescadero El Edén del Pejerrey, frente al Vivero MAA Chasicó (LTU 79 [CNP-D 201]), EEA INTA Hilario Ascasubi, linde norte (CNP 4633 [CNP-D 389], CNP 5689 [CNP-D 487]); Río Negro, Est. El Cóndor (CNP 3658 [CNP-D 342]), CNP 5686 [CNP-D 453]; Chubut, Puerto Madryn, CENPAT (CNP 8187 [CNP-D 534]).

*Calomys venustus* (n = 1): Argentina, Córdoba, Deán Funes, Campo “La Luisiana” (CNP 5790 [CNP-D 225]).

Subtribe Phyllotina

Genus *Auliscomys* (n = 5)

*Auliscomys boliviensis* (n = 1): Peru, Moquegua, Mariscal Nieto, Torata (RCC 511).

*Auliscomys pictus* (n = 3): Bolivia, La Paz, Puina (CBF 9543). Peru, Apurímac, Cotabambas, Challhuahuacho (RCC 470, RCC 471).

*Auliscomys sublimis* (n = 1): Bolivia, La Paz, Puina (CBF 9563).

Genus *Eligmodontia* (n = 16)

*Eligmodontia hirtipes* (n = 1): Peru, Tacna, Tarata, Kallapuma (RCC 437).

*Eligmodontia moreni* (n = 2): Argentina, Mendoza, Arroyo Chacay y río Mendoza (CNP 7651 [CNP-D 301]), Uspallata (MNT 298 [CNP-D 341]).

*Eligmodontia morgani* (n = 8): Argentina, Santa Cruz, Ea. La Madrugada, Cañadón del Duraznillo, sendero peatonal (CNP 5446 [CNP-D 411], CNP 5416 [CNP-D 413], CNP 5492 [CNP-D 418], CNP 5425 [CNP-D 421], CNP 5454 [CNP-D 423], CNP 5496 [CNP-D 425], CNP 4509 [CNP-D 427]), margen SW Lago Musters (PPA 879 [CNP-D 471]).

*Eligmodontia typus* (n = 5): Argentina, Mendoza, Agua del Toro (CNP 7650 [CNP-D 305], CNP 7825, CNP 7826, CNP 7827 [CNP-D 539], CNP 7828).

Genus *Graomys* (n = 12)

*Graomys chacoensis* (n = 5): Argentina, Córdoba, Parque Provincial Chancani (CNP 4732 [CNP-D 49]); Chaco, Parque Nacional El Impenetrable (CNP 6632 [CNP-D 163]); Salta, Reserva Natural Privada Palma Chueca, La Unión (CNP 6619 [CNP-D 186], CNP 6637 [CNP-D 172]), CNP 6638 [CNP-D 192]).

*Graomys griseoflavus* (n = 7): Argentina, Mendoza, Estación Transformadora 25 de Mayo (CNP 7652 [CNP-D 314]), Diamante River (UP 4163 [CNP-D 328]), Santa Cruz, Ea. La Madrugada, Cañadón del Duraznillo, sendero peatonal (CNP 5411 [CNP-D 438], CNP 5404 [CNP-D 484]); Río Negro, El Espigón, 29 km S Bln. El Cóndor sobre RN 1 (CNP 1670 [CNP-D 502]); Chubut, Puerto Madryn, CENPAT (UP 5032 [CNP-D 551]).

Genus *Loxodontomys* (n = 9)

*Loxodontomys micropus* (n = 9): Argentina, Chubut, Estancia Leleque, La Potrada (CNP 1653 [CNP-D 139]), Estancia El Maitén (CNP 1628 [CNP-D 198]), Lago Fontana, 1 km E RP 57 desde cabaña municipal (CNP 5338 [CNP-D 496]; Río Negro, inmediaciones de Bariloche (CNP 6522 [CNP-D 142], CNP 6523 [CNP-D 141]), Neuquén, lago Traful (MNT 168 [CNP-D 212], MNT 176 [CNP-D 215], MNT 162 [CNP-D 227]); Santa Cruz, Estancia La Ensenada (CNP 1988 [CNP-D 292]).

Genus *Phyllotis* (n = 24)

*Phyllotis andium* (n = 1): Ecuador, Curiquinga, antena (QCAZ 1136).

*Phyllotis bonariensis* (n = 1): Argentina, Buenos Aires, campamento base Sierra de la Ventana (LTU 117 [CNP-D 410]).

*Phyllotis haggardi* (n = 1): Ecuador, Cotopaxi, Río Barrancas, en la falda suroccidental del volcán Cotopaxi (QCAZ 8446).

*Phyllotis pehuenche* (n = 4): Argentina, Mendoza, Portezuelo del Viento, Meseta del Pueblo (UP 4003, UP 4004, UP 4005); Neuquén, Cueva Epullán Grande (CNP 7759 [CNP-D 528]).

*Phyllotis rupestris* (n = 1): Peru, Tacna, Tarata, Kallapuma (RCC 446).

*Phyllotis vaccarum* (n = 8): Argentina, Mendoza, Diamante River (UP 4103 [CNP-D 280], UP 4104 [CNP-D 262], UP 4105 [CNP-D 276], UP 4106 [CNP-D 259], UP 4107 [CNP-D 283], UP 4108 [CNP-D 279], UP 4158 [CNP-D 326]), Uspallata (MNT 290 [CNP-D 350]).

*Phyllotis xanthopygus* (n = 8): Argentina, Chubut, Alm. Hotel Los Manantiales (PPA 898 [CNP-D 424], PPA 888 [CNP-D 426], PPA 949 [CNP-D 431], PPA 901 [CNP-D 442]), extremo W-SW Lago Blanco (CNP 1255 [CNP-D 549]), Santa Cruz, Estancia Cerro Ventana (PNG 791 [CNP-D 29], PNG 799 [CNP-D 230]), Río Chico, La península, 2 km SSW por camino de Tío Camping (PPA 425 [CNP-D 219]).

Genus *Tapecomys* (n = 2)

*Tapecomys primus* (n = 2): Argentina, Jujuy, Peña Alta, RP 83, San Francisco (CNP 6607 [CNP-D 158], CNP 7564).

Tribe Reithrodontini

Genus *Reithrodon* (n = 11)

*Reithrodon auritus* (n = 11): Argentina, Chubut, Estancia El Maitén (CNP 216 [CNP-D 137]), Puerto Piojo (CNP 2290 [CNP-D 295]), Alm. Hotel Los Manantiales (CNP 5441 [CNP-D 415]); Mendoza, Cerro Colorado (CNP 6669 [CNP-D 238]); Río Negro, inmediaciones de Bariloche (CNP 6674 [CNP-D 239]); Santa Cruz, Cabo Vírgenes (CNP 7158 [CNP-D 300]), Lago Posadas, Tío Camping (CNP 6309 [CNP-D 85], CNP 7018 [CNP-D 286], CNP 7019 [CNP-D 288], CNP 7020 [CNP-D 290]).

Tribe Rhagomyini

Genus *Rhagomys* (n = 2)

*Rhagomys rufescens* (n = 1): Brasil, Minas Gerais, Mata do Paraíso (GL 909).

*Rhagomys septentrionalis* (n = 1): Ecuador, Morona Santiago, Río Upano (MECN 6172).

Tribe Sigmodontini

Genus *Sigmodon* (n = 2)

*Sigmodon peruanus* (n = 2): Ecuador, Manabí, Guarango (MECN 4184); Loja, Selva Alegre (QCAZ 2072).

Tribe Thomasomyini

Genus *Aepeomys* (n = 4)

*Aepeomys lugens* (n = 4): Venezuela, Mérida, El Mostrenco, 4 km S de Santo Domingo (CVULA I-1057), 12.5 km SE La Azulita, La Bravera (CVULA I-8069, CVULA I-8070, CVULA I-8071).

Genus *Chilomys* (n = 5)

*Chilomys georgeledecii* (n = 1): Ecuador, Carchi, Reserva Drácula (MECN 6205).

*Chilomys neisi* (n = 1): Ecuador, El Oro, Chilla (MECN 6187).

*Chilomys percequilloi* (n = 2): Ecuador, Tungurahua, Vizcaya (MECN 6097, MECN 6098).

*Chilomys weksleri* (n = 1): Ecuador, Cotopaxi, Reserva Integral Otonga (QCAZ 1787).

Genus *Rhipidomys* (n = 6)

*Rhipidomys austrinus* (n = 1): Argentina, Salta, Parque Nacional El Rey (CNP 827 [CNP-D 143).

*Rhipidomys* cf. *R. latimanus* (n = 2): Ecuador, Carchi, Reserva Drácula (MECN 5934); Cotopaxi, San Francisco de Las Pampas (QCAZ 2437).

*Rhipidomys leucodactylus* (n = 1): Ecuador, Morona Santiago, Chawalyaku (MECN 7147).

*Rhipidomys venezuelae* (n = 1): Venezuela, Mérida, Capazón, La Azulita (CVULA I-1250).

*Rhipidomys* sp. (n = 1): Ecuador, Morona Santiago, Tinajillas (MECN 8125).

Genus *Thomasomys* (n = 29)

Aureus group

*Thomasomys* cf. *T. aureus* (n = 2): Ecuador, Cotopaxi, Reserva Integral Otonga (MECN 6500, QCAZ 2406).

*Thomasomys burneoi* (n = 1): Ecuador, Tungurahua, Guamag (MECN 7263).

*Thomasomys* sp. (aureus group) (n = 2): Bolivia, La Paz, Puina (CBF 9510), Tunquini (CBF 7660).

Baeops group

*Thomasomys baeops* (n = 1): Ecuador, Tungurahua, Manteles (MECN 7694).

*Thomasomys taczanowskii* (n = 2): Ecuador, El Oro, Chillacocha (MECN 6236, MECN 6827).

Cinereus group

*Thomasomys caudivarius* (n = 1): Ecuador, El Oro, Chilla (MECN 7022).

*Thomasomys cinnameus* (n = 1): Ecuador, Tungurahua, Patate (MECN 7685).

*Thomasomys emeritus* (n = 1): Venezuela, Mérida, Monte Zerpa, 6 km N Mérida (CVULA 4918).

*Thomasomys erro* (n = 3): Ecuador, Napo, Reserva Biológica Colonso-Chalupas (MECN 6891, MECN 6892); Carchi, Cordillera de la Virgen Negra (QCAZ 9879).

*Thomasomys fumeus* (n = 2): Ecuador, Napo, Reserva Biológica Colonso-Chalupas (MECN 6893); Tungurahua, Baños (MECN 621).

*Thomasomys hudsoni* (n = 2): Ecuador, Tungurahua, Faldas del Tungurahua (MECN 6089); Loja, Parque Nacional Yacuri, Cantón Espíndola (QCAZ 15644).

*Thomasomys paramorum* (n = 1): Ecuador, Chimborazo, Cubillines (MECN 5277).

*Thomasomys salazari* (n = 1): Ecuador, Morona Santiago, Parque Nacional Sangay (MECN 5681).

*Thomasomys silvestris* (n = 2): Ecuador, Pichincha, Bellavista (MECN 2821); Cotopaxi, Reserva Integral Otonga (QCAZ 2648).

*Thomasomys ucucha* (n = 2): Ecuador, Napo, Papallacta (MECN 2695, MECN 2692).

*Thomasomys vestitus* (n = 1): Venezuela, Mérida, El Baho, 5 km SW Santo Domingo (CVULA 989).

*Thomasomys vulcani* (n = 1): Ecuador, Imbabura, Toisan (MECN 7522).

*Thomasomys* sp. (cinereus group) (n = 1): Ecuador, Zamora Chinchipe, Área Ecológica de Conservación Municipal Yacuambi, Urdaneta, Páramos de Gurudel (QCAZ 13300).

Gracilis group

*Thomasomys andersoni* (n = 1): Bolivia, La Paz, Cargadero (CBF 9874).

Incanus group

*Thomasomys* cf. *T. ladewi* (n = 1): Bolivia, La Paz, Chullo (CBF 10209).

Tribe Wiedomyini

Genus *Juliomys* (n = 4)

*Juliomys ossitenuis* (n = 2): Brasil, Paraná, municipality of São Mateus do Sul, not precise locality (UFSC 5949), Rio de Janeiro, Vassouras, Fazenda Leonardo Parente (MN 80630).

*Juliomys pictipes* (n = 2): Argentina, Misiones, Arroyo de Salamanca, Reserva Natural Ernesto Che Guevara (CNP 895). Brasil, Santa Catarina, Caldas da Imperatriz, municipality of Santo Amaro da Imperatriz (UFSC 5874).

Genus *Phaenomys* (n = 1)

*Phaenomys ferrugineus* (n = 1): Brasil, Minas Gerais, Itamonte, Hotel Casa Alpina (CMUFLA 904).

Genus *Wiedomys* (n = 3)

*Wiedomys cerradensis* (n = 1): Brasil, Rio Grande do Norte, Assú (UFSC 6191).

*Wiedomys pyrrhorhinos* (n = 2): Brasil, Bahia, Brotas de Macaúbas (UFSC 5224); Minas Gerais, Parque Florestal Jaíba (MN 62181).

Genus *Wilfredomys* (n = 2)

*Wilfredomys oenax* (n = 1): Uruguay (CNP-D 130).

*Wilfredomys* sp. (n = 1): Brasil, Santa Catarina, PCH Passos Maia, municipality of Passos Maia (UFSC 5614).

Sigmodontinae *incertae sedis*

Genus *Abrawayaomys* (n = 1)

*Abrawayaomys chebezi* (n = 1): Argentina, Misiones, Parque Provincial Urugua-í (CNP 3631 [CNP-D 56]).

Genus *Chinchillula* (n = 2)

*Chinchillula sahamae* (n = 2): Bolivia, La Paz, Puina (CBF 9539). Peru, Tacna, Tarata, Kallapuma (RCC 434).

Genus *Delomys* (n = 3)

*Delomys dorsalis* (n = 2): Brasil, Rio de Janeiro, Nova Friburgo, Três Picos, Salinas (MN 80411, MN 80413).

*Delomys sublineatus* (n = 1): Brasil, Santa Catarina, Caldas da Imperatriz, municipality of Santo Amaro da Imperatriz (UFSC 5908).
