# Supplementary figures and images for: Gastric morphology in sigmodontine rodents (Mammalia: Cricetidae): a comprehensive comparative classification
Source: PeerJ. 2026 Jun 10;14:e21405. doi: 10.7717/peerj.21405 (PMC13264281; doi:10.7717/peerj.21405)

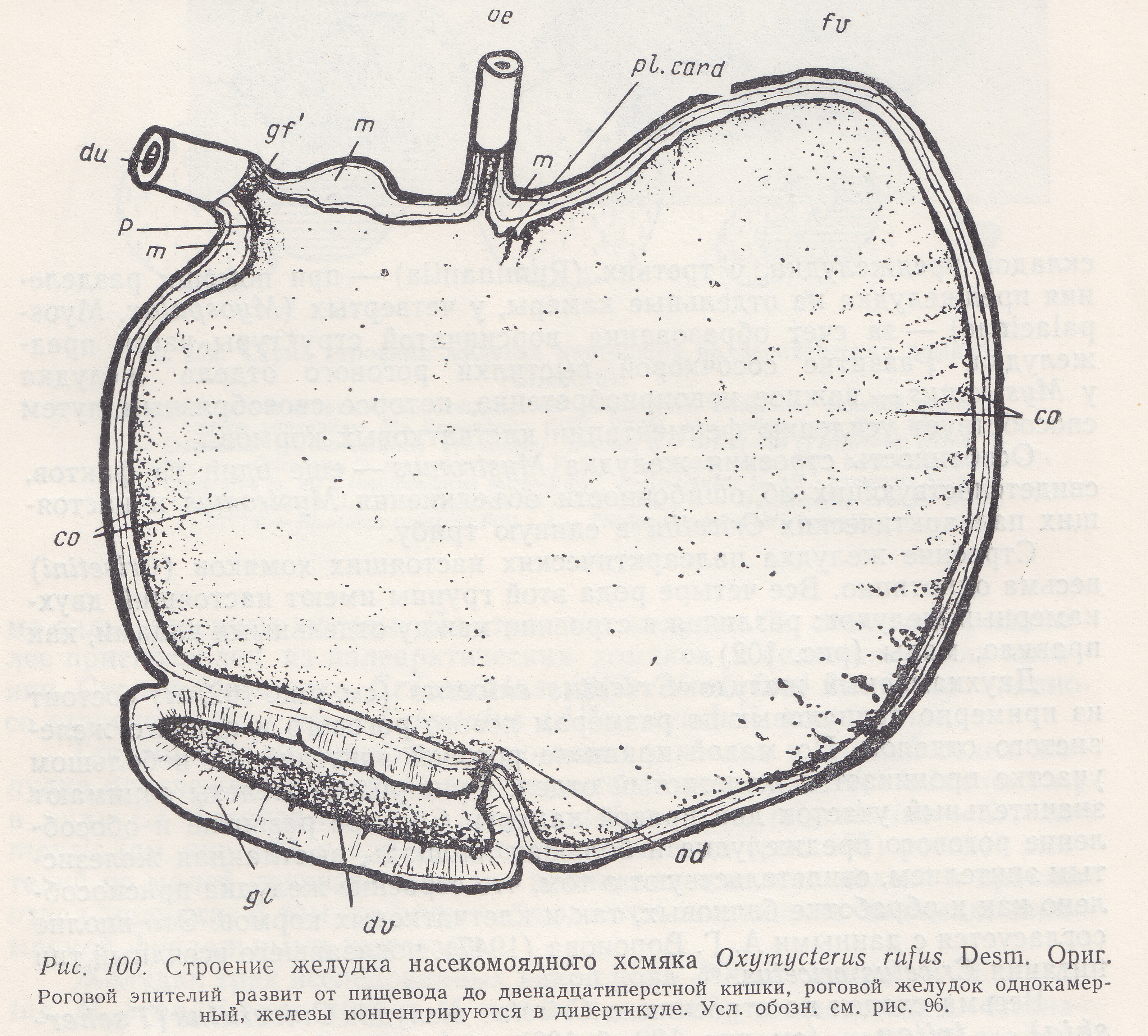

Supplement: Supplemental Information 4 — Original illustration of the stomach of Oxymycterus rufus from Vorontsov (1967: fig. 100). [file peerj-14-21405-s004.png]

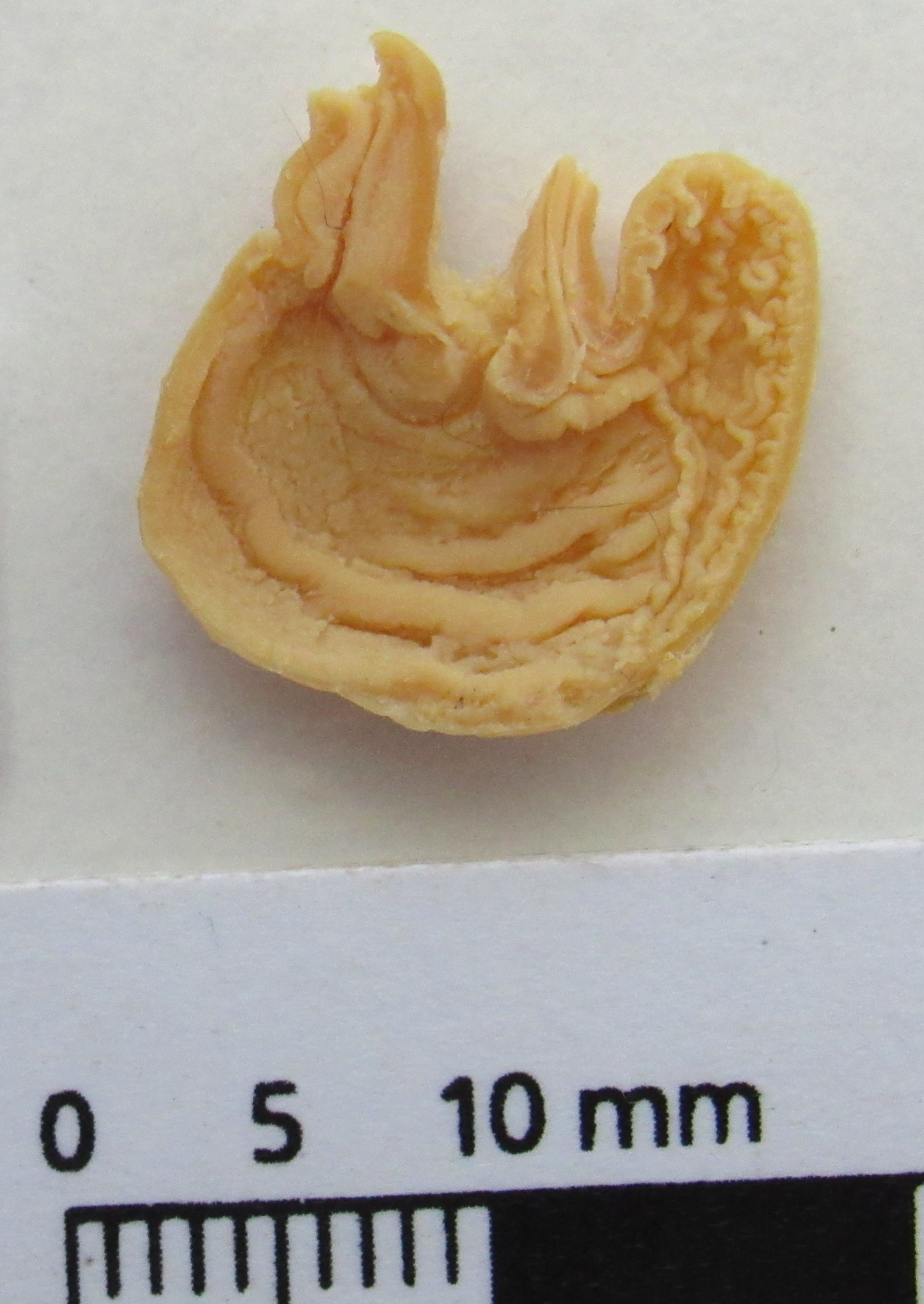

Supplement: Supplemental Information 5 — Necromys obscurus, internal view of the stomach (ventral aspect) based on specimen CNP 6035. [file peerj-14-21405-s005.png]
